# Lessons learned about the biology and genomics of *Diaphorina citri* infection with *“Candidatus* Liberibacter asiaticus” by integrating new and archived organ-specific transcriptome data

**DOI:** 10.1093/gigascience/giac035

**Published:** 2022-04-28

**Authors:** Marina Mann, Surya Saha, Joseph M Cicero, Marco Pitino, Kathy Moulton, Wayne B Hunter, Liliana M Cano, Lukas A Mueller, Michelle Heck

**Affiliations:** Plant Pathology and Plant-Microbe Biology Section, School of Integrative Plant Science, Cornell University, Ithaca, NY 14853, USA; Boyce Thompson Institute, Ithaca, NY 14853, USA; School of Plant Sciences, University of Arizona, Tucson, AZ 85721, USA; AgroSource, Inc. Juniper, FL 33469, USA; U.S. Horticultural Research Laboratory, Unit of Subtropical Insects and Horticulture, USDA Agricultural Research Service, Fort Pierce, FL 34945, USA; U.S. Horticultural Research Laboratory, Unit of Subtropical Insects and Horticulture, USDA Agricultural Research Service, Fort Pierce, FL 34945, USA; Indian River Research and Education Center, University of Florida, Fort Pierce, FL 34945, USA; Boyce Thompson Institute, Ithaca, NY 14853, USA; Plant Pathology and Plant-Microbe Biology Section, School of Integrative Plant Science, Cornell University, Ithaca, NY 14853, USA; Boyce Thompson Institute, Ithaca, NY 14853, USA; Emerging Pests and Pathogens Research Unit, Robert W. Holley Center, USDA Agricultural Research Service, Ithaca, NY 14853, USA

**Keywords:** Diaphorina citri, Huanglongbing, *Candidatus* Liberibacter asiaticus, transcriptomics, citrus, vector-pathogen interactions

## Abstract

**Background:**

Huanglongbing, a devastating disease of citrus, is caused by the obligate, intracellular bacterium *“Candidatus* Liberibacter asiaticus” (*C*Las). *C*Las is transmitted by *Diaphorina citri*, the Asian citrus psyllid. Development of transmission-blocking strategies to manage huanglongbing relies on knowledge of *C*Las and *D. citri* interactions at the molecular level. Prior transcriptome analyses of *D. citri* point to changes in psyllid biology due to *C*Las infection but have been hampered by incomplete versions of the *D. citri* genome, proper host plant controls, and/or a lack of a uniform data analysis approach. In this work, we present lessons learned from a quantitative transcriptome analysis of excised heads, salivary glands, midguts, and bacteriomes from *C*Las-positive and *C*Las-negative *D. citri* using the chromosomal length *D. citri* genome assembly.

**Results:**

Each organ had a unique transcriptome profile and response to *C*Las infection. Though most psyllids were infected with the bacterium, *C*Las-derived transcripts were not detected in all organs. By analyzing the midgut dataset using both the Diaci_v1.1 and v3.0 *D. citri* genomes, we showed that improved genome assembly led to significant and quantifiable differences in RNA-sequencing data interpretation.

**Conclusions:**

Our results support the hypothesis that future transcriptome studies on circulative, vector-borne pathogens should be conducted at the tissue-specific level using complete, chromosomal-length genome assemblies for the most accurate understanding of pathogen-induced changes in vector gene expression.

## Background

Huanglongbing (HLB), also known as citrus greening, is the most serious disease of citrus (reviewed in [1–3]). HLB symptoms include leaves with blotchy, chlorotic mottling, stunting, loss of root biomass, premature fruit drop, uneven fruit development, and ultimately tree death. In the USA and Asia, HLB is associated with plant vascular tissue infection by the gram-negative, uncultivable Alphaproteobacteria “*Candidatus* Liberibacter asiaticus” (*C*Las). The Asian citrus psyllid *Diaphorina citri* Kuwayama (NCBI:txid121845) (Hemiptera: Liviidae) is the vector of *C*Las. HLB has decimated a multi-billion dollar industry in Florida and is threatening the industries in Texas and California [4].

**Figure 1:**
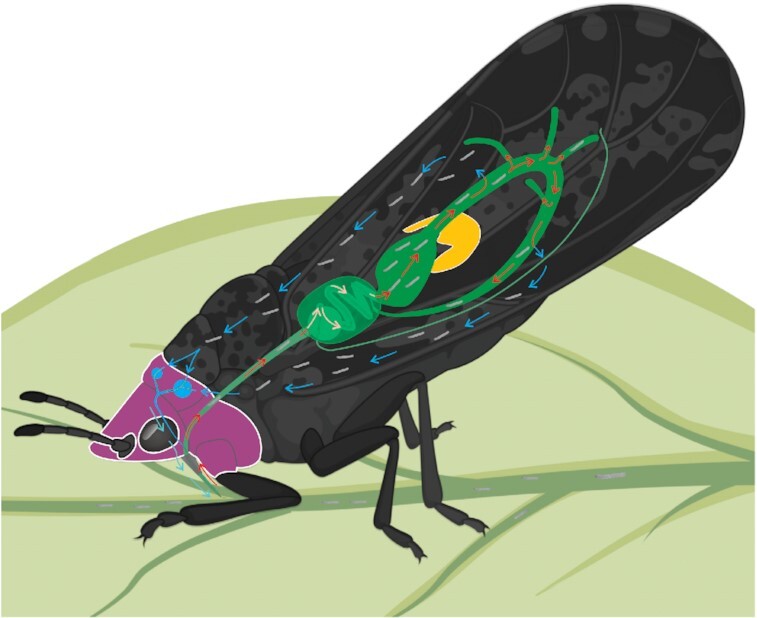
Schematic of *Diaphorina citri* on a citrus leaf, showing the anatomical location and physical details of four organs that were extracted from adult specimens to create four datasets (gut—green, bacteriome—yellow, salivary gland—blue, head—dark purple). The circulative transmission of “*Candidatus* Liberibacter asiaticus” (*C*Las, represented by small grey lines and blue arrows) is represented as *C*Las travels from leaf veins through the gut, crossing the midgut epithelial cell layer to circulate in the body of *D. citri. C*Las enters the salivary gland, where it is known, by contributory effects from acquisition by late-instar nymphs, to replicate to high levels, at which point it can be inoculated into the phloem while adult *D. citri*feed (see 3D imaging and digital video by Alba-Tercedor et al. for more details [40]).

Evidence thus far on *C*Las transmission by *D. citri* is consistent with a circulative, propagative transmission mode that is inextricably linked to the insect's development and intracellular environment surrounding *C*Las bacteria (Fig. 1) [5]. During the circulative propagative transmission cycle of *C*Las, *D. citri* acquire *C*Las from an infected citrus host during phloem ingestion as early as the second nymphal instar [6] but in increasing amounts during the fourth and fifth instars of the nymphal stage [7, 8]. The bacteria remain associated with the insect during molting [7, 9, 10]. *C*Las circulates throughout the body of *D. citri* until it reaches the salivary gland tissues, where it replicates to high levels in the adults [11–14]. Approximately 30% of the *C*Las population replicates in the psyllid [15], primarily in the salivary gland tissue, over ∼1–2 weeks [7]. The infected adults are then competent vectors capable of tree-to-tree spread of *C*Las. *C*Las is detectable in the insect's alimentary canal, especially the midgut [11, 12, 16]. The bacteria also systemically infect the psyllid during propagative transmission, including the hemolymph, salivary glands, muscle, fat body, and reproductive organs (reviewed in [3]). Specific cellular receptors in these different *D. citri* tissues are not known. In adults, *C*Las forms a biofilm along the midgut and induces apoptosis of midgut epithelial cells [17], a process that is not observed in nymph midguts [18]. In the midgut, the bacterium is hypothesized to be associated with the endoplasmic reticulum on the basis of microscopic observations [19]. The movement and infection of *C*Las in the vector tissues predicts extensive vector-pathogen interactions at the molecular level. We use the *C*Las (+) and *C*Las (−) designation to refer to the different sample groups, where the *C*Las (+) insects were reared on *C*Las-infected trees and the *C*Las (−) insects were reared on healthy citrus that also tested negative for *C*Las by quantitative PCR (qPCR).

*D. citri* harbors 3 bacterial symbionts, “*Candidatus* Profftella armatura,” “*Candidatus* Carsonella ruddii,” and *Wolbachia pipientis*[20–26], which reside in a specialized organ referred to as the bacteriome. The bacteriome is composed of bacteriocytes—psyllid cells densely packed with the endosymbiotic bacteria. The bacteriome of *D. citri* has a precise and elegant cellular organization that has been described using fluorescence microscopy [20, 24]. *Carsonella* resides in the outer bacteriocytes and *Profftella* resides in the internal syncytial cytoplasm of the bacteriome. The functions of these beneficial bacterial symbionts in the biology of *D. citri* are inferred from bacterial genome sequencing, proteomics, metabolite, and quantitative PCR (qPCR) data [20].

Rapid advances in genome sequencing technologies have paved the way to a deeper understanding of vector biology over the past decade, including in the analysis of the *D. citri* genome sequence [22, 27–30]. The short-read–based assembly Diaci_v1.1 [29, 31] has been foundational tomany published research articles on *D. citri* to date, including the newest chromosomal length reference genome [30], which is expected to lend more reliability and contain more cohesive, full-length annotated gene models. Numerous studies have used these valuable *D. citri* genome sequencing resources to investigate interactions between *D. citri* and *C*Las and *D. citri* biology at the transcriptome and proteome levels [16, 32–36]. Wu and colleagues [37] published a thorough RNAseq experiment including an analysis of organs, sexes, and life stages of *D. citri*. Their analysis focused on potential insecticide detoxification genes from *C*Las (−) insects raised on a close relative of citrus known to be resistant to systemic infection by *C*Las, *Murraya exotica*, but did not address the impact of *C*Las infection in these organs. A year later, the same group published a paired transcriptome-proteome article focusing on *C*Las (−) *D. citri* salivary glands and associated salivary secretions [38]. They focused on identifying bioactive molecules from the saliva and salivary gland ‘omics analysis and discussed proteins that were found uniquely in the salivary glands from *D. citri* reared on healthy plants.

Tissue-specific omics analyses enable a molecular snapshot of *C*Las–*D. citri* interactions within specific tissues known to be colonized by *C*Las in the insect. Studies have revealed stark differences in patterns of expression when comparing tissue-specific responses to whole-body responses [16, 34]. However, earlier studies were limited in the interpretation of the data because of the incomplete nature of the *D. citri* genome used as a backbone for the quantitative analysis and the application of different computational workflows to identify differentially expressed (DE) genes. Kruse et al. did a thorough analysis and discussed the midgut transcriptomics responses to *C*Las using four biological replicates of pools of hundreds of midguts and performed dual differential expression analysis using two types of computational biology tools, edgeR and DESeq2, to reduce the false discovery rates [16]. However, the results were dependent on paired proteomics and transcriptomics that were both aligned to the relatively low quality and incomplete v1.1 *D. citri* genome, the assembly available at the time. Yu and colleagues [39] built on the study by Kruse et al. [16] using the *D. citri* v2.0 genome, which also lacked the Hi-C scaffolding included in the newest v3.0 genome. Despite the limitations of the genome sequences used for the analyses of these transcriptomes, the results clearly showed that *C*Las has different effects on metabolic pathways expressed within different tissues of *D. citri*. To understand the nature of the *C*Las–*D. citri* relationship at the molecular level, a holistic approach that both integrates the responses across different tissues involved in the circulative transmission pathway and quantifies the impact of *C*Las infection on the transcriptional regulation within specific tissues is necessary.

In this work, we report a comparative, tissue-specific transcriptome analysis of *C*Las (−) to *C*Las (+) psyllid bacteriomes, salivary glands, and heads. Using the newest *D. citri* genome assembly (v3.0), which includes chromosomal-length scaffolds [30], we analyzed these new data together with previously published *C*Las (+) and *C*Las (−) midgut transcriptome data [16]. This study advances our understanding of *D. citri*–*C*Las interactions because it integrates an analysis of new transcriptome data with previously published transcriptome data to show the impact of *C*Las on the transcriptional landscape of *D. citri* organs involved in the circulative, propagative transmission using the latest genomic resources. The lessons learned from and difficulties of comparing the four datasets — three of which were collected from separate insect colonies, at different times, sequenced separately, stored in freezers for different lengths of time, and contain variable amounts of *C*Las in each tissue type — should be acknowledged. This study does not purport to have controlled for all differences found between these datasets, but we do attempt to carefully explain results within the bounds of our controls and include caveats for the confounding effects and the lessons learned from this analysis.

## Data Description

### Background and purpose of data collection

We collected new transcriptomics data from bacteriomes, salivary glands, and heads, as well as using previously published data on the midgut, (i) to test the hypothesis that analysis using newer genome versions can provide valuable new data and (ii) to explore organ-specific patterns of gene expression during *C*Las infection of the insect vector.

### General methods of collection, curation, and quality control

To conduct this study, pools of adult *D. citri* were collected from multiple separate colonies located at the United States Department of Agriculture (USDA) Agricultural Research Service (ARS) in Ithaca, NY, and the USDA ARS in Fort Pierce, FL. Colonies in both laboratories were raised using the same plant growth conditions and on the same host plant species, *Citrus medica—*citron. Psyllid colonies used in this study were either *C*Las-infected (designated *C*Las [+]) or not exposed to the bacterium (raised on *C*Las-negative citrus trees, designated as *C*Las [−]). Sequenced samples were pools of multiple individuals (120 per bacteriome and head replicate [Ithaca colony], 150 per salivary gland replicate [Fort Pierce colony], 250 per midgut replicate [Fort Pierce colony], see [16]). See Table 1 for details of each dataset used in this study.

**Table 1:** Metadata on each of the four datasets used in this study, specifically highlighting the ways each dataset differs from the next; following sequencing, all data were treated to the same methods

Tissue type	No. biological replicates	Time at −80 °C	No. psyllids pooled/replicate	Colony location	RNA extraction
Bacteriome	5 *C*Las (+), 5 *C*Las (−)	6 months	120, 120	Ithaca, NY	Qiagen RNeasy
Head	5 *C*Las (+), 5 *C*Las (−)	6 months	120, 120	Ithaca, NY	Qiagen RNeasy
Salivary gland	4 *C*Las (+), 4 *C*Las (−)	Replicates 1–3, 1 yearReplicates 4, 2 year	150, 150	Fort Pierce, FL	TRIzol
Midgut [16]	3 *C*Las (+), 3 *C*Las (−)	<1 month	250, 250	Fort Pierce, FL	TRIzol

### Accessing the data

Bacteriome, head, and salivary gland samples were sequenced separately from the previously published midgut samples [16]. Raw data have been uploaded to NCBI and are accessible via BioProject accession No. PRJNA385527.

## Analyses

### Although most psyllids were infected with *C*Las, *C*Las-derived RNAseq reads were not detected in all organs

Using qPCR analysis of whole insects, we determined the *C*Las percent infection of the *D. citri* populations used for dissections. Across all sample types, the percent infection was in the range 73–85%. Quantitative cycle (Cq) values <40 were counted as *C*Las (+) (Table 2). In addition to the population-level assessment of *C*Las infection, we quantified *C*Las-mapped reads found within each sample after sequencing (Table 2 and Supplementary Fig. S1). Read counts mapping to the *C*Las-psy62 genome (genome produced from a single psyllid in FL [41]) were detected above background in *C*Las (+) salivary gland and head samples (a mean of 1,965 and 2,681 reads, respectively), suggesting that some AT-rich sequences were captured during poly-A enrichment. Upon closer analysis of the *C*Las-aligning reads from the salivary glands, when ≥3 biological replicates had a transcript with ≥1 read, 50 unique *C*Las messenger RNA (mRNA) transcripts were represented, with an additional six ribosomal RNA (rRNA) transcripts (three of each 16S and 23S transcripts), for a total of 56 *C*Las-psy62 transcripts identified. The majority of *C*Las reads from the salivary glands aligned to the top ten transcripts, where the total number of reads across all biological replicates of each transcript ranged from 80 to 290. Of these top ten, three were listed as “protein coding” and annotated as flgB, flgC, and parB, while the rest were unlabeled/unknown (Supplementary Table S1).

**Table 2:** Percent infection by *C*Las in different *Diaphorina citri* tissues as measured by qPCR, and the mean number of RNAseq reads that aligned to the *C*Las genome (psy62) from each dataset

Dataset		Mean ***C*Las reads** ^4^	% Infection^5^
Midgut**^1^**	*C*Las (−)	1	0
	*C*Las (+)	212	82
Salivary gland**^2^**	*C*Las (−)	0.5	0
	*C*Las (+)	1,965.5	73
Bacteriome**^3^**	*C*Las (−)	0.8	0
	*C*Las (+)	3.4	85
Head**^3^**	*C*Las (−)	174.6	0
	*C*Las (+)	2,681.8	85

1 qPCR Cq data from Kruse et al. [16]; reads aligning to *C*Las are from our own alignments.

2 Salivary glands from a colony with a high (>90%) infection rate.

3 Bacteriomes and heads were taken from the same insects, and whole insects were used for qPCR of *C*Las titer, so the mean Cq value is the same for both datasets.

4 Low read counts may represent sequences from contaminating *C*Las sequences remaining within the *D. citri* genome (which need to be removed), or sequences transferred to *D. citri*, or found in common in other bacterial symbionts present.

5 Cq values of 40 translate to 0 titer of the target bacterium. Cq values are calculated using 20–30 whole-body individuals from each parent colony of each dataset. All Cq <40 are counted for percent infection.

### Global assessment of four *D. citri* transcriptomic datasets reveals an organ-specific response to *C*Las

Across all four datasets, we obtained an average of 27.23 million high-quality reads (midguts: 26.43 M, salivary glands: 44.98 M, bacteriomes: 22.11 M, heads: 15.40 M), and 71.3% of the reads aligned concordantly to the v3.0 *D. citri* genome on average (mean concordant alignment in midguts: 74.17%, salivary glands: 73.51%, bacteriomes: 81.12%, heads: 56.43%). The head dataset proved to be more variable as compared to the other datasets, recording the fewest raw reads and the lowest mean percent alignment. In contrast, the highest percent alignment to the *D. citri* genome was recorded by the bacteriome dataset, samples of which were collected from the same individual insects as the head dataset (Supplementary Table S2).

**Figure 2:**
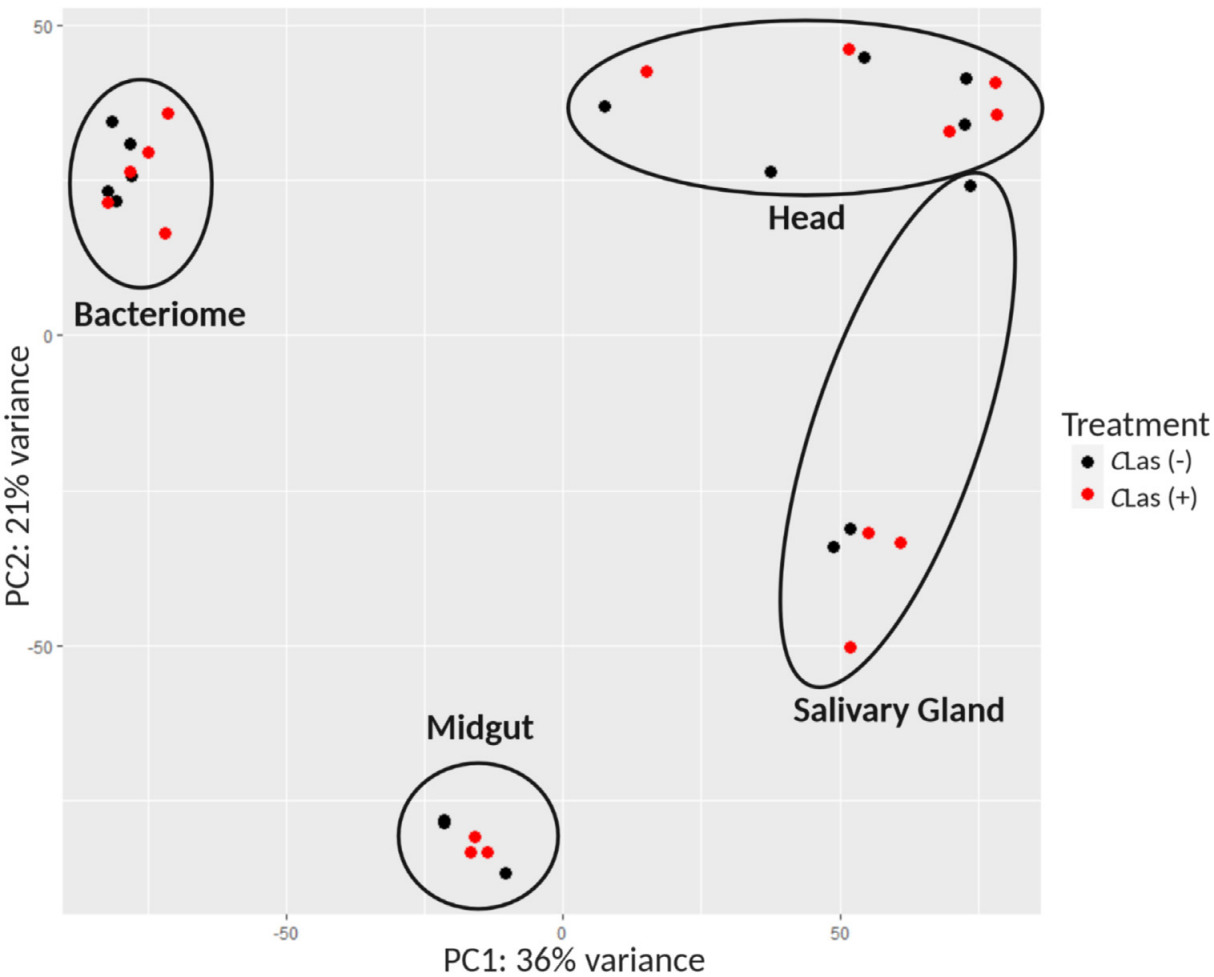
Principal component analysis (PCA) of four *Diaphorina citri* mRNAseq datasets (head, midgut, salivary gland, and bacteriome), each composed of *C*Las (+) and *C*Las (−) biological replicates, showing the two main sources of variation among them. PC1 (36%) separates samples containing salivary tissues (head and salivary gland samples) from the other datasets, while PC2 (21%) distinguishes the bacteriome and head datasets (which were collected in parallel from the same individual insects) from the salivary gland and midgut datasets (which were collected independently). Raw read counts were processed by DESeq2 using the Benjamini-Hochberg normalization method before generating the principal component plot.

A principal component analysis (PCA) to examine the sources of variation among the four *D. citri* dataset expression profiles was performed, where each dataset includes both *C*Las (+) and *C*Las (−) biological replicates. Each organ separated from the other organs in PCA space, showing that each organ has a unique transcriptome profile. The largest source of variation (PC1 = 36%) was explained by differences in the transcriptome profiles of the midgut and bacteriome as compared to the salivary gland and head (Fig. 2). The second largest source of variation between the four datasets (PC2 = 21%) was explained by differences between the midgut and the bacteriome datasets, with a smaller amount of variation between those samples and the head and salivary gland datasets along the same principal component. Importantly, biological replicates of each dataset clustered together and separately from the others (Supplementary Fig. S2), supporting the hypothesis that each organ has a unique transcriptomic signature independent of *C*Las infection. A closer examination of the four clusters showed that the salivary gland, bacteriome, and head datasets did not differentiate between *C*Las (−) and *C*Las (+) biological replicates (Supplementary Fig. S2B–D), while midguts (Supplementary Fig. S2A) showed a clear separation along PC1 between *C*Las (−) and *C*Las (+) biological replicates.

PCA plots of each organ transcriptome dataset revealed other sources of variation (Supplementary Fig. S2). The variance described by PC1 of the salivary gland dataset (44.1%, Supplementary Fig. S2B) was explained by two samples that were kept in the −80°C freezer and then sequenced a year after the other six samples, while PC2 (19%, Supplementary Fig. S2B) represented the effect of *C*Las infection, which is not distinct, except for the two outlier samples. The bacteriome dataset (Supplementary Fig. S2C) showed some separation between *C*Las (+) and *C*Las (−) biological replicates (PC2 = 15.9%) but the majority of variation was due to variance among individual biological replicates (PC1 = 16.7%). The head dataset (Supplementary Fig. S2D) showed similar variation across all samples as the bacteriome dataset. This variation explained both the first and second major sources of variance (PC1 = 39.7%, PC2 = 27.3%) with no obvious distinctions between *C*Las (+) and *C*Las (−) biological replicates.

### Gene expression signatures in response to *C*Las infection are tissue-specific in *D. citri*

Differentially expressed transcripts expressed in *C*Las (+) or *C*Las (−) replicates in addition to transcripts that were present in but differentially expressed between *C*Las (+) and *C*Las (−) biological replicates using the maximum adjusted *P* value of 0.05 and a log_2_ fold change (L2FC) of >|2| were used for downstream analyses. This strict quality and DE threshold limited the number of final transcripts to a small number (midgut = 196, salivary gland = 105, bacteriome = 113, head = 10) (see Supplementary Tables S3–S6 for the list of transcripts). A skew towards up-regulated transcripts in *C*Las (+) biological replicates was detected in all organs (salivary gland: up-regulated = 91, down-regulated = 14; midgut: up-regulated = 129, down-regulated = 67; bacteriome: up-regulated = 70, down-regulated = 43; head: up-regulated = 6, down-regulated = 4).

**Figure 3:**
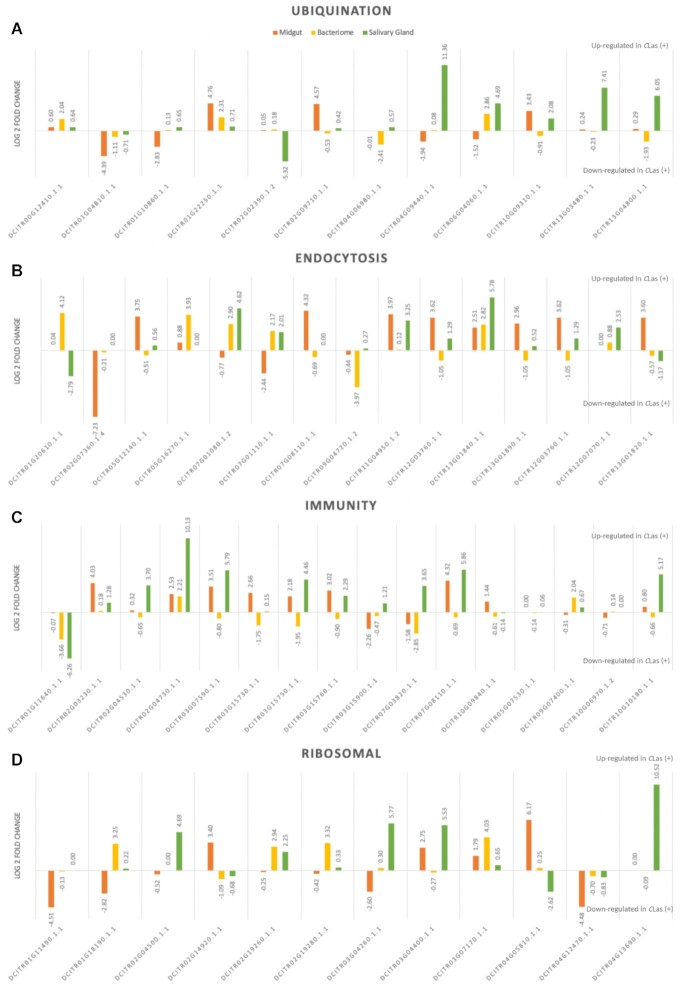
Transcripts have unique expressions across different organs of *C*Las (+) *D. citri*. The top differentially expressed (DE) transcripts from each dataset (bacteriome, midgut, and salivary gland) are sorted by major functional groups including ubiquination, endocytosis, immunity, and ribosomal-related transcripts. Not all transcripts are statistically DE; A transcript may be DE in one dataset but not the others. See Supplementary Table S7 for *P* values.

Four major groups of transcripts were chosen on the basis of their strong representation among the top differentially expressed gene (DEG) lists from the salivary gland, bacteriome, and midgut datasets (Fig. 3, Supplementary Table S7 for a more detailed analysis) to highlight the tissue-specific patterns of transcriptional activation in response to *C*Las. The four groups include ribosomal transcripts, immunity-related transcripts, endocytosis-related transcripts, and ubiquination-related transcripts. Each dataset varies in its magnitude of response (as measured by L2FC and the relative number of transcripts found in each of the four categories). Ubiquination genes are highly up-regulated in the salivary gland dataset (Fig. 3A, green). Endocytosis genes are highly up-regulated in all tissue datasets (Fig. 3B). Immunity genes are up-regulated in the salivary glands and midguts but not the bacteriomes (Fig. 3C, green and orange vs yellow). Different ribosomal genes are up-regulated in *C*Las (+) samples in all three datasets (Fig. 3D), despite ribosomal transcript depletion *in silico*.

In addition to the major patterns (Fig. 3), selected transcripts of interest also showed notable changes in expression in the datasets consistent with the functions of these tissues in *D. citri* physiology that may give insight into how *C*Las is interacting with these specific tissues at the molecular level. These changes are discussed here.

#### Midgut

The top DE transcripts from the midgut dataset were manually sorted into five additional functional categories including biosynthesis and catabolism (n = 55, 40 up-regulated in *C*Las [+], 15 down-regulated), cell structure and signaling (n = 66, 38 up, 28 down), stress (n = 10, 6 up, 4 down), transport (n = 28, 19 up, 9 down), and unknown (n = 37, 26 up, 11 down). The full list can be found in Supplementary Table S3. DE transcripts in the stress category include heat shock and cold shock protein genes, thioredoxin, and E3 ubiquitin ligase. Three heat shock proteins (70-A1, 70-B, 70) are up-regulated with exposure to *C*Las, while the cold shock protein is down-regulated. An E3 ubiquitin ligase, a type IV collagenase, and a *D. citri* homologue of p53 are also up-regulated. A thioredoxin transcript and an HSP20-like chaperone transcript are down-regulated with exposure to *C*Las. Transport-related transcripts that are up-regulated with *C*Las infection include two odorant-binding protein transcripts, membrane-associated ion transporters (aquaporin, major facilitator, protein-coupled AA-transporter, efflux system protein transcript, phosphate transporter, potassium channel protein transcript, and general secretion pathway transcripts), a vacuolar-sorting protein transcript, and an intraflagellar transport particle protein transcript, among others. Down-regulated transcripts include syntaxin, ubiquinol cytochrome-c, membrane-associated proteins and transporters, and nuclear transport factor 2.

#### Salivary gland

The full list of statistically significant, adjusted *P* values (*P*adj <0.05) (Benjamini-Hochberg correction of *P* value via DESeq2) of the salivary gland DE (L2FC >|2|) transcripts can be found in Supplementary Table S4. Transcripts for 40S and 60S subunits of the eukaryotic ribosome are highly up-regulated (40S S15a L2FC = 10.12, 40S S28 L2FC = 10.52, 60S L2FC = 5.53), as well as six transcripts involved with transport which are all up-regulated (ABC transporter C family L2FC = 5.95, α-tocopherol transfer protein L2FC = 8.04, γ-glutamylcyclotransferase L2FC = 8.15, geranylgeranyl transferase L2FC = 6.22, MFS-type transporter L2FC = 4.03, and phosphate acetyltransferase L2FC = 9.30). Additionally, four elongation factor (EF) transcripts are highly up-regulated (EF-1b, EF-2, EF-4, and a calcium-binding EF hand), consistent with increased ribosomal activity. While ubiquination-related transcripts are present in every dataset, in the salivary gland dataset two transcripts are highly up-regulated including a ubiquitin conjugating enzyme (L2FC = 3.70) and ubiquitin-ligase E3 (L2FC = 4.69) [39].

Because the salivary gland is a secretory organ, the most abundant transcripts were checked both for the presence of transmembrane helices (TMHs) and for signal sequences, the first step towards computationally identifying secreted effectors that would modulate interactions between *D. citri* and the citrus host plant during *C*Las transmission. A total of 12 candidate *D. citri* secreted effectors were found: five lack annotation or are otherwise *D. citri*–specific, and four were predicted to contain a TMH. Of the eight candidate salivary gland effector transcripts without TMHs, seven are highly up-regulated in *C*Las (+) adult *D. citri*, while one of the unknown transcripts is highly down-regulated in *C*Las (+) adult salivary glands (Supplementary Table S8). A study by Yu and Killiny [42] studied proteins of *D. citri* saliva, and while no candidates overlap exactly with transcripts identified in this study, many were of similar nature (including serine/threonine kinases, RNA polymerase-associated proteins, ribosomal proteins, homeobox proteins, and ubiquitins). A more recent article by Wu et al. [38] also looked closely at salivary proteins and transcripts from *C*Las (−) *D. citri*, and of the eight possible effectors identified by this study, only the serine proteases were found in common, suggesting that the diversity of secreted effectors is vast, may be context dependent, and requires additional study.

#### Bacteriome

A key group of DE bacteriome transcripts include transporters, methyltransferases, acetyltransferases, and the PiggyBac transposable elements. Three methyltransferases are all highly up-regulated in the *C*Las (+) adult bacteriome (methyltransferase family protein L2FC = 7.48, phthiotriol dimycocerosates methyltransferase L2FC = 5.48, and protein arginine N-methyltransferase L2FC = 2.10) and one acetyltransferase is down-regulated (histone acetyltransferase catalytic subunit L2FC = −2.17). Five transcripts are annotated as “transporters” including three that are up-regulated in *C*Las (+) (cation-chloride cotransporter L2FC = 3.07, cationic amino acid transporter L2FC = 8.43, major facilitator transporter L2FC = 5.59) and two that are down-regulated in *C*Las (+) (ABC transporter G family protein L2FC = −2.13 and organic solute transporter ostalpha protein L2FC = −2.31). Three ribosomal-related transcripts are up-regulated in the *C*Las (+) adult *D. citri* bacteriome (60S L26 with L2FC = 4.03, 60S L37a with L2FC = 3.25, and ribosomal protein L23 with L2FC = 2.93). The full list of statistically significant (*P*adj <0.05) bacteriome DE (L2FC >|2|) transcripts can be found in Supplementary Table S5.

#### Head

The head dataset had relatively few reads sequenced and likewise, very few transcripts were statistically significantly DE. Of the 10 with *P*adj <0.05 and L2FC >|2|, half (n = 5) were associated with cell structure and signaling, including a vigilin gene with L2FC = −4.09, a DNA-polymerase gene with L2FC = −3.42, a Rho-GTPase with L2FC = 5.28, a neuromodulin gene with L2FC = 5.27, and an insulin-like growth factor with L2FC = 5.63. One transcript was associated with activation of autophagy, p53-inducible nuclear protein 1 with L2FC = 5.61). Two were annotated to have transport functions, an ATP synthase subunit gene with L2FC = −5.33, and one with an intracellular protein transport protein with L2FC = 3.41. Two were not functionally annotated (Dcitr10g06500.1.1 with L2FC = 5.56, and Dcitr05g06500.1.1 with L2FC = −4.26). Two overlaps between transcripts found in the salivary gland and head datasets included RNA-directed DNA polymerase, which is highly down-regulated in *C*Las (+) adults in both datasets, as well as two ATP-synthase transcripts, one up-regulated in salivary glands (ATP synthase γ-chain L2FC = 2.56), one down-regulated in heads (ATP synthase δ-subunit L2FC = −5.33). The full list of statistically significant (*P*adj <0.05) DE (L2FC > |2|) head transcripts can be found in Supplementary Table S6.

### Genome improvement leads to quantifiable differences in RNAseq data interpretation

We hypothesized that, owing to improvements in the v3.0 *D. citri* genome, integrating across different datasets for visualization of tissue-specific responses may have been successful in part due to improved transcript quantification. To test this hypothesis, the midgut dataset was used to compare RNAseq alignment and DE results between the v.1.1 and v.3.0 *D. citri* genome. The two versions of the *D. citri* genome resulted in different interpretations of the midgut transcriptomics results. Genome v3.0 had a 9% higher overall read alignment, as well as 3,000 fewer *D. citri* transcripts found in each biological replicate, on average. After differential expression, fewer statistically significant (*P*adj < 0.05) DE transcripts (L2FC > |0.5|) were matched to genome v3.0 than genome v1.1. Percent alignment of cleaned reads was <100% in all biological replicates for both genomes (Table 3).

**Table 3:** Reanalysis of the midgut transcriptome to quantify the impact of a chromosomal length *Diaphorina citri* genome assembly on transcriptome interpretation, showing comparison of number of raw and trimmed reads from all biological replicates analyzed, as well as percent alignment, number of transcripts, and number of up- and down-regulated transcripts from both the v1.1 and v3.0 genome analysis of *D. citri C*Las (+) midguts

Midgut samples	Raw read cleaning and filtering stats					
	No. raw paired reads (M)	No. reads trimmed^1^	% Aligned^2^		No. transcripts^3^	
			v1.1	v3.0	v1.1	v3.0
*C*Las(−)_1	27.85	273	64.89	73.82	17,170	13,814
*C*Las(−)_2	28.26	234	68.13	77.12	15,284	12,481
*C*Las(−)_3	26.05	246	66.12	74.29	17,566	14,142
*C*Las(+)_1	26.89	76	64.04	73.23	16,339	13,281
*C*Las(+)_2	27.15	210	62.16	71.82	16,834	13,641
*C*Las(+)_3	22.41	117	64.48	74.77	16,476	13,230
	Up-regulated	Down-regulated	Total			
***D. citri* genome v1.1** **^4^**	272 (1.30%)	341 (1.64%)	20,792 (100%)			
***D. citri* genome v3.0** **^4^**	176 (1.38%)	303 (2.38%)	12,704 (100%)			

1 Trimming performed using Trimmomatic to remove adapters and low-quality sequences.

2 Alignment of cleaned reads to each genome performed using Hisat2. Quantities of single- and multi-aligning concordant reads were added together to calculate percent alignment.

3 Transcripts were counted before differential expression and include only named, annotated Dcitr (v3.0) or XM (v1.1) IDs that have 1 or more counts. Not all transcripts are found in all biological replicates and not all are found in both *C*Las (+) and *C*Las (−).

4 Differential expression performed via Ballgown and DESeq2. Transcripts in “TOTAL” column have ≥1 read aligning, while UP- and DOWN-regulated transcripts have adjusted *P* <0.05 and log_2_ fold change >0.5.

**Figure 4:**
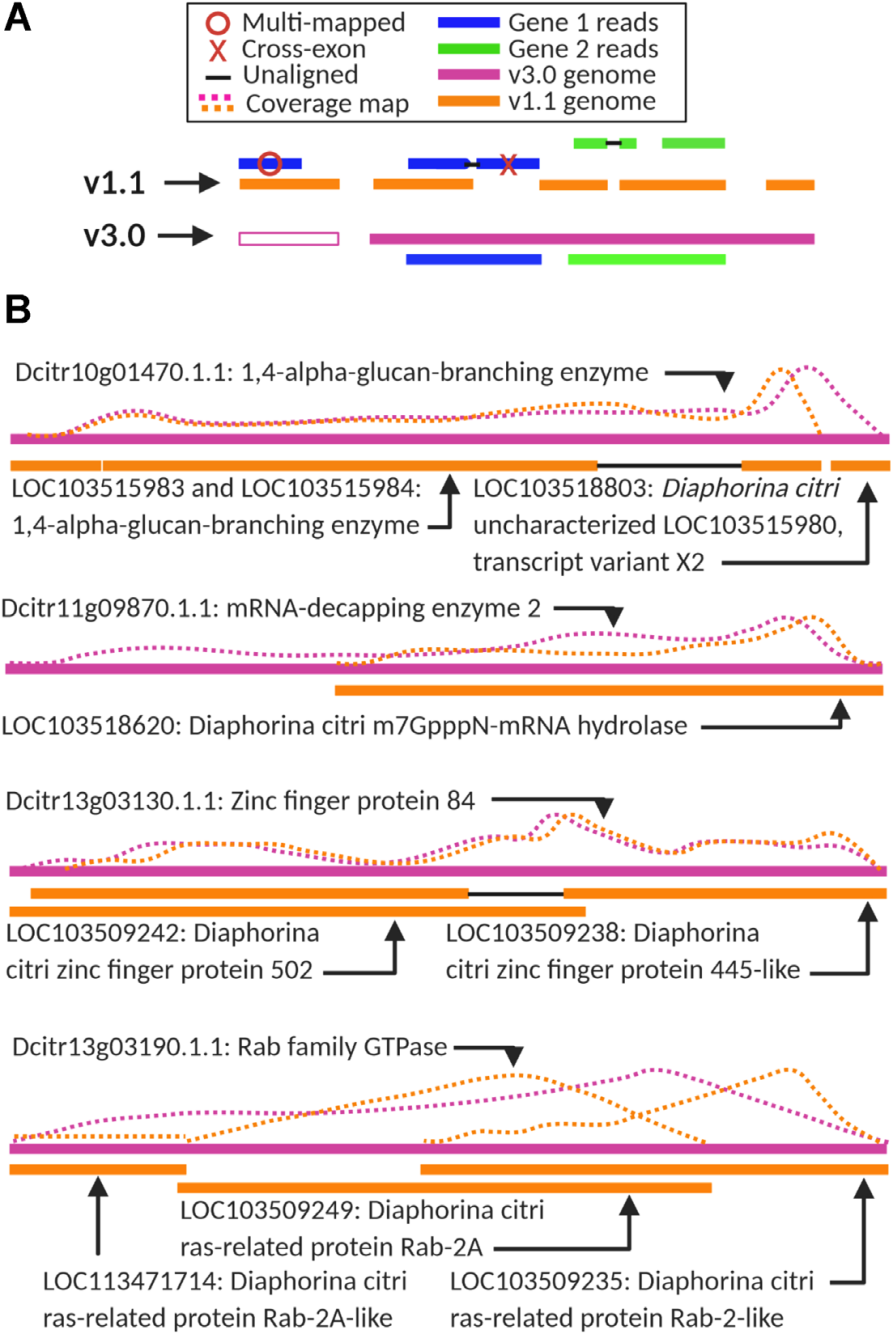
Chromosomal-length *Diaphornia citri* genome assembly improves transcriptome interpretation. (A) Predicted differences between the version Diaci_1.1 and v3.0 *D. citri*genomes. The genes in blue and green together demonstrate multi-mapping, non-alignment due to missing genomic sequence, alignment across a gap in the genome, and the genomic sequence is of such low quality that reads may not match to it perfectly enough to be counted, while the updated genome (pink) fixes or reduces these issues. (B) Four example transcripts showing differences in read alignment as a result of differences between the two genome versions. The pink line represents the newest genome v3.0 while orange represents the older genome, v1.1. Dotted lines demonstrate read alignment to the transcripts in the case of each genome.

Next, we hypothesized several possible ways the genome assembly could affect the interpretation of the transcriptome data (Fig. 4A). The orange genome (representing version 1.1, Fig. 4A) is shown in short fragments with variably sized gaps between the lengths. The reads from Gene 1 (in blue) demonstrate multi-mapping to >1 genomic region, as well as non-alignment due to missing genomic sequence. The reads in green from Gene 2 demonstrate that reads may align across a gap in the genome, and also that a dataset may not have reads to cover all the genome, or, alternatively the genomic sequence is of such low quality that reads may not match to it perfectly enough to be counted. The corrected genome from v3.0 (pink) would be predicted to minimize these spurious mapping occurrences (Fig. 4A, v3.0 genome in pink).

To test whether these differences between genomes have a measurable effect on downstream expression analyses, we selected four random, differentially expressed transcripts (DE in the v3.0 analysis) for an in-depth comparison (Fig. 4B). As predicted, in all four cases, the new gene model was longer and did not contain gaps. In contrast, the associated v1.1 gene models that matched to the full-length transcript were shorter, comprised more fragments, included introns or gaps (Fig. 4B), and were described as “PREDICTED” genes. We matched the read abundance profile over each transcript annotation to demonstrate differences in alignment frequency. The transcript expression associated with each of the v1.1 LOC gene IDs that matched to the sequence from five differentially expressed transcripts from v3.0 (Fig. 4B) were assessed relative to v3.0 transcript expression. In all cases, the differential expression of the v1.1 transcripts in *C*Las-exposed relative to healthy was lower and less significant than the expression of the v3.0 transcripts (Table 4).

**Table 4:** Four statistically significant, differentially expressed *Diaphorina citri*genes from v3.0 midgut alignment were subject to BLAST to find their v1.1 genome equivalent gene IDs, and their total read counts, adjusted *P* values, and log_2_ fold change are compared

v3.0 Gene ID	v3.0 *P*adj^1^	v3.0 log_2_FC^2^	v1.1 Gene ID	v1.1 *P*adj^1^	v1.1 log_2_FC^2^
Dcitr10g01470.1.1	<0.001	−10.69	LOC103515983	0.22	−1.21
			LOC103515984	0.50	−0.92
			LOC103518803*	>0.99	0.34
Dcitr11g09870.1.1	0.01	−0.511	LOC103518620	0.14	1.60
Dcitr13g03130.1.1	0.01	−0.62	LOC103509242	0.87	−0.21
			LOC103509238	0.86	−0.43
Dcitr13g03190.1.1	0.01	0.51	LOC103513428	0.72	0.66
			LOC103509249	0.84	−0.44
			LOC103509235	0.51	0.56
			LOC113471714	0.55	0.53

1 *P* values determined by DESeq2 using Benjamini-Hochberg adjustment of *P* values.

2 The log_2_ fold change (FC) is calculated relative to healthy, so negative values show reduced expression in *C*Las (+) samples, while positive values show increased expression in *C*Las (+) samples.

*Insufficient read alignment counts for statistical analysis of differential expression.

## Discussion

Quantitative measurements of insect vector-borne phytopathogens such as *C*Las pose a major challenge for studying vector-pathogen interactions owing to the underlying biological complexities of the system. The *D. citri* populations used to generate the samples in this study were infected with *C*Las at different percentages, consistent with what has been reported in the literature [9]. Additionally, *C*Las reads were detected at high levels in the salivary gland and head samples, consistent with previous studies of the salivary glands using qPCR analysis [7, 11, 12]. The number of *C*Las reads detected in the salivary gland data suggests that *C*Las is transcriptionally active, indicative of replication, although the lack of detection of similar numbers of *C*Las reads in the bacteriome and midgut does not preclude transcription but suggests that the levels may be below the limit of detection in these samples. Because sample RNA was poly-A enriched using oligos prior to making sequencing libraries, many of the *C*Las transcripts in samples are likely excluded because poly-A tail enrichment biases samples towards eukaryotic mRNAs. Not all of the variation can be attributed to biology. The storage time of these samples at −80 °C (Table 1) was a major driver for variation in the transcriptomes among the salivary gland biological replicates (Supplementary Fig. S2B).

In the salivary glands, the detected *C*Las transcripts had low read counts; most were unannotated, but two transcripts from the *flg* operon and one from the par operon were detected. The *flg* operon is part of the flagellum and is involved in cell motility, cellular processes, chemotaxis, and overall mobility, [43] making it a potentially important gene when *C*Las interacts with its sub-cellular environment in the psyllid. Interestingly, a BLASTx analysis of the coding sequences of both the flgB and flgC transcripts showed homology to multiple *Liberibacter*species (flgC %identity range of 72.93–84.33%, flgB %identity range of 63.08–76.15%). The non-pathogenic *Liberibacter crescens* had the lowest identity (flgC %identity = 67.67%, flgB %identity = 56.92%) relative to the other *Liberibacters*, including “*Ca*. Liberibacter solanacearum,” “*Ca*. Liberibacter americanus,” “*Ca*. Liberibacter africanus,” “*Ca*. Liberibacter europaeus,” and “*Ca*. Liberibacter ctenarytainae.” These results support the hypothesis that the *flg* operon may be active in *Liberibacter* bacteria that are transmitted by psyllids.

The *parB*gene binds DNA and is part of the parABS system, which is known to play a role in bacterial chromosomal partitioning, cell cycle control, and cell division [44], and works by nicking supercoiled plasmid DNA at AT-rich regions and thus can act as a transcriptional regulator. While overall takeaways are limited owing to the low number of reads aligned to this *C*Las gene, finding the *par* operon expressed when *C*Las is in the salivary glands of *D. citri* is consistent with the hypothesis of bacterial genome replication in this organ [11]. Owing to the low number of *C*Las reads found in the other datasets, *parB* was not detected and thus relative expression of this gene could not be compared across tissues.

*D. citri* salivary gland transcripts shed light on mechanisms of transmission and pathogenicity of *C*Las. Ma and colleagues recently published [45] the idea that the pathology of citrus greening disease is due to cell death of phloem cells triggered by reactive oxygen species. The transcripts up-regulated in *C*Las (+) salivary glands suggests that the *D. citri* salivary glands are responding indirectly to the reactive oxygen species environment of the phloem or directly to the infection of this organ by *C*Las.

Excised heads, which contain salivary glands, proved to be a complex and recalcitrant tissue for transcriptome analysis. Excised heads contained multiple organs through which *C*Las-infected phloem or saliva pass, including the esophagus, foregut, mouthparts, and salivary glands. *C*Las has been found in the brain [20], which is also present in head samples. Thus, the head may contain, on average, a greater number of *C*Las bacteria than the other datasets because it contains more organs that *C*Las has been shown to inhabit. However, the head of the psyllid is a highly sclerotized part of the body. Sclerotization may have led to reduced yield when extracting nucleic acids owing to reduced disruption efficiency and blockage of filters. These two possibilities may have led to the low yield—of both raw reads and alignment to the *D. citri* genome in these samples. Additionally, it has been shown that eye fluids of insects can contain PCR inhibitors that may interfere with library amplification and sequencing [46, 47].

The bacteriome is highly specialized and designed to provide a place for replication of obligate bacteria. It is encased in a layer of psyllid cells (bacteriocytes), which could act as a barrier to *C*Las entry. Hosseinzadeh et al. [20] quantified *C*Las titer in multiple organs of *D. citri* and found that bacteriomes contained a very low titer of *C*Las, with only the reproductive organs showing a lower titer. Despite the lack of *C*Las in the bacteriome, it still had marked differences in the transcriptome between *C*Las (+) and *C*Las (−), showing that there are indirect effects of *C*Las infection on psyllid transcription in the bacteriome. Changes in the transporter genes of the bacteriome may be induced indirectly by changes in leaf protein and small-molecule (including amino acid) composition that occur during citrus greening disease [48–50] or directly by the bacterium during psyllid infection and circulative transmission.

An intriguing transcript observed to be differentially expressed in the bacteriome samples is the Dcitr05g01800.11 transcript, which has a log_2_(fold change) of 2.473, with a length 612 nucleotides, annotated as the “PiggyBac transposable element-derived protein 4.” It was significantly differentially expressed in the bacteriome dataset and not the other datasets, suggesting that *C*Las infection of the insect may be inducing transposition in the psyllid bacteriocyte nuclear genome. In the Diaci_v3.0 genome, this transcript is one of ≥11 PiggyBac-related genes found scattered across the genome (see Supplementary Table S9). The PiggyBac (pB) transposon was first discovered 30 years ago in the cabbage looper, and now it is used to transform insects, such as *Drosophila melanogaster*. PiggyBac is unique among transposases because of its specificity and seamless excision [51]. DNA between two sites with the specific sequence “TTAA” can be cleanly excised and the resulting DNA ends can perfectly match again without leaving a genomic footprint or synthesizing any new DNA. Similarly, the excised transposon can be re-integrated at any TTAA site in the genome. Owing to the precision of pB, it is difficult to know exactly where Dcitr05g01800.11 originated—whether from the syncytial cytoplasmic cells, or the outer bacteriocytes. Considering what is known about pB and the bacteriome interactions with endosymbiotic bacteria, Dcitr05g01800.11 is a strong candidate for future studies of the bacteriome and using pB may open pathways for transgenesis in *D. citri*.

The psyllid midgut is the first site of sub-cellular interaction between *C*Las and *D. citri*. A notable observation is that, although there were low levels of *C*Las reads in the midgut, the impact of *C*Las infection on the *D. citri* transcriptome was greatest in the midgut as compared to other tissues, the former of which showed clear separation between *C*Las (+) and *C*Las (−) samples as a result of *C*Las infection. In adult insects, feeding on *C*Las-infected plants has been shown to induce drastic morphological changes to the psyllid nuclear architecture and apoptosis in the midgut epithelial cells [17, 18]. These data suggest that the infected plant sap, and not *C*Las directly, may be playing a role in modulating the midgut transcriptome response. A relatively low replication rate for *C*Las in the midgut vs salivary glands may be an adaptive strategy to switch hosts from plant to insect to evade detection by the psyllid immune system [11, 52] until just prior to transmission to a new host plant.

## Lessons Learned

### Archived transcriptome data are useful and usable together with newly collected data

PCA analysis enabled a global visualization of the variation both within and across the datasets and showed that variance due to time of sample collection was minimal. The bi-axis separation between the four datasets as seen in Fig. 2 can be partially explained by the average amount of *C*Las present (PC1) and by their sequencing (PC2). The head and bacteriome datasets were collected and multiplexed together but sequenced separately from the midgut and salivary gland datasets (which were also sequenced at different times). Head and salivary gland samples produced the highest number of reads aligning to *C*Las in the infected biological replicates, and bacteriome and midgut read counts were relatively low. The clustering of the head and salivary gland data in PC1 was particularly encouraging and showed additional support that transcriptome datasets collected in different experiments can be compared in the same analysis. The head samples were collected from a different cohort of insects than the salivary gland samples, and yet the salivary gland transcriptome was represented in the head transcriptome (Fig. 2).

### Transcript quantification accuracy is improved with full-length genome models

The full-length transcript from the v3.0 analysis was searched against the v1.1 *D. citri* genome using BLAST (see Methods). These analyses clearly show how quantification accuracy is improved with the full-length gene models because all reads matching to a particular transcript are fully accounted for and used for DE analysis. Although each of these transcripts being analyzed is relatively short—comprising ∼600–4,000 nucleotides in length—the difference in read alignment frequency can be in the hundreds. We hypothesized that an improved genome sequence would change how transcriptomics results are interpreted. Analysis of four representative transcripts illustrated the case. In the v1.1 analysis, all ten of these fragmented gene IDs and their associated transcripts would have been disregarded from the DE analysis because their adjusted *P* values did not meet the significance threshold and the differential expression was nearly nonexistent (L2FC < |1|), and/or counts were too low and lacking in the biological replicates to be used. However, according to the v3.0 analysis, each of the four genes and their transcripts should be considered in downstream pathway analyses of effects of *C*Las exposure becausethey satisfied the adjusted *P* value and L2FC cutoffs. Thus, by quantifying how improved genome assemblies can lead to changes in DE, we present evidence to show that long-read sequencing or other genome sequence improvement efforts are foundational for transcriptome-wide expression studies. The improvements in overall read alignment rate of the midgut data to the v3.0 genome compared to the v1.1 genome suggest that, during alignment to the v1.1 genome, thousands of *D. citri* reads were completely left out of the analysis. The lower number of transcripts that matched to genome v3.0 is consistent with the increased scaffold length and gene model improvements.

### Improved genome quality did not determine the proportion of transcripts differentially expressed

The proportion of DE to non-DE transcripts in each dataset may be derived from the biology of the organisms or samples and, in part, to the bioinformatic pipelines but is not due to the improved genome quality. Three studies look at the midgut of *D. citri* using transcriptomics: The analysis by Kruse et al. using v1.1 [16], this study using the Kruse et al. data and the v3.0 genome, and a study by Yu et al. [39] using the v2.0 genome. The source of the midgut RNA is significantly different between the Yu et al. study and the Kruse et al. study. Yu et al. pooled midguts from *D. citri* adults raised on *Murraya exotica*, whereas Kruse et al., and thus, the present study, utilized insects raised on *C. medica*. Yu et al. also reported different *C*Las-infection rates among their individual insects pooled compared to Kruse et al. The relative proportions of transcripts that are up- or down-regulated in each of the three studies are not consistent, nor does the pattern become consistent with improved genome quality. In the studies by Yu et al. and Kruse et al., both groups reported more up-regulated transcripts (499 and 965, respectively) than down-regulated transcripts (279 and 850, respectively), while in the present study, the opposite is true (176 up and 303 down, respectively) (Supplementary Table S3). The discrepancies in the two published midgut transcriptomes underscore the importance of study-level differences as drivers of observed variation. Such differences could be due to the psyllid genotype or the host plant variety on which the insects are reared. For example, host plant switching between the citrus relative *Murraya paniculata* (orange jasmine), a commonly used plant host for rearing *D. citri*, and *Citrus spp*. has been shown to induce changes in the expression of *D. citri* metabolism, immunity, and cytoskeleton proteins [36]. Differences in the computational pipeline may also play a role in reported transcriptome variation. The midgut analysis by Kruse et al. aligned RNA reads to the *D. citri* genome assembly v1.1 using the bioinformatic tools RSEM and bowtie2 for alignment, followed by edgeR and DESeq2 for differential expression calculations. The raw data from Kruse et al. were reanalyzed in the present study using the most recent versions of the bioinformatic tools Hisat2 (genome alignment), Stringtie (transcript assembly), Ballgown, and DESeq2 (DE). These two bioinformatic pipelines differ in their alignment algorithms, statistical methods, and importantly their ability to identify false-positive and false-negative DE transcripts.

### *C*Las-exposed or non-exposed are the most precise descriptions of *D. citri* reared on HLB-positive or uninfected citrus

Detection of *C*Las reads in some tissues and not others leads us to revisit the nomenclature used to describe insects that are sampled from *C*Las-infected plants. Some studies, such as this one, designate insect samples as *C*Las (+) or *C*Las (−), or healthy or infected, referring to the infection status of the tree used to rear the insect. Alternatively, some studies label insects (as opposed to the trees) as *C*Las-exposed or non-exposed, the latter when sampled from healthy, *C*Las-negative trees. The use of “exposed” or “non-exposed” is to account for the finding that not all insects acquire and/or become infected with *C*Las when reared on *C*Las-infected trees [9, 53, 54]. This transcriptomics study suggests that the exposed and non-exposed designations are the most accurate because there is deeper complexity of *C*Las infection status in each insect at the level of the organ. In this study, salivary glands appear to have 10× more *C*Las reads than found in midguts and even more than in bacteriomes, suggesting that salivary glands are truly “infected” and other organs, such as the bacteriome, remain “exposed.”

### Bacterial transcript counts in mRNAseq experiments are not reliable to determine infection of a psyllid tissue with *C*Las

It was difficult to interpret whether psyllid organs were infected on the basis of read count alone when read counts were barely above background, such as in the midguts. Kruse et al. [16] reported that 82% (n = 20, Cq <40) of the *C*Las (+) *D. citri* population that was harvested for their midguts were positive for *C*Las, with a mean qPCR Cq value of 31 across their four *C*Las (+) biological replicates. While 212 is not an especially large number of *C*Las reads post poly-A enrichment, when paired with the qPCR results, midguts, which have been shown to contain a visible slurry of *C*Las cells in previous work using microscopy [16, 18], may be referred to as “infected” by *C*Las, but at a lower level than the salivary glands. However, similar numbers of *C*Las reads were detected in the head samples from insects sampled from healthy (non-exposed) trees as in the midguts, so whether the *C*Las reads in the midguts are meaningful is debatable. Finding a low level of reads aligning to *C*Las in healthy samples is not unexpected and may be due to a few understandable reasons, such as alignment errors, genome annotation errors, or homology of these reads to other psyllid-associated bacteria (the bacterial endosymbionts). *C*Las (−) psyllid colonies and citrus plants are reared in separate but identical environments to *C*Las (+) trees and insects. It is critical that all insect materials be tested regularly and thoroughly for *C*Las using qPCR to rule out the possibility of unintended *C*Las infection of *C*Las (−) samples prior to experimentation.

## Potential Implications

*C*Las is uncultivable, and methods to study *C*Las–*D. citri* interactions are challenging. Genome sequencing is a foundational tool for our exploration of the molecular interactions among *D. citri, C*Las, the bacterial endosymbionts, and the citrus host. Our research showed that improved genome assemblies influence interpretation of transcriptomic data and that investigators have reason to reanalyze their previous *D. citri* transcriptomic data with the new genome release. The more accurate quantification provided by the Diaci_v3.0 genome may reduce the need to validate transcriptomic changes using reverse transcription (RT)-PCR. We urge arthropod genome communities and funding bodies to continue to invest funds in genome improvement projects such as i5k [55] and Ag100Pest [56] and to emphasize reanalyzing previously generated data because it may yield higher confidence results after using an improved-quality genome. Additionally, single-cell RNAseq is the next frontier of understanding insect-pathogen interactions, especially for intracellular symbionts, and provides the highest resolution. Currently, single-cell RNAseq has been done on very few insects, but the list is expanding [57–60].

Still, a major roadblock is the functional annotation of the gene models. While automated pipelines for annotation exist at NCBI and elsewhere [61], these efforts are supplemented by manual annotation efforts [62–66] for *D. citri* and other arthropods [55]. Future work on understanding how the improved genome leads to improved quantification at the proteome level is also needed, and we hope that such studies are inspired by the findings we present here. Our analysis demonstrates that it is possible to analyze new ‘omics data in the context of and alongside historical data in public repositories to maximize the use of existing large-scale dataset resources in discovering new biology. The results underscore the importance of chromosomal-length assemblies of arthropod genomes for accurate interpretation of gene expression.

## Methods

### Experimental design, RNA collection, and sequencing of four *D. citri* RNA datasets

Psyllid colonies and citrus plants used to generate samples for the bacteriome, head, salivary gland, and midgut datasets were continuously maintained by the USDA ARS in Ithaca, NY, and the USDA ARS in Fort Pierce, FL, under the same growth conditions in both locations. These psyllid colonies—including *C*Las (−) and *C*Las (+) *D. citri* adults and nymphs raised on *C. medica* (citron)—were originally started in 1999 from individuals collected from a farm near Fort Pierce, FL, and the *C*Las strain used came with those original individuals. Growth chambers were maintained at 22.8–26.7°C, 70–80% humidity, and a 14 hr light/10 hr dark photoperiod. Citrus plants were grown in greenhouse conditions from seed. *C*Las (+) *C. medica* were inoculated using *C*Las (+) *D. citri*. When insect colonies contained 1–2 week old adults, pools of adult *D. citri* were collected from each colony to create each biological replicate (120 per bacteriome and head replicate [Ithaca colony], 150 per salivary gland replicate [Fort Pierce colony], 250 per midgut replicate (Fort Pierce colony described in [16]). Insects were anesthetized on ice for a few hours prior to and during dissection.

### Bacteriome and head sample preparation in Ithaca, NY

Using a dissecting scope, bacteriomes and heads of adult psyllids were excised into mili-Q (MQ)-water then moved to 2-mL tubes containing 350 µL of buffer RLT from the RNeasy kit (Qiagen, Hilden, Germany) with β-mercaptoethanol and kept on ice during collections. Once the collection of a biological replicate was complete, the tube containing pools of psyllid organs was flash-frozen in liquid nitrogen and stored at −80°C until needed. Total RNA was extracted following the RNeasy extraction protocol (Qiagen, Hilden, Germany), including sample disruption with syringes and DNase treatment to remove DNA contamination.

### Salivary gland and midgut sample preparation in Fort Pierce, FL

Salivary tissues and midguts were preserved in TRIzol (Thermo-Fisher, Waltham, MA). Salivary glands were excised as described by Cicero and Brown [67] in pools of 150 per replicate in TRIzol LS (Thermo-Fisher, Waltham, MA). Samples were kept at −80°C (bioreps 1–3, *C*Las (-/+) were kept one year, while replicate 4, both *C*Las (-/+), was kept for two years) prior to RNA extraction. Total RNA was extracted for both midguts and salivary glands following the standard TRIzol (Thermo-Fisher, Waltham, MA) RNA extraction protocol [68] including light syringe disruption prior to adding ethanol, and DNase treatment to purify total RNA. Total RNA quality was tested using an RNA gel prior to library preparation. Details of midgut sample handling can be found in Kruse et al. [16].

Illumina (San Diego, CA) libraries for all samples were made by Polar Genomics LLC (Ithaca, NY) following the protocol of Zhong et al. [69] and included poly-A tailed mRNA enrichment. Libraries were shipped on dry ice to GENEWIZ (Azenta Life Sciences, Plainfield, NJ), where they were pooled for Illumina (San Diego, CA) paired-end (PE) 150 bp sequencing. Bacteriome, head, and salivary gland samples were sequenced separately from the previously published midgut samples [16]. Raw data have been uploaded to NCBI and are accessible to reviewers via BioProject accession No. PRJNA385527.

### *C*Las titer determination by qPCR

*D. citri* colonies that were exposed to *C*Las and those that were non-exposed were tested for the presence of *C*Las using quantitative PCR (qPCR), yielding a relative (Cq = quantitation cycle) and absolute value of bacterial titer (using a standard dilution curve) by amplification of the 16S rDNA using TaqMan reagents (Thermo-Fisher, Waltham, MA). Individual, whole-body, adult psyllids (n = 50 for the midgut colony, n = 20 for the salivary gland colony, n = 20 for the colony used to collect heads and bacteriomes) were collected from each colony. Total DNA was extracted from individual insects using the DNeasy kit (Qiagen, Hilden, Germany). DNA concentration was estimated using a Nanodrop spectrophotometer (Thermo-Fisher, Waltham, MA). Each sample was standardized to 30 ng/µL and the Cq values from each dataset can be compared directly. The *C*Las probe (5′-FAM-AGACGGGTG/ZEN/AGTAACGCG-3′) sequence and specific forward (5′-TCGAGCGCGTATGCAATACG-3′) and reverse (5′-GCGTTATCCCGTAGAAAAAGGTAG-3′) primers used are as published previously in Kruse et al. [16]. Non-exposed colonies were tested monthly, and *C*Las (+) colonies were tested at the time the insects were collected for dissection. Each qPCR plate contained positive and negative controls as well as a *C*Las 16S rDNA standard curve to allow for both absolute and relative *C*Las titer quantification, and every sample was run in triplicate. For our purposes, only Cq values were required to determine whether individual samples were *C*Las (-/+) and to record the percent infection rate (how many out of 20 were *C*Las [+]) of the colony. A sample was considered *C*Las (+) if the Cq value was <40 (if there is only a single molecule in the reaction, with perfect primer efficiency, 37–40 cycles will be the cycle plateau). The Cq data from all 20 individuals, from all three colonies (bacteriomes and heads were collected from the same individuals and thus the same colony), were compiled and reported in Supplementary Fig. S1. Cq values from the *C*Las non-exposed insects were undetected (40 PCR cycles completed without amplification).

### *In silico* quality control and cleaning of raw data to reduce confounding factors in analysis

Data analysis was conducted on servers hosted by the Computational Biology Center at the Boyce Thompson Institute (Ithaca, NY). Data for all four datasets (bacteriome, head, salivary gland, and midgut) were subjected to identical computational assessments and manipulations to eliminate variability caused by analysis methods. Total raw mRNA reads were first analyzed with FastQC (FastQC, RRID:SCR_014583) [70] to gauge the presence of anomalies and adapters. Illumina (San Diego, CA) universal adapters that were present were removed by first interleaving/merging together forward and reverse reads into one large file. This file was then presented to AdapterRemoval (AdapterRemoval, RRID:SCR_011834) [71] using the Unix commands suggested in the manual for PE read analysis. AdapterRemoval output a file of interleaved PE reads that survived adapter removal. FastQC was run for the second time on this file to confirm adapter removal and check remaining read lengths and total remaining read quantity. This interleaved file was then used as input for SortMeRNA  (SortMeRNA, RRID:SCR_014402) [72], which removes rRNA that survived the poly-A enrichment *in silico*, based on rRNA databases for bacteria, eukaryotes, and archaea provided with the software program. Seed length was adjusted from default 18 down to 14 during rRNA database file indexing to be compatible with the minimum length reads in the current dataset. SortMeRNA supplied two output types: (i) those reads that mapped to rRNA (both forward and reverse reads had to map to be included) and (ii) those where one or both of the PE reads did not map to rRNA, such that the non-rRNA read pool contained some single-strand sequences that aligned to rRNA. Separating out rRNA reduced overexpression and bias of ribosomal gene expression in the datasets without totally removing rRNAs from the analysis. Low-quality sequences (QC <20) were removed with Trimmomatic (Trimmomatic, RRID:SCR_011848) [73]. Paired reads where one or more are shorter than 17 nucleotides were then discarded. FastQC was run for the third time on these files to check their new read length distribution, read number, and overall quality. A shell script was used to unmerge the forward and reverse reads for each sample file (reverse interleaving), creating a set of PE data files containing “cleaned reads” that could be used in the following steps.

### Read alignment to multiple genomes and differential transcript expression for each dataset

All four datasets comprising cleaned, PE mRNA reads were aligned to both the Diaci_v3.0 *D. citri* genome and the “*Candidatus* Liberibacter asiaticus” psy62 genome, which is available on NCBI. The midgut dataset was additionally aligned to the v1.1 *D. citri* genome available on NCBI or CitrusGreening.org. The computational methods closely follow those published by Pertea et al. [74] and include the following: each *D. citri* genome was indexed using HISAT2 (HISAT2, RRID:SCR_015530) (hisat2-build) [75]. Total cleaned reads were aligned to the indexed genome using HISAT2 and standard settings for PE data as described in the HISAT2 manual [75]. Specifically, options added to the base function included index memory mapping (--mm); setting the number of server threads to increase the speed of the alignment (-p); specifying output file names for both concordant alignments and non-concordant alignments (--al-conc and --un-conc, respectively); specifying which of the input files was forward or reverse (specified by “RF” showing −1 was reverse and −2 was forward); and tailoring the output file organization for the possibility of downstream transcript assembly (--dta). Additionally, read alignment statistics were directed into a .stdout file for ease of future reference. Reads that aligned concordantly (collected in the --al-conc output file) were checked with FastQC and used in the next steps. Following alignment, the SAM files were converted to BAM to save space and then sorted by name using SAMtools (SAMTOOLS, RRID:SCR_002105) [76]. Once sorted, reads were bundled into transcripts using StringTie (StringTie, RRID:SCR_016323) [77] based on their alignments and promptly re-aligned to the .GTF/.GFF file specific to each genome, containing information on all known genes for that genome. This process labeled each transcript with a specific Gene_ID, genomic location, and information on introns/exons. Finally, using the number of transcripts that align to each gene, a count matrix was formed using StringTie and Ballgown [74] to allow downstream DE analysis between *C*Las (−) and *C*Las (+) replicates and data visualization. DE was performed in R (v3.3.3) using DESeq2 (DESeq2, RRID:SCR_015687) [78], following standard protocols (DE determined by setting *C*Las [−] as the denominator such that positive L2FC indicates greater expression in *C*Las [+] replicates and negative L2FC indicates reduced expression in *C*Las [+] replicates relative to *C*Las [−]). Because each dataset (except bacteriome and head) was collected and sequenced separately, normalizing the datasets to each other had too many experimental variables that were uncontrollable, so DE analysis for *C*Las (-/+) was performed separately for each dataset. DE results, like those of the qPCR Cq data, could be compared directly for transcripts within a dataset, while transcripts across datasets could be qualified, although no direct or quantitative comparison of expression could be made between datasets as currently analyzed. Reads that aligned to *C*Las in the *C*Las (+) samples were counted and only certain transcripts of interest were analyzed further.

### Statistics and data visualization of results

A variety of statistical methods and data visualization tools were used. A principal component analysis (PCA) of all four datasets combined was performed in R (prcomp and plot) using a large transcript count matrix combining the transcript expression count matrices from the four datasets. The count data were minimally normalized by transcript counts per million, and transcripts not present in both *C*Las (−) and *C*Las (+) replicates were removed. Individual PCA plots were also generated in R (plotPCA and ggplot) to show separation between *C*Las (−) and *C*Las (+) biological replicates, using the DESeq2 rlog-transformed transcript data for each dataset individually. Following PCA analysis, R was used to generate volcano plots of the DE transcripts from each dataset individually, again using the DESeq2 rlog-transformed data. The log2 of the fold change (L2FC) of each DE transcript was plotted against the negative log of the Benjamini-Hochberg adjusted *P*-value for the same transcript, using ggplot.

The comparison of expression results from the midgut dataset when aligned to either v3.0 or v1.1 of the *D. citri* genome was started by choosing four transcripts present and expressed in both analyses. The two genomes presented different gene_IDs and genomic location coordinates, which was problematic for direct comparison of changes in expression or even direct comparison of transcripts. The transcript sequence from Diaci_v3.0 was analyzed using BLASTx (BLASTX, RRID:SCR_001653) against the v1.1 genome to determine which v1.1 transcripts aligned to the v3.0 transcript and whether alignment was partial or full. To demonstrate differences in read distribution between the two genomes for each of the four transcripts and to show differential alignment frequencies, the v3.0 transcript sequences and associated v1.1 transcript sequences were each used as a genome and total cleaned reads were re-aligned to these sequences using HISAT2 to generate the BAM files of read alignments for each transcript. Coverage maps were generated for each transcript using an R script (BEDtools) written by Dave Tang [79]. The general pattern of coverage from these coverage plots was duplicated in cartoon form on top of the respective transcript cartoon to demonstrate the differences in read alignment location and frequency between the two *D. citri* genomes.

Potential secreted effectors were determined from the list of top DE transcripts of the salivary gland dataset by running two programs—SignalP-v5.0 (SignalP, RRID:SCR_015644) [80], which accesses protein sequences for the presence of signal peptides, and Phobius (Phobius, RRID:SCR_015643) [81], which detects both signal peptides and TMHs from a protein sequence. Transcripts that putatively contained signal peptides but not TMHs were considered candidate salivary gland effector proteins.

## Data Availability

Raw data have been uploaded to NCBI via BioProject accession No. PRJNA385527. All supporting data and materials are available in the GigaScience GigaDB database [82].

## Additional Files

Supplementary Figure S1. A histogram of *C*Las Cq values from individuals tested from each colony used to generate RNAseq data.

Supplementary Figure S2. Principal component analysis of all four *Diaphorina citri*transcriptome datasets.

Supplementary Table S1. *C*Las transcripts identified in the *Diaphorina citri*salivary gland transcriptome in three or more biological replicate samples.

Supplementary Table S2. Metadata on RNAseq datasets and alignments.

Supplementary Table S3. All transcripts from the midgut dataset that have differential expression Log2FoldChange>|2| and adjusted *P*-value<0.05. Sorted by Log2FoldChange. Aligned to v3.0 of the *D. citri* genome.

Supplementary Table S4. All transcripts from the salivary gland dataset that have differential expression Log2FoldChange>|2| and adjusted *P*-value<0.05. Sorted by Log2FoldChange. Aligned to v3.0 of the *D. citri* genome.

Supplementary Table S5. All transcripts from the bacteriome dataset that have differential expression Log2FoldChange>|2| and adjusted *P*-value<0.05. Sorted by Log2FoldChange. Aligned to v3.0 of the *D. citri* genome.

Supplementary Table S6. All transcripts from the head dataset that have differential expression Log2FoldChange>|2| and adjusted *P*-value<0.05. Sorted by Log2FoldChange. Aligned to v3.0 of the *D. citri* genome.

Supplementary Table S7. Data used to generate Figure 3, including annotations, *P*-values and Log2FoldChange values for each transcript listed.

Supplementary Table S8. All transcripts from the *Diaphorina citri*salivary gland dataset that have predicted signal sequences. The four in bold text had predicted transmembrane helices.

Supplementary Table S9. All piggyBac-related genes currently annotated in the Diaci_v3.0 genome. The gene identified in our transcript analysis is in bold.

giac035_GIGA-D-21-00314_Original_Submission

giac035_GIGA-D-21-00314_Revision_1

giac035_Response_to_Reviewer_Comments_Original_Submission

giac035_Reviewer_1_Report_Original_Submission Nabil Killiny -- 11/1/2021 Reviewed

giac035_Reviewer_2_Report_Original_Submission Kerry Mauck -- 11/12/2021 Reviewed

giac035_Reviewer_2_Report_Revision_1 Kerry Mauck -- 1/21/2022 Reviewed

## Abbreviations

ARS: Agricultural Research Service; BLAST: Basic Local Alignment Search Tool;*C*Las: *Candidatus* Liberibacter asiaticus; Cq: quantitative cycle; DE: differentially expressed; EF: elongation factor; HLB: huanglongbing; L2FC: log_2_ fold change; mRNA: messenger RNA; NCBI: National Center for Biotechnology Information; PCA: principal component analysis; PE: paired end; rRNA: ribosomal RNA; TMH: transmembrane helices; USDA: United States Department of Agriculture.

## Competing Interests

The authors declare that they have no competing interests.

## Funding

This project was funded by NIFA Predoctoral Fellowship 2021–67011-35143 (M.M.), USDA-NIFA grants 2015–70016-23028 (M.H. and L.M.), 2020–70029-33199 (L.M.), and USDA ARS Project No. 8062–22410-007–00-D (M.H.).

## Authors' Contributions

M.M.: Took part in, or led, all aspects including conceptualization, data curation, formal analysis, funding acquisition, investigation, methodology, validation, visualization, and writing of original draft, as well as review and editing.

S.S.: Funding acquisition, conceptualization, methodology, resources, writing—review and editing.

J.M.C.: Visualization, writing—review and editing, data curation.

M.P.: Methodology, writing—review and editing.

K.M.: Data curation, resources.

L.M.C.: Funding acquisition, project administration, resources, supervision.

W.B.H.: Funding acquisition, project administration, resources, supervision, writing—review and editing.

L.A.M.: Funding acquisition, project administration, methodology, conceptualization resources, supervision, writing—review and editing.

M.H.: Took part in, or led, all aspects including conceptualization, investigation, methodology, project administration, funding acquisition, data analysis, resources, supervision, validation, visualization, writing of original draft and reviews and edits.
