# Supplementary material for: Lessons learned about the biology and genomics of Diaphorina citri infection with “Candidatus Liberibacter asiaticus” by integrating new and archived organ-specific transcriptome data
Source: Gigascience. 2022 Apr 28;11:giac035. doi: 10.1093/gigascience/giac035 (PMC9049105; doi:10.1093/gigascience/giac035)
Supplement: giac035_GIGA-D-21-00314_Original_Submission [file giac035_giga-d-21-00314_original_submission.pdf]

GigaScience

Lessons learned about the biology and genomics of Diaphorina citri infection with “Candidatus Liberibacter asiaticus” by integrating new and archived organ-specific transcriptome data.

--Manuscript Draft--

**Manuscript Number:**	GIGA-D-21-00314
**Full Title:**	Lessons learned about the biology and genomics of Diaphorina citri infection with “Candidatus Liberibacter asiaticus” by integrating new and archived organ-specific transcriptome data.
**Article Type:**	Research
**Funding Information:**	Agricultural Research Service (8062-22410-007-00-) — Dr. Michelle L Heck<br>National Institute of Food and Agriculture (2015-70016-23028) — Dr. Michelle L Heck<br>National Institute of Food and Agriculture (2020-70029-33199) — Dr. Lukas A. Mueller<br>National Institute of Food and Agriculture (2021-67011-35143) — Miss Marina Mann

Abstract:

Background

Huanglongbing (HLB), a devastating disease of citrus, is caused by the obligate, intracellular bacterium “ Candidatus Liberibacter asiaticus” (C Las). C Las is transmitted by Diaphorina citri , the Asian citrus psyllid. Development of transmission-blocking strategies to manage HLB relies on knowledge of C Las- D. citri interactions at the molecular level. Prior transcriptome analyses of D. citri point to changes in psyllid biology due to C Las-infection but have been hampered by incomplete versions of the D. citri genome, proper host plant controls, and/or a lack of a uniform data analysis approach. Therefore, using the scaffold of the newest chromosomal length D. citri genome assembly, Diaci_v3.0, we computationally identified differentially expressed genes in both C Las (+) and C Las (-) D. citri . In this work, we present lessons learned from a quantitative transcriptome analysis of excised heads, salivary glands, midguts and bacteriomes from C Las (+) and C Las (-) insects.

Results

Each organ had unique transcriptome profiles and responses to C Las infection. Though most psyllids were infected with C Las, C Las-derived transcripts were not detected in all organs. By analyzing the midgut dataset using both the Diaci_v1.1 and v3.0 D. citri genomes, we showed that improved genome assembly led to significant and quantifiable differences in RNAseq data interpretation.

Conclusions

Our results support the hypothesis that future transcriptome studies on circulative, vector-borne pathogens should be conducted at the tissue specific level using complete, chromosomal-length genome assemblies for the most accurate understanding of pathogen-induced changes in vector gene expression.

**Corresponding Author:**	Michelle L Heck, Ph.D.<br><br>UNITED STATES
**Corresponding Author Secondary Information:**	
**Corresponding Author's Institution:**	
**Corresponding Author's Secondary Institution:**	

Powered by Editorial Manager® and ProduXion Manager® from Aries Systems Corporation

**First Author:**	Marina Mann
**First Author Secondary Information:**	
**Order of Authors:**	Marina Mann
	Surya Saha, Ph.D.
	Joseph M. Cicero, Ph.D.
	Marco Pitino
	Kathy Moulton
	Wayne B. Hunter, Ph.D.
	Lilianna Cano
	Lukas A. Mueller, Ph.D.
	Michelle L Heck, Ph.D.
**Order of Authors Secondary Information:**	

**Additional Information:**	
**Question**	**Response**
Are you submitting this manuscript to a special series or article collection?	No
**Experimental design and statistics**<br><br>Full details of the experimental design and statistical methods used should be given in the Methods section, as detailed in our Minimum Standards Reporting Checklist. Information essential to interpreting the data presented should be made available in the figure legends.<br><br>Have you included all the information requested in your manuscript?	Yes
**Resources**<br><br>A description of all resources used, including antibodies, cell lines, animals and software tools, with enough information to allow them to be uniquely identified, should be included in the Methods section. Authors are strongly encouraged to cite Research Resource Identifiers (RRIDs) for antibodies, model organisms and tools, where possible.<br><br>Have you included the information requested as detailed in our Minimum	Yes

Standards Reporting Checklist?	
**Availability of data and materials**<br><br>All datasets and code on which the conclusions of the paper rely must be either included in your submission or deposited in publicly available repositories (where available and ethically appropriate), referencing such data using a unique identifier in the references and in the "Availability of Data and Materials" section of your manuscript.<br><br>Have you have met the above requirement as detailed in our Minimum Standards Reporting Checklist?	Yes

Powered by Editorial Manager® and ProduXion Manager® from Aries Systems Corporation

Click here to access/download;Manuscript;10-1 Submission.pdf

Lessons learned about the biology and genomics of *Diaphorina citri* infection with *"Candidatus* Liberibacter asiaticus" by integrating new and archived organ-specific transcriptome data.

Marina Mann[1], Surya Saha[2,3], Joseph M. Cicero[4], Marco Pitino[5], Kathy Moulton[6], Wayne B. Hunter[6], Lilianna Cano[7], Lukas A. Mueller[2], Michelle Heck[1,8*]

[1]Plant Pathology and Plant-Microbe Biology Section, School of Integrative Plant Science, Cornell University, Ithaca, NY 14853, USA

[2]Boyce Thompson Institute, Ithaca, NY 14853, USA

[3]School of Animal and Comparative Biomedical Sciences, 1117 E. Lowell Street, Tucson AZ 85721 USA

[4]School of Plant Sciences, University of Arizona, Tucson, AZ 85721 USA

[5] AgroSource, Inc. Juniper, FL 33469

[6]U.S. Horticultural Research Laboratory, Unit of Subtropical Insects and Horticulture, USDA Agricultural Research Service, Fort Pierce, FL 34945, USA

[7]Indian River Research and Education Center, Fort Pierce, FL 34945 USA

[8]Emerging Pests and Pathogens Research Unit, Robert W. Holley Center, United States Department of Agriculture Agricultural Research Service, Ithaca, NY 14853, USA

[*]To whom correspondence should be addressed:

Michelle Heck, michelle.cilia@usda.gov

Abstract:

Background

Huanglongbing (HLB), a devastating disease of citrus, is caused by the obligate, intracellular bacterium "*Candidatus* Liberibacter asiaticus" (*C*Las). *C*Las is transmitted by *Diaphorina citri*, the Asian citrus psyllid. Development of transmission-blocking strategies to manage HLB relies on knowledge of *C*Las-*D. citri* interactions at the molecular level. Prior transcriptome analyses of *D. citri* point to changes in psyllid biology due to *C*Las-infection but have been hampered by incomplete versions of the *D. citri* genome, proper host plant controls, and/or a lack of a uniform data analysis approach. Therefore, using the scaffold of the newest chromosomal length *D. citri* genome assembly, Diaci_v3.0, we computationally identified differentially expressed genes in both *C*Las (+) and *C*Las (-) *D. citri*. In this work, we present lessons learned from a quantitative transcriptome analysis of excised heads, salivary glands, midguts and bacteriomes from *C*Las (+) and *C*Las (-) insects.

Results

Each organ had unique transcriptome profiles and responses to *C*Las infection. Though most psyllids were infected with *C*Las, *C*Las-derived transcripts were not detected in all organs. By analyzing the midgut dataset using both the Diaci_v1.1 and v3.0 *D. citri* genomes, we showed that improved genome assembly led to significant and quantifiable differences in RNAseq data interpretation.

Conclusions

Our results support the hypothesis that future transcriptome studies on circulative, vector-borne pathogens should be conducted at the tissue specific level using complete, chromosomal-length

genome assemblies for the most accurate understanding of pathogen-induced changes in vector gene expression.

Keywords

Diaphorina citri, Huanglongbing, *Candidatus* Liberibacter asiaticus, transcriptomics, citrus, vector-pathogen interactions

Background

Huanglongbing (HLB), also known as citrus greening, is the most serious disease of citrus (reviewed in [1-3]). HLB symptoms include leaves with blotchy, chlorotic mottling, stunting, loss of root biomass, premature fruit drop, uneven fruit development, and ultimately tree death. In the USA and Asia, HLB is associated with plant vascular tissue infection by the gram-negative, uncultivable Alphaproteobacteria "*Candidatus* Liberibacter asiaticus" (*C*Las). The Asian citrus psyllid *Diaphorina citri* Kuwayama (Hemiptera: Liviidae) is the vector of *C*Las. HLB has decimated a multi-billion dollar industry in Florida and is threatening the industries in Texas and California [4].

Evidence thus far on *C*Las transmission by *D. citri* is consistent with a circulative, propagative transmission mode that is inextricably linked to the insect's development and intracellular environment surrounding *C*Las bacteria (Figure 1) [5]. During the circulative propagative transmission cycle of *C*Las, *D. citri* acquire *C*Las from an infected citrus host during phloem ingestion as early as the 2nd nymphal instar [6] but in increasing amounts during the 4th and 5th instars of the nymphal stage [7]. The bacteria remain associated with the insect during molting [7, 8]. *C*Las circulates throughout the body of *D. citri* until it reaches the salivary gland

tissues, where it replicates to high levels in the adults [9-12]. The titer of *C*Las increases, presumably in the salivary gland tissue, over approximately 1-2 weeks [7]. The infected adults are then competent vectors capable of tree-to-tree spread of *C*Las. *C*Las is detectable in the insect's alimentary canal, especially the midgut [9, 10, 13]. The bacteria also systemically infect the psyllid during propagative transmission, including the hemolymph, salivary glands, muscle, fat body and reproductive organs (reviewed in [3]). Specific cellular receptors in these different *D. citri* tissues are not known. In adults, *C*Las forms a biofilm along the midgut and induces apoptosis of midgut epithelial cells [14], a process which is not observed in nymph midguts [15]. In the midgut, the bacterium is hypothesized to be associated with the endoplasmic reticulum based on microscopic observations [16]. The movement and infection of *C*Las in the vector tissues predicts extensive vector-pathogen interactions at the molecular level. This paper will use the *C*Las (+) and *C*Las (-) designation to refer to the different sample groups, where the *C*Las (+) insects were reared on *C*Las-infected trees and the *C*Las (-) insects were reared on healthy citrus which also tested negative for *C*Las by quantitative PCR (qPCR).

D. citri harbors three bacterial symbionts, "*Candidatus* Profftella armatura," "*Candidatus* Carsonella ruddii," and *Wolbachia-Diaphorina* (wDi) [17-23] which reside in a specialized organ referred to as the bacteriome. The bacteriome is comprised of bacteriocytes – psyllid cells densely packed with the endosymbiotic bacteria. The bacteriome of *D. citri* has a precise and elegant cellular organization that has been described using fluorescence microscopy [17, 21]. *Carsonella* resides in the outer bacteriocytes and *Profftella* resides in the internal syncytial cytoplasm of the bacteriome. The function of these beneficial bacterial symbionts in the biology of *D. citri* is inferred from bacterial genome sequencing, proteomics and metabolite data.

Rapid advances in genome sequencing technologies have paved the way to a deeper understanding of vector biology over the past decade, including in the analysis of the *D. citri* genome sequence [19, 24-27]. The short read-based assembly, Diaci_v1.1 [26, 28], has been foundational to the vast majority of published research on *D. citri* to date, including the newest chromosomal length reference genome [27], which is expected to lend more reliability and contain more cohesive, full-length annotated gene models. Numerous studies have used these valuable *D. citri* genome sequencing resources to investigate interactions between *D. citri* and *C*Las and *D. citri* biology at the transcriptome and proteome levels [13, 29-32]. Wu and colleagues [33] published a thorough RNAseq experiment including an analysis of organs, sexes, and life stages of *D. citri*. Their analysis focused on potential insecticide detoxification genes from *C*Las (-) insects raised on a close relative of citrus known to be resistant to systemic infection by *C*Las, *Murraya exotica*, but did not address the impact of *C*Las infection in these organs. A year later, the same group published a paired transcriptome-proteome paper focusing on *C*Las (-) *D. citri* salivary glands and associated salivary secretions [34]. They focused on identifying bioactive molecules from the saliva and salivary gland 'omics analysis and discussed proteins that were found uniquely in the salivary glands from *D. citri* reared on healthy plants.

Tissue-specific omics analyses enables a molecular snapshot of *C*Las-*D. citri* interactions within specific tissues known to be colonized by *C*Las in the insect. Studies have revealed stark differences in patterns of expression when comparing tissue-specific responses to whole body responses [13, 31]. However, earlier studies were limited in the interpretation of the data because of the incomplete nature of the *D. citri* genome that were used as a backbone for the quantitative analysis and the application of different computational workflows to identify differentially expressed genes. Kruse et al. (2016) did a thorough analysis and discussed the midgut

transcriptomics responses to *C*Las using four biological replicates of pools of hundreds of midguts and performed dual differential expression analysis using two types of computational biology tools, edgeR and DESeq2, to reduce the false discovery rates [13]. However, the results were dependent on paired proteomics and transcriptomics that were both aligned to the relatively low quality and incomplete v1.1 *D. citri* genome, the assembly available at the time. Yu and colleagues [35] built on the Kruse et al. study [13] using the *D. citri* v2.0 genome, which also lacked the Hi-C scaffolding included in the newest v3.0 genome. Despite the limitations of the genome sequences used for the analyses of these transcriptomes, the results clearly showed that *C*Las has different effects on metabolic pathways expressed within different tissues of *D. citri*. To understand the nature of the *C*Las-*D. citri* relationship at the molecular level, a holistic approach which both integrates the responses across different tissues involved in the circulative transmission pathway and quantifies the impact of *C*Las infection on the transcriptional regulation within specific tissues is necessary.

In this work, we report the first comparative transcriptome analysis of *C*Las (-) to *C*Las (+) psyllid bacteriomes, salivary glands and heads. Using the newest *D. citri* genome assembly (v3.0), which includes chromosomal length scaffolds [27], we analyzed these new data together with previously published *C*Las (+) and *C*Las (-) midgut data [13]. This study advances our understanding of *D. citri*-*C*Las interactions because it integrates an analysis of new transcriptome data with previously published transcriptome data to show the impact of *C*Las on the transcriptional landscape of *D. citri* organs involved in the circulative, propagative transmission using the latest genomic resources. The lessons learned from and difficulties of comparing the four datasets – three of which were collected from separate insect colonies, at different times, sequenced separately, stored in freezers for different lengths of time, and contain

variable amounts of *C*Las in each tissue type – should be acknowledged. This study does not purport to have controlled for all differences found between these datasets, but we do attempt to carefully explain results within the bounds of our controls and include caveats for the confounding effects and the lessons learned from this analysis.

Figure 1. Schematic of *Diaphorina citri* on a citrus leaf, showing the anatomical location and physical details of four parts that were extracted from adult *D. citri* to create four datasets (gut - green, bacteriome - yellow, salivary gland - blue, head – dark purple). The circulative transmission of "*Candidatus* Liberibacter asiaticus" (*C*Las represented by small grey lines) is represented as *C*Las travels from leaf veins through the gut, crossing the midgut epithelial cell layer to circulate in the body of *D. citri. C*Las enters the salivary gland where it is known by contributory effects from acquisition by late instar nymphs, to replicate to high levels, at which point it can be inoculated into the phloem while adult *D. citri* feed (see 3D imaging and digital video by Alba-Tercedor et al. (2021) for more details [36]). *C*Las (-) adults transmit *C*Las inefficiently if the bacteria are acquired during the adult stage.

Data Description

Experimental design, RNA collection, and sequencing of four *D. citri* RNA datasets.

Psyllid colonies and citrus plants used to generate samples for the bacteriome, head and midgut datasets were continuously maintained by the USDA Agricultural Research Service (ARS) in Ithaca NY and the USDA ARS in Fort Pierce, FL under the same growth conditions. These psyllid colonies – including *C*Las (-) and *C*Las (+) *D. citri* adults and nymphs raised on

Citrus medica (Citron) – were originally started in 1999 from individuals collected from a farm near Fort Pierce, Florida and the *C*Las strain used came with those original individuals. Growth chambers were maintained at 22.8ºC-26.7ºC, 70-80% humidity and a 14h light/10h dark photoperiod. Citrus plants were grown in greenhouse conditions from seed. *C*Las (+) *C. medica* were inoculated using *C*Las (+) *D. citri*. When insect colonies contained 1-2 week old adults, pools of adult *D. citri* were collected from each colony to create each biological replicate (120 per bacteriome and head replicate (Ithaca colony), 150 per salivary gland replicate (Fort Pierce colony), 250 per midgut replicate (Fort Pierce colony described in [13]). Insects were anesthetized on ice for a few hours prior to and during dissection.

Bacteriome and head samples.

Using a dissecting scope, bacteriomes and heads of adult psyllids were excised into mili-Q (MQ)-water then moved to 2ml tubes containing 350ul of buffer RLT (Qiagen RNeasy kit) with beta-mercaptoethanol and kept on ice during collections. Once the collection of a biological replicate was complete, the tube containing pools of psyllid organs was flash frozen in liquid nitrogen and stored in -80ºC until needed. Total RNA was extracted following the Qiagen RNeasy extraction protocol, including sample disruption with syringes and DNase treatment to remove DNA contamination.

Salivary glands and midguts.

Salivary tissues and midguts were preserved in TriZol. Salivary glands were excised as described by Cicero and Brown [37] in pools of 300 per replicate in TRIzol LS (ThermoFisher). Samples were kept at -80 º C (bioreps 1-3, *C*Las (-/+) were kept 1 year, while replicate 4, both *C*Las (-/+), was kept for 2 years) prior to RNA extraction. Total RNA was extracted for both midguts and salivary glands following the standard TRIzol RNA extraction protocol [38]

including light syringe disruption prior to adding ethanol, and DNase treatment to purify total RNA. Total RNA quality was tested using an RNA gel prior to library preparation. Details of midgut sample handling can be found in Kruse et al. [13].

Illumina libraries for all samples were made by Polar Genomics LLC following the protocol of Zhong et al., [39] and included poly-A tailed mRNA enrichment. Libraries were shipped on dry ice to GENEWIZ where they were pooled for Illumina paired-end 150bp sequencing. Bacteriome, head and salivary gland samples were sequenced separately from the previously published midgut samples. Raw data has been uploaded to NCBI and is accessible to reviewers via BioProject accession # PRJNA385527, submission ID SUB10382129 and will be made available to the public upon publication.

Analyses

Though most psyllids were infected with *C*Las, *C*Las-derived RNAseq reads were not detected in all organs.

Using quantitative PCR (pPCR) analysis of whole insects, we determined the *C*Las infection rate of the *D. citri* populations used for dissections. Across all sample types, the percent infection rate ranged between 73-85%. Cq values lower than 40 were counted as *C*Las (+) (Table 1). In addition to a population-level assessment of *C*Las infection, we quantified *C*Las-mapped reads found within each sample after sequencing (Table 1 and Figure S1). Read counts mapping to the *C*Las-psy62 genome (genome produced from a single psyllid in FL [40]) were detected above background in *C*Las (+) salivary gland and head samples (an average of 1965 and 2681 reads, respectively), suggesting some AT-rich sequences were captured during poly-A enrichment. Upon closer analysis of the *C*Las-aligning reads from the salivary glands,

when at least three biological replicates had a transcript with at least one read, 50 unique *C*Las mRNA transcripts were represented, with an additional six rRNA transcripts (three of each 16S and 23S transcripts), for a total of 56 *C*Las-psy62 transcripts identified. The majority of *C*Las reads from the salivary glands aligned to the top 10 transcripts, where the total number of reads across all biological replicates of each transcript ranged from 80 to 290. Of these top 10, three were listed as "protein coding" and annotated as figB, figC, and parB, while the rest were unlabeled/unknown (Table S1).

Table 1. Percent infection by *C*Las in different *D. citri* tissues as measured by qPCR, and the average number of RNAseq reads that aligned to the *C*Las genome (psy62) from each dataset.

**Dataset**		**Avg # *C*Las reads**	**% Infection[+]**
**Midgut[1]**	***C*Las (-)**	1*	0
	***C*Las (+)**	212	82%
**Salivary gland[2]**	***C*Las (-)**	0.5	0
	***C*Las (+)**	1965.5	73%
**Bacteriome[3]**	***C*Las (-)**	0.8	0
	***C*Las (+)**	3.4	85%
**Head[3]**	***C*Las (-)**	174.6	0
	***C*Las (+)**	2681.8	85%

[1] qPCR Cq data from Kruse et al. 2017, reads aligning to *C*Las are from our own alignments.
[2]Salivary glands from a colony with a high (>90%) infection rate.
[3]Bacteriomes and heads were taken from the same insects, and whole insects were used for qPCR of *C*Las titer, so the average Cq value is the same for both datasets.
*Low read counts may represent sequences from contaminating *C*Las sequences remaining within the *D. citri* genome (which need to be removed), or representative of sequences transferred to *D. citri*, or found in common in other bacterial symbionts present.
[+]Cq values of 40 translate to 0 titer of the target bacterium. Cq values are calculated using 20-30 whole body individuals from each parent colony of each dataset. All Cq<40 are counted for percent infection.

Global assessment of four transcriptomics datasets clarifies the *D. citri* organ-specific response to *C*Las.

Across all four datasets, we obtained an average of 27.23 million high-quality reads, (midguts: 26.43M, salivary glands: 44.98M, bacteriomes: 22.11M, heads: 15.40M), and 71.3% of the reads aligned concordantly to the v3.0 *D. citri* genome on average (average concordant alignment in midguts: 74.17%, salivary glands: 73.51%, bacteriomes: 81.12%, heads: 56.43). The head dataset proved to be more variable as compared to the other datasets, recording the least number of raw reads and the lowest average percent alignment. In contrast, the highest percent alignment to the *D. citri* genome was recorded by the bacteriome dataset, samples of which were collected from the same individual insects as the head dataset (Table S2).

A principal components analysis (PCA) to examine the sources of variation among the four *D. citri* dataset expression profiles was performed, where each dataset includes both *C*Las (+) and *C*Las (-) biological replicates. Each organ separated from the other organs in PCA space, showing that each organ has a unique transcriptome profile. The largest source of variation (PC1 = 21%) was explained by differences in the transcriptome profiles of the midgut and bacteriome as compared to the salivary gland and head (Figure 2). The second largest source of variation between the four datasets (PC2 = 18%) was explained by differences between the midgut and the bacteriome datasets, with a smaller amount of variation between those samples and the head and salivary gland datasets along the same principal component. Importantly, biological replicates of each dataset clustered together and separately from the others (Figure S2), supporting the hypothesis that each organ has a unique transcriptomic signature independent of *C*Las infection. A closer examination of the four clusters showed that the salivary gland, bacteriome and head datasets did not differentiate between *C*Las (-) and *C*Las (+) biological replicates (Figure S2B, S2C, S2D), while midguts (Figure S2A) showed a clear separation along PC1 between *C*Las (-) and *C*Las (+) biological replicates.

PCA plots of each organ dataset comparing *C*Las (+) to *C*Las (-) revealed other sources of variation (Figure S2). The variance described by PC1 of the salivary gland dataset (44.1%, Figure S2B) was explained by two samples which were kept in the -80C freezer and then sequenced a year after the other six samples, while PC2 (19%, Figure S2B) represented the effect of *C*Las infection which is not distinct, except for the two outlier samples. The bacteriome dataset (Figure S2C) showed some separation between *C*Las (+) and *C*Las (-) biological replicates (PC2=15.9%) but the majority of variation was due to variance among individual biological replicates (PC1=16.7%). The head dataset (Figure S2D) showed similar variation across all samples as the bacteriome dataset. This variation explained both the first and second major sources of variance (PC1=39.7%, PC2=27.3%) with no obvious distinctions between *C*Las (+) and *C*Las (-) biological replicates.

Figure 2: Principal components analysis (PCA) of four *D. citri* mRNAseq datasets (head, midgut, salivary gland and bacteriome), each composed of *C*Las (+) and *C*Las (-) biological replicates, showing the two main sources of variation among them. PC1 (21%) separates samples containing salivary tissues (head and salivary gland samples) from the other datasets, while PC2 (18%) distinguishes the bacteriome and head datasets (which were collected in parallel from the same individual insects), from the salivary gland and midgut datasets (which were collected independently). Raw read counts were processed by DESeq2 using the Benjamini-Hochberg normalization method before generating the principal components plot.

Gene expression signatures in response to *C*Las infection are tissue-specific in *D. citri*.

Differentially expressed transcripts expressed in *C*Las (+) or *C*Las (-) replicates in addition to transcripts that were present in but differentially expressed between *C*Las (+) and *C*Las (-) biological replicates using the maximum adjusted p-value of 0.05 and a Log2FoldChange (L2FC) of >|2| were used for downstream analyses. This strict quality and DE threshold limited the number of final transcripts to a small number (midgut=196, salivary gland=105, bacteriome=113, head=10) (see Tables S3, Table S4, Table S5, and Table S6 for the list of transcripts). A skew towards up-regulated transcripts in *C*Las (+) biological replicates was detected in all organs (salivary gland: up-regulated=91, down-regulated=14; midgut: up-regulated=129, down-regulated=67; bacteriome: up-regulated=70, down-regulated=43; head: up-regulated=6, down-regulated=4).

Four major groups of transcripts were chosen based on their strong representation among the top differentially expressed gene (DEG) lists from the salivary gland, bacteriome and midgut datasets (Figure 3, Table S7) for a more detailed analysis to highlight the tissue-specific patterns of transcriptional activation in response to *C*Las. The four groups include ribosomal transcripts, immunity-related transcripts, endocytosis-related transcripts and ubiquination-related transcripts. Each dataset varies in its strength of response (as measured by L2FC and the relative number of transcripts found in each of the four categories). Ubiquitination genes are highly upregulated in the salivary gland dataset (Figure 3A, green bars). Endocytosis genes are highly upregulated in all tissue datasets (Figure 3B). Immunity genes are upregulated in the salivary glands and midguts but not the bacteriomes (Figure 3C, green and orange bars vs. yellow bars). Different ribosomal genes are upregulated in *C*Las (+) samples in all three datasets (Figure 3D), despite ribosomal transcript depletion *in silico*.

Figure 3: Transcripts have unique expressions across different organs of *D. citri.* The top differentially expressed (DE) transcripts from each dataset (bacteriome, midgut and salivary gland) are sorted by major functional groups including ubiquination, endocytosis, immunity and ribosomal-related transcripts. Not all transcripts are statistically DE, one transcript may be DE in one dataset, but not the others. See Table S7 for p-values.

In addition to the major patterns (Figure 3), selected transcripts of interest also showed notable changes in expression in the datasets consistent with the functions of these tissues in *D. citri* physiology that may give insight into how *C*Las is interacting with these specific tissues at the molecular level. These changes are discussed here.

Midgut: The top differentially expressed transcripts from the midgut dataset were manually sorted into five additional functional categories including biosynthesis and catabolism (n=55, 40 up-regulated in *C*Las (+), 15 down-regulated), cell structure and signaling (n=66, 38 up, 28 down), stress (n=10, 6 up, 4 down), transport (n=28, 19 up, 9 down), and unknown (n=37, 26 up, 11 down). The full list can be found in Table S3. Differentially expressed transcripts in the stress category include heat shock and cold shock protein genes, thioredoxin, and E3 ubiquitin ligase. Three heat shock proteins (70-A1, 70-B, 70) are up-regulated with exposure to *C*Las, while the cold shock protein is down regulated. An E3 ubiquitin ligase, a type IV collagenase and tumor protein p53 are also up-regulated. A thioredoxin transcript and a HSP20-like chaperone transcript are down-regulated with exposure to *C*Las. Transport-related transcripts that are up-regulated with *C*Las-infection include two odorant-binding protein transcripts, membrane-associated ion transporters (aquaporin, major facilitator, protein-coupled AA-transporter, efflux system protein transcript, phosphate transporter, potassium channel

protein transcript, and general secretion pathway transcripts), a vacuolar-sorting protein transcript, and an intraflagellar transport particle protein transcript, among others. Down-regulated transcripts include syntaxin, ubiquinol cytochrome-c, membrane-associated proteins and transporters, and nuclear transport factor 2.

***Salivary gland*:** The full list of statistically significant (padj<0.05) salivary gland differentially expressed (L2FC>|2|) transcripts can be found in Table S4. Transcripts for 40S and 60S subunits of the eukaryotic ribosome are highly up-regulated (40S S15a L2FC=10.12, 40S S28 L2FC=10.52, 60S L2FC=5.53), as well as six transcripts involved with transport which are all up-regulated (ABC transporter C family L2FC=5.95, alpha-tocopherol transfer protein L2FC=8.04, gamma-glutamylcyclotransferase L2FC=8.15, geranylgeranyl transferase L2FC=6.22, MFS-type transporter L2FC=4.03, and phosphate acetyltransferase L2FC=9.30). Additionally, four elongation factor (EF) transcripts are highly up-regulated (EF-1b, EF-2, EF-4 and a Calcium-binding EF hand), consistent with increased ribosomal activity. While ubiquination-related transcripts are present in every dataset, in the salivary gland dataset two transcripts are highly up-regulated including a ubiquitin conjugating enzyme (L2FC=3.70) and ubiquitin-ligase E3 (L2FC=4.69). [35].

Since the salivary gland is known as a secretory organ, the most abundant transcripts were checked for both the presence of transmembrane helices (TMHs) and for signal sequences, the first step towards identifying secreted effectors that would modulate interactions between *D. citri* and the citrus host plant differentially during *C*Las transmission. A total of 12 candidate *D. citri* secreted effectors were found: five lack annotation or are otherwise *D. citri*-specific, and four were predicted to contain a TMH. Of the eight candidate salivary gland effector transcripts without TMHs, seven are highly up-regulated in *C*Las (+) adult *D. citri*, while one of the

unknown transcripts is highly down-regulated in *C*Las (+) adult salivary glands. (Table S8). A recent paper by Wu et al [34] looked closely at salivary proteins and transcripts from CLas (-) *D. citri*, and of the eight possible effectors identified by this study, only the serine proteases were found in common, suggesting that the diversity of secreted effectors is vast, context dependent and requires additional study.

Bacteriome: The bacteriome, home to the psyllid's bacterial endosymbionts, responded to *C*Las infection at the transcriptional level. A key group of bacteriome transcripts involved in communication between *D. citri* and its obligate endosymbionts in the presence of *C*Las are the transporters, methyltransferases, acetyltransferases and the PiggyBac transposable elements, which together are represented in the top DE transcript list by 10 different transcripts. Three methyltransferases are all highly up-regulated in the *C*Las (+) adult bacteriome (methyltransferase family protein L2FC=7.48, phthiotriol dimycocerosates methyltransferase L2FC=5.48, and protein arginine N-methyltransferase L2FC=2.10) and one acetyltransferase is down-regulated (histone acetyltransferase catalytic subunit L2FC= -2.17). Five transcripts are annotated as "transporters" including three that are up-regulated in *C*Las (+), (cation-chloride cotransporter L2FC=3.07, cationic amino acid transporter L2FC=8.43, major facilitator transporter L2FC=5.59) and two that are down-regulated in *C*Las (+), (ABC transporter G family protein L2FC= -2.13 and organic solute transporter ostalpha protein L2FC= -2.31). Three ribosomal-related transcripts are up-regulated in the *C*Las (+) adult *D. citri* bacteriome (60S L26 with L2FC=4.03, 60S L37a with L2FC=3.25, and ribosomal protein L23 with L2FC=2.93). The full list of statistically significant (padj<0.05) bacteriome differentially expressed (L2FC>|2|) transcripts can be found in Table S5.

Head: The head dataset had relatively few reads sequenced and likewise, very few transcripts were statistically significantly DE. Of the 10 with padj<0.05 and L2FC>|2|, half (n=5) were associated with cell structure and signaling, including a vigilin gene with L2FC=-4.09, a DNA-polymerase gene with L2FC=-3.42, a Rho-GTPase with L2FC=5.28, a neuromodulin gene with L2FC=5.27, and an insulin-like growth factor with L2FC=5.63. One transcript was associated with activation of autophagy, Tumor protein p53-inducible nuclear protein 1 with L2FC=5.61). Two were annotated to have transport functions, an ATP synthase subunit gene with L2FC= -5.33, and one with an intracellular protein transport protein with L2FC= 3.41. Two were not functionally annotated (Dcitr10g06500.1.1 with L2FC= 5.56, and Dcitr05g06500.1.1 with L2FC= -4.26). Two overlaps between transcripts found in the salivary gland and head datasets included RNA-directed DNA polymerase which is highly down-regulated in *C*Las (+) adults in both datasets, as well as two ATP-synthase transcripts, one up-regulated in salivary glands (ATP synthase gamma chain L2FC=2.56), one down-regulated in heads (ATP synthase delta subunit L2FC= -5.33). The full list of statistically significant (padj<0.05) differentially expressed (L2FC>|2|) head transcripts can be found in Table S6.

Genome improvement leads to quantifiable differences in RNAseq data interpretation.

We hypothesized that, due to improvements in the v3.0 *D. citri* genome, integrating across different datasets for visualization of tissue specific responses may have been successful in part due to improved transcript quantification. To test this hypothesis, the midgut dataset was used to compare RNAseq alignment and DE results between the v.1.1 and v.3.0 *D. citri* genome. The two versions of the *D. citri* genome resulted in different interpretations of the midgut transcriptomics results. Genome v3.0 had a 9% higher overall read alignment, as well as 3000 fewer *D. citri* transcripts found in each biological replicate, on average. After differential

expression, fewer statistically significant (adjusted p-value<0.05) differentially expressed transcripts (Log2FoldChange>|0.5|) were matched to genome v3.0 than genome v1.1. Percent alignment of cleaned reads was less than 100% in all biological replicates for both genomes (Table 2).

Table 2: Comparison of number of raw and trimmed reads from all biological replicates analyzed, as well as percent alignment, number of transcripts, and number of up and down regulated transcripts from both the v1.1 and v3.0 genome analysis of *D. citri* *C*Las (+) midguts.

**Raw read cleaning and filtering stats**						
**Midgut samples**	**#raw paired reads**	**#reads trimmed[1]**	**%aligned v1.1[2]**	**%aligned v3.0[2]**	**#transcripts v1.1[3]**	**#transcript v3.0[3]**
*C*Las(-)_1	27.85M	273	64.89	73.82	17,170	13,814
*C*Las(-)_2	28.26M	234	68.13	77.12	15,284	12,481
*C*Las(-)_3	26.05M	246	66.12	74.29	17,566	14,142
*C*Las(+)_1	26.89M	76	64.04	73.23	16,339	13,281
*C*Las(+)_2	27.15M	210	62.16	71.82	16,834	13,641
*C*Las(+)_3	22.41M	117	64.48	74.77	16,476	13,230

***D. citri* genome v1.1[4]**			***D. citri* genome v3.0[4]**		
**UP**	**DOWN**	**TOTAL**	**UP**	**DOWN**	**TOTAL**
272	341	20,792	176	303	12,704
1.30%	1.64%	100%	1.38%	2.38%	100%

[1]Trimming performed using Trimmomatic to remove adapters and low quality sequences.
[2]Alignment of cleaned reads to each genome performed using Hisat2. Quantities of single- and multi-aligning concordant reads were added together to calculate percent alignment.
[3]Transcripts were counted before differential expression and include only named, annotated Dcitr (v3.0) or XM (v1.1) IDs that have 1 or more counts. Not all transcripts are found in all biological replicates and not all are found in both *C*Las(+) and *C*Las(-).
[4]Differential expression performed via Ballgown and DESeq2. Transcripts in "TOTAL" column have at least 1 read aligning, while UP and DOWN regulated transcripts have adjusted p-value <0.05 and Log2FoldChange>0.5.

Next, we hypothesized several possible ways the genome assembly could impact the interpretation of the transcriptome data (Figure 4A). The orange genome (representing version 1.1, Figure 4A) is shown in short fragments with variably sized gaps between the lengths. The

reads from gene 1 (in blue) demonstrate multi-mapping to more than one genomic region, as well as non-alignment due to missing genomic sequence. The reads in green from gene 2 demonstrate that reads may align across a gap in the genome, and also that a dataset may not have reads to cover all the genome, or, alternatively the genomic sequence is such low quality that reads may not match to it perfectly enough to be counted. The corrected genome from v3.0 (pink) would be predicted to minimize these spurious mapping occurrences (Figure 4A, v3.0 genome in pink).

To test whether these differences between genomes has a measurable effect on downstream expression analyses, we selected four random, differentially expressed transcripts (DE in the v3.0 analysis) for an in-depth comparison (Figure 4B). As predicted, in all four cases, the new gene model was longer and did not contain gaps. In contrast, the associated v1.1 gene models that matched to the full-length transcript were shorter, comprised of more fragments, included introns or gaps (Figure 4B), and were described as "PREDICTED" genes. We matched the read abundance profile over each transcript annotation to demonstrate differences in alignment frequency. The transcript expression associated with each of the v1.1 LOC gene IDs which matched to the sequence from five differentially expressed transcripts from v3.0 (Figure 4B) were assessed relative to v3.0 transcript expression. In all cases, the differential expression of the v1.1 transcripts in *C*Las-exposed relative to healthy was lower and less significant than the expression of the v3.0 transcripts (Table 3).

Figure 4: A) Predicted differences between the version Diaci_1.1 and v3.0 *D. citri* genomes. The genes in blue and green together demonstrate multi-mapping, non-alignment due to missing genomic sequence, alignment across a gap in the genome, and the genomic sequence is such low quality that reads may not match to it perfectly enough to be counted, while the updated genome

represented in pink, fixes or reduces these issues. B) Four example transcripts showing differences in read alignment as a result of differences between the two genome versions. The pink line represents the newest genome v3.0 while orange represents the older genome, v1.1. Dotted lines demonstrate read alignment to the transcripts in the case of each genome.

Table 3: Four statistically significant, differentially expressed genes from v3.0 midgut alignment were subject to BLAST to find their v1.1 genome equivalent gene IDs, and their total read counts, adjusted p-values, and Log2(fold change) are compared.

v3.0 Gene ID	v3.0 padj[1]	V3.0 Log2FC[2]	v1.1 Gene ID	v1.1 padj[1]	v1.1 Log2FC[2]
Dcitr10g01470.1.1	0.00	-10.69	LOC103515983	0.22	-1.21
			LOC103515984	0.50	-0.92
			LOC103518803*	1.00	0.34
Dcitr11g09870.1.1	0.01	-0.511	LOC103518620	0.14	1.60
Dcitr13g03130.1.1	0.01	-0.62	LOC103509242	0.87	-0.21
			LOC103509238	0.86	-0.43
Dcitr13g03190.1.1	0.01	0.51	LOC103513428	0.72	0.66
			LOC103509249	0.84	-0.44
			LOC103509235	0.51	0.56
			LOC113471714	0.55	0.53

[1]Adjusted p-values determined by DESeq2 using Benjamini-Hochberg adjustment of p-values.
[2]Log2FoldChange is calculated relative to healthy, so negative values show reduced expression in *C*Las (+) samples, while positive values show increased expression in *C*Las (+) samples.
*Insufficient read alignment counts for statistical analysis of differential expression.

Discussion

The *D. citri* populations used to generate the samples in this study were infected with *C*Las at different percentages, consistent with what has been reported in the literature [8]. Additionally, *C*Las reads were detected at high levels in the salivary gland and head samples,

consistent with previous studies of the salivary glands using qPCR analysis [7, 9, 10]. The number of *C*Las reads detected in the salivary gland data suggests that *C*Las is transcriptionally active, indicative of replication, though the lack of detection of similar numbers of *C*Las reads in the bacteriome and midgut does not preclude transcription, but that the levels may be below the limit of detection in these samples. Since sample RNA was poly-A enriched using oligos prior to making sequencing libraries, many of the *C*Las transcripts in samples are likely excluded, as poly-A tail enrichment biases samples towards eukaryotic mRNAs. Wang and colleagues recently published in a preprint the idea that the pathology of citrus greening disease is due to cell death of phloem cells triggered by reactive oxygen species. The transcripts upregulated in *C*Las (+) salivary glands suggests that the *D. citri* salivary glands are responding indirectly to the ROS environment of the phloem or directly to the infection of this organ by *C*Las. The highly upregulated transcripts in the *C*Las (+) salivary glands are a hit parade for those involved in response to ROS.

In the salivary glands, the detected *C*Las transcripts had low read counts, most were unannotated, but two transcripts from the fig operon and one from the par operon were detected. The fig operon is part of the flagellum, and is involved in cell motility, cellular processes, chemotaxis, and overall mobility, [41] making it a potentially important gene when *C*Las interacts with its sub-cellular environment in the psyllid. Interestingly, a BLASTx analysis of the coding sequences of both the figB and figC transcripts produced homology to multiple Liberibacter species (figC %identity range of 72.93-84.33%, figB %identity range of 63.08-76.15%). The non-pathogenic *Liberibacter crescens* had the lowest identity (figC % identity = 67.67%, figB % identity = 56.92%) relative to the other Liberibacters, including "*Ca.* L. solanacearum", *Ca.* L. americanus", "*Ca.* L. africanus", "*Ca.* L. europaeus" and "*Ca.* L.

ctenarytainae". These results support the hypothesis that the fig operon may be active in Liberibacter bacteria that are transmitted by pysllids.

Given that the head samples were collected from a different cohort of insects than the salivary gland samples, and the salivary gland transcriptome is expected to be represented to some extent in the head transcriptome, the clustering of the head and salivary gland samples in PC1 was particularly encouraging and shows that transcriptome datasets collected in different experiments can be compared in the same analysis. The excised heads contained multiple organs which *C*Las-infected phloem or saliva pass through including the esophagus, foregut, mouthparts and salivary glands. *C*Las has been found in the brain [17], which is also present in head samples. Thus, the head may contain on average, a greater number of *C*Las bacteria than the other datasets as it contains more organs that *C*Las have been shown to inhabit. However, the head of the psyllid is a highly sclerotized part of the body. Sclerotization may have led to reduced yield when extracting nucleic acids due to reduced disruption efficiency and blockage of filters, two possibilities that may have led to the low yield – both of raw reads and alignment to the *D. citri* genome in these samples. Additionally, it has been shown that eye fluids of insects can contain PCR inhibitors that may interfere with library amplification and sequencing [42, 43].

The parB gene binds DNA and is part of the parABS system, which is known to play a role in bacterial chromosomal partitioning, cell cycle control and cell division, [44] and works by nicking supercoiled plasmid DNA at AT-rich regions and thus can act as a transcriptional regulator. While overall takeaways are limited due to the low number of reads aligned to this *C*Las gene, finding the par operon at relatively high expression when *C*Las is in the salivary glands of *D. citri* is consistent with the hypothesis of bacterial multiplication in this organ. [9]

Due to the low number of *C*Las reads found in the other datasets, parB was not detected and thus relative expression could not be compared across tissues.

Hosseinzadeh et al [17] quantified *C*Las titer in multiple organs of *D. citri* and found that bacteriomes contained a very low titer of *C*Las, with only the reproductive organs showing a lower titer. The bacteriome is highly specialized and designed to provide a place for replication of obligate bacteria. It is encased in a layer of psyllid cells (bacteriocytes), which could act as a barrier to *C*Las entry. Despite the lack of *C*Las in the bacteriome, it still had marked differences in the transcriptome between *C*Las(+) and *C*Las(-). Changes in the transporter genes of the bacteriome may be induced indirectly by changes in leaf protein and small molecule (including amino acid) composition that occurs during citrus greening disease [45-47] or directly by the bacterium during psyllid infection. Another intriguing transcript observed to be differentially expressed in the bacteriome samples is the Dcitr05g01800.11 transcript, which has a log2(fold change) of 2.473, with a length 612 nucleotides, annotated as the "PiggyBac transposable element-derived protein 4". It was significantly differentially expressed in the bacteriome dataset and not the other datasets, suggesting that *C*Las infection of the insect may be inducing transposition in the psyllid bacteriocyte nuclear genome. In the Diaci_v3.0 genome, this transcript is one of at least 11 PiggyBac-related genes found scattered across the genome (see Table S9). The PiggyBac (pB) transposon was first discovered 30 years ago in the cabbage looper, and now it is regularly used to transform insects, such as *Drosophila melanogaster*. PiggyBac is unique among transposases because of its specificity and seamless excision [48]. DNA between two sites with the specific sequence "TTAA" can be cleanly excised and the resulting DNA ends can perfectly match again without leaving a genomic footprint or synthesizing any new DNA. Similarly, the excised transposon can be re-integrated at any TTAA

site in the genome. Due to the precision of pB, it is difficult to know exactly where Dcitr05g01800.11 originated – whether from the syncytial cytoplasmic cells, or the outer bacteriocytes. Considering what is known about pB and the bacteriome interactions with endosymbiotic bacteria, Dcitr05g01800.11 is a strong candidate for future studies of the bacteriome and using pB may open pathways for transgenesis in *D. citri.*

A notable observation is that, although there were low levels of *C*Las reads in the midgut, the impact of *C*Las infection on the *D. citri* transcriptome was greatest in the midgut as compared to other tissues, the former which showed clear separation between *C*Las(+) and *C*Las(-) samples as a result of *C*Las infection. In adult insects, feeding on *C*Las-infected plants has been shown to induce drastic morphological changes to the psyllid nuclear architecture and apoptosis in the midgut epithelial cells [14, 15]. These data suggest that the infected plant sap, and not *C*Las directly, may be playing a role in modulating the midgut transcriptome response. A relatively low replication rate for *C*Las in the midgut vs salivary glands may be an adaptive strategy to switch hosts from plant to insect to evade detection by the psyllid immune system [9, 49] until just prior to transmission to a new host plant.

Lessons learned

Archived transcriptome data is useful and usable together with newly collected data.

PCA analysis enabled a global visualization of the variation both within and across the datasets, and showed that variance due to time of sample collection was minimal. The bi-axis separation between the four datasets as seen in Figure 2 can be partially explained by the average amount of *C*Las present (PC1) and by their sequencing (PC2). The head and bacteriome datasets were collected and multiplexed together but sequenced separately from the midgut and salivary

gland datasets (which were also sequenced at different times). Head and salivary gland samples produced the highest number of reads aligning to *C*Las in the infected biological replicates, and bacteriome and midgut read counts were relatively low.

Transcript quantification accuracy is improved with full-length genome models.

The full-length transcript from the v3.0 analysis was searched against the v1.1 *D. citri* genome using BLAST (see methods). These analyses clearly show how quantification accuracy is improved with the full-length gene models, as all reads matching to a particular transcript are fully accounted for and used for differential expression analysis. Though each of these transcripts being analyzed is relatively short – comprising about 600-4000 nucleotides in length - the difference in read alignment frequency can be in the hundreds. We hypothesized that an improved genome sequence would change how transcriptomics results are interpreted. Analysis of a selected 4 transcripts showed this to be the case. In the v1.1 analysis, all 10 of these fragmented gene IDs and their associated transcripts would have been disregarded from the DE analysis because their adjusted p-values did not meet the significance threshold and the differential expression was nearly nonexistent (L2FC<|1|), and/or counts were too low and lacking in the biological replicates to be used. However, according to the v3.0 analysis, each of the four genes and their transcripts should be considered in downstream pathway analyses of effects of *C*Las exposure as they satisfied the adjusted p-value and log2FoldChange cutoffs. Thus, by quantifying how improved genome assemblies can lead to changes in differential expression, we present evidence to show that long read sequencing or other genome sequence improvement efforts are foundational for transcriptome-wide expression studies. The improvements in overall read alignment rate of the midgut data to the v3.0 genome compared to the v1.1 genome suggests that, during alignment to the v1.1 genome, thousands of *D. citri* reads

were completely left out of the analysis. The lower number of transcripts that matched to genome v3.0 is consistent with the increased scaffold length and gene model improvements.

Improved genome quality did not determine the proportion of transcripts differentially expressed.

Three studies look at the midgut of *D. citri* using transcriptomics: The analysis by Kruse et al. using v1.1 [13], this study using the Kruse et al data and the v3.0 genome, and a study by Yu et al. [35] using the v2.0 genome. The source of the midgut RNA is significantly different between the Yu et al. study and the Kruse et al study. Yu et al pooled midguts from *D. citri* adults raised on *Murraya exotica*, whereas Kruse et al. and thus, the current study, utilized insects raised on *Citrus medica*. Yu et al. also reported different *C*Las-infection rates among their individual insects pooled compared to Kruse et al. The relative proportions of transcripts that are up or down regulated in each of the three studies is not consistent, nor does the pattern become consistent with improved genome quality. In studies by Yu et al. and Kruse et al., there are more up regulated transcripts (499 and 965 respectively) than down regulated transcripts (279 and 850 respectively), while in this current study, the opposite is true (176 up and 303 down) (Table S3). The midgut analysis by Kruse et al. aligned RNA reads to the *D. citri* genome assembly v1.1 using the bioinformatic tools RSEM and bowtie2 for alignment, followed by edgeR and DESeq2 for differential expression calculations. The raw data from Kruse et al. was reanalyzed in the current study using the most recent versions of the bioinformatic tools Hisat2 (genome alignment), Stringtie (transcript assembly), Ballgown and DESeq2 (differential expression). These two bioinformatic pipelines differ in their alignment algorithms, statistical methods, and importantly their ability to identify false positive and negative differentially expressed

transcripts. The proportion of differentially expressed transcripts may be derived from the biology of the organisms or samples and in part the bioinformatic pipelines.

_C_Las-exposed or unexposed are the most precise descriptions of _D. citri_ reared on HLB positive or uninfected citrus.

Detection of *C*Las reads in some tissues and not others leads us to revisit the nomenclature used to describe insects which are sampled from *C*Las-infected plants. Some studies, such as this one, designate insect samples as *C*Las (+) or *C*Las (-), or healthy or infected referring to the infection status of the tree used to rear the insect. Alternatively, some studies label insects (as opposed to the trees) as *C*Las-exposed or unexposed, the latter when sampled from healthy, *C*Las-negative trees. The use of exposed or unexposed is to account for the finding that not all insects acquire and/or become infected with *C*Las when reared on *C*Las-infected trees [8, 50, 51]. This transcriptomics study suggests that the exposed and unexposed designations are the most accurate because there is deeper complexity of *C*Las infection status in each insect at the level of the organ. In this study, salivary glands appear to have 10x more *C*Las reads than found in midguts and even more than in bacteriomes, suggesting salivary glands are truly “infected” and other organs, such as the bacteriome, remain “exposed”.

Bacterial transcript counts are not reliable to determine infection of a psyllid tissue with _C_Las.

It was difficult to interpret whether psyllid organs were infected based on read count alone when read counts were barely above background, such as in the midguts. Kruse et al [13] reported that 82% (n=20, Cq<40) of the *C*Las (+) *D. citri* population which was harvested for their midguts were positive for *C*Las with an average qPCR Cq value of 31 across their four *C*Las (+) biological replicates. While 212 is not an especially large number of *C*Las reads post

poly-A enrichment, when paired with the qPCR results, midguts, which have been shown to contain a visible slurry of *C*Las cells in previous work using microscopy [13, 15], may be referred to as “infected” by *C*Las, but at a lower level than the salivary glands. However, similar number of *C*Las reads were detected in the head samples from insects sampled from healthy (unexposed) trees as in the midguts, so whether the *C*Las reads in the midguts are meaningful is debatable. Finding a low level of reads aligning to *C*Las in healthy samples is not unexpected, and may be due to a few understandable reasons, such as a lack of enrichment of bacterial transcripts following poly-A enrichment for eukaryotic mRNAs, alignment errors, genome annotation errors, or homology of these reads to other psyllid-associated bacteria (the bacterial endosymbionts). *C*Las (-) psyllid colonies and citrus plants are reared in separate but identical environments to *C*Las (+) trees and insects. It is critical that all insect materials are tested regularly and thoroughly for *C*Las using qPCR to rule out the possibility of unintended *C*Las infection *C*Las (-) samples prior to experimentation.

Potential Implications

*C*Las is uncultivable and methods to study *C*Las-*D. citri* interactions are challenging. Genome sequencing is a foundational tool for our exploration of the molecular interactions among *D. citri*, *C*Las, the bacterial endosymbionts and the citrus host. Our research showed that improved genome assemblies influences interpretation of transcriptomic data and that investigators have reason to re-analyze their previous *D. citri* transcriptomic data with the new genome release. The more accurate quantification provided by the Diaci_v3.0 genome may reduce the need to validate transcriptomic changes using reverse transcription (RT)-PCR. We urge arthropod genome communities and funding bodies to continue to invest funds on genome

improvement projects such as i5k [52] and Ag100Pest [53]. These investments can help save expenditures elsewhere by reanalyzing previously generated and yielding higher confidence in the results after using a quality genome backbone. Additionally, single-cell RNAseq is the next frontier of understanding insect-pathogen interactions, especially for intracellular symbionts, at the highest resolution. Currently, single-cell RNAseq has been done on very few insects, but the list is expanding [54-57].

Still, a major roadblock is the functional annotation of the gene models. While automated pipelines for annotation exist at NCBI and elsewhere [58], these efforts are supplemented by manual annotation efforts [59-63] for *D. citri* and other arthropods [52]. Future work on understanding how the improved genome leads to improved quantification at the proteome level is also needed, and we hope such studies are inspired by the findings we present here. Our analysis demonstrates that it is possible to analyze new 'omics data in the context of and alongside historical data in public repositories to maximize the use of existing large-scale dataset resources in discovering new biology. The results underscore the importance of chromosomal length assemblies of arthropod genomes for accurate interpretation of gene expression.

Methods

***C*Las titer determination by qPCR.**

*D. citri C*Las-exposed and unexposed colonies were tested for the presence of *C*Las using qPCR by amplification of the 16S rDNA using TaqMan reagents. Individual, whole-body, adult psyllids (n=50 for the midgut colony, n=20 for the salivary gland colony, n=20 for the colony used to collect heads and bacteriomes) were collected from each colony. Total DNA was

extracted from individual insects using the Qiagen DNeasy kit. DNA concentration was measured using a Nanodrop spectrophotometer. Each sample was standardized to 30 ng/ul so the Cq values from each dataset can be compared directly subjected. The *C*Las probe (5'-FAM-AGACGGGTG/ZEN/AGTAACGCG-3') sequence and specific forward (5'-TCGAGCGCGTATGCAATACG-3') and reverse (5'-GCGTTATCCCGTAGAAAAAGGTAG-3') primers used are as published previously in Kruse et al. [13]. Unexposed colonies were tested monthly and *C*Las (+) colonies were tested at the time the insects were collected for dissection. Each qPCR plate contained positive and negative controls as well as a CLas 16S rDNA standard curve to allow for both absolute and relative *C*Las titer quantification, and every sample was run in triplicate. For our purposes, only Cq values were required to determine to whether individual samples were *C*Las (-/+) and to record the percent infection rate (how many out of 20 were *C*Las (+)) of the colony. A sample was considered *C*Las (+) if the Cq value was <40 (if there is only a single molecule in the reaction, with perfect primer efficiency, 37-40 cycles will be the cycle plateau). The Cq data from all 20 individuals, from all three colonies (bacteriomes and heads were collected from the same individuals and thus the same colony) was compiled and reported in Figure S1. Cq values from the CLas unexposed insects were undetected.

Once colonies were confirmed *C*Las (+) or *C*Las(-) by qPCR, hundreds of one to two week-old adult *D. citri* were collected and pooled to create biological replicates. Midgut samples included 250 guts pooled per biological replicate [n=3 replicates each *C*Las(+) and *C*Las(-)], salivary gland replicates each included 150 pooled extirpations [n=4 replicates each *C*Las(+) and *C*Las(-)], while 120 bacteriomes and heads were pooled for each replicate [n=5 replicates each *C*Las(+) and *C*Las(-)]. Salivary glands and midguts were pooled in TriZol while bacteriomes and heads were pooled in beta-mercaptoethanol and Qiagen RLT buffer. Samples were stored at -

80C until RNA extraction. The paired-end 150bp Illumina sequencing, raw data was uploaded to NCBI and is accessible to reviewers via BioProject accession # PRJNA385527, submission ID SUB10382129 and will be made available to the public upon publication.

***In silico* quality control and cleaning of raw data to reduce confounding factors in analysis.**

Data analysis was conducted on servers hosted by the Computational Biology Center at the Boyce Thompson Institute. Data for all four datasets (bacteriome, head, salivary gland and midgut) were subjected to identical computational assessments and manipulations to eliminate variability caused by analysis methods. Total raw mRNA reads were first analyzed with FastQC [64] to gauge the presence of anomalies and adapters. Illumina Universal adapters that were present were removed by first interleaving/merging together forward and reverse reads into one large file. This file was then presented to AdapterRemover [65] using the Unix commands suggested in the manual for PE analysis. AdapterRemover output a file of interleaved paired-end reads that survived adapter removal. FastQC was run for the second time on this file to confirm adapter removal and check remaining read lengths and total remaining read quantity. This interleaved file was then used as input for SortMeRNA [66] which removes rRNA that survived the poly-A enrichment *in silico,* based on rRNA databases for bacteria, eukaryotes and archaea provided with the software program. Seed length was adjusted from default 18 down to 14 during rRNA database file indexing to be compatible with the minimum length reads in the current data set. SortMeRNA supplied two output types: 1) Those reads that mapped to rRNA (both forward and reverse reads had to map to be included), and 2) those where one or both of the paired end reads did not map to rRNA, such that the non-rRNA read pool contained some single strand sequences that aligned to rRNA. Separating out rRNA reduced over expression and bias of ribosomal gene expression in the datasets without totally removing rRNAs from the

analysis. Low quality sequences (QC<20) were removed with Trimmomatic [67]. Paired reads where one or more are shorter than 17 nucleotides were then discarded. FastQC was run for the third time on these files to check their new read length distribution, read number and overall quality. A shell script was used to unmerge the forward and reverse reads for each sample file (reverse interleaving), creating a set of paired-end data files containing "cleaned reads" that could be used in the following steps.

Read alignment to multiple genomes and differential transcript expression for each dataset.

All four datasets comprised of cleaned, paired-end mRNA reads were aligned to both the v3.0 *D. citri* genome and the "*Candidatus* Liberibacter asiaticus" psy62 genome available on NCBI. The midgut dataset was additionally aligned to the v1.1 *D. citri* genome. The computational methods closely follow those published by Pertea et al., [68] and include the following: Each *D. citri* genome was indexed using HISAT2 (*hisat2-build*) [69]. Total cleaned reads were aligned to the indexed genome using *hisat2* and standard settings for PE data as described in the HISAT2 manual [69]. Specifically, options added to the base function included index memory mapping (*--mm*); setting the number of server threads to increase the speed of the alignment (*-p*); specifying output file names for both concordant alignments and non-concordant alignments (*--al-conc* and *--un-conc*, respectively); specifying which of the input files was forward or reverse (specified by "RF" showing -1 was reverse and -2 was forward); and tailored the output file organization for the possibility of downstream transcript assembly (*--dta*). Additionally, read alignment statistics were directed into a .stdout file for ease of future reference. Reads that aligned concordantly (collected in the *--al-conc* output file) were checked with FastQC and used in the next steps. Following alignment, the SAM files were converted to BAM to save space and then sorted by name using SAMtools [70]. Once sorted, reads were

bundled into transcripts using Stringtie [71] based on their alignments and promptly re-aligned to the .GTF/.GFF file specific to each genome, containing information on all known genes for that genome. This process labeled each transcript with a specific Gene_ID, genomic location and information on introns/exons. Finally, using the number of transcripts that align to each gene, a count matrix was formed using Stringtie and ballgown [68] to allow downstream differential expression (DE) analysis between *C*Las (-) and *C*Las (+) replicates, paired with data visualization. Differential expression was performed in R (v3.3.3) using DESeq2 [72], following standard protocols (DE determined by setting *C*Las (-) as the denominator such that positive Log2FoldChange (L2FC) indicates greater expression in *C*Las (+) replicates and negative L2FC indicates reduced expression in *C*Las (+) replicates relative to *C*Las (-)). Because each dataset (except bacteriome and head) was collected and sequenced separately, normalizing the datasets to each other had too many experimental variables that were uncontrollable, so DE analysis for *C*Las (-/+) was performed separately for each dataset. DE results, like those of the qPCR Cq data, could be compared directly for transcripts within a dataset, while transcripts across datasets could be qualified, though no direct or quantitative comparison of expression could be made between datasets. Reads that aligned to *C*Las in the *C*Las (+) samples were counted and only certain transcripts of interest were analyzed further.

Statistics and data visualization of results.

A variety of statistical methods and data visualization tools were utilized. A principal components analysis (PCA) of all four datasets combined was performed in R (*prcomp* and *plot*) using a large transcript count matrix combining the transcript expression count matrices from the four datasets. The count data was minimally normalized by transcript counts per million and transcripts not present in both *C*Las (-) and *C*Las (+) replicates were removed. Individual PCA

plots were also generated in R (*plotPCA* and *ggplot*) to show separation between *C*Las (-) and *C*Las (+) biological replicates, using the DESeq2 rlog-transformed transcript data for each dataset individually. Following PCA analysis, R was used to generate Volcano plots of the differentially expressed transcripts from each dataset individually, again using the DESeq2 rlog-transformed data. The L2FC of each DE transcript was plotted against the negative log of the adjusted p-value (-log(padj)) for the same transcript using *ggplot*.

The comparison of expression results from the midgut dataset when aligned to either v3.0 or v1.1 of the *D. citri* genome was started by choosing four transcripts present and expressed in both analyses. The two genomes presented different gene_IDs and genomic location coordinates which was problematic for direct comparison of changes in expression or even direct comparison of transcripts. The transcript sequence from Diaci_v3.0 was analyzed using BLASTx against the v1.1 genome to determine which v1.1 transcripts aligned to the v3.0 transcript and whether alignment was partial or full. To demonstrate differences in read distribution between the two genomes for each of the four transcripts and to show differential alignment frequencies, the v3.0 transcript sequences and associated v1.1 transcript sequences were used as a genome backbone and total cleaned reads were re-aligned to these sequences using HISAT2 to generate the .BAM files of read alignments for each transcript. Coverage maps were generated for each transcript using an R script (*BEDtools*) written by Dave Tang [73]. The general pattern of coverage from these coverage plots was duplicated in cartoon form on top of the respective transcript cartoon, to demonstrate the differences in read alignment location and frequency between the two *D. citri* genomes.

Potential secreted effectors were determined from the list of top DE transcripts of the salivary gland dataset by running two programs – SignalP-v5.0 [74] which accesses protein

sequences for the presence of signal peptides, and Phobius [75] which detects both signal peptides and transmembrane helices (TMHs) from a protein sequence. Transcripts that putatively contained signal peptides but not TMHs were considered candidate salivary gland effector proteins.

Additional Information Sections

Data Submission Information for Reviewers:

Title specific to data itself: The RNAseq data for the transcriptome analysis of multiple *D. citri* organs including the head, bacteriome and salivary gland.

Abstract: Paired-end 150bp mRNA-seq raw read files generated from pools of organs, which may include contaminants and/or other endosymbionts in addition to *Candidatus* Liberibacter asiaticus. Data is ideal for differential gene expression analysis.

Author list: Marina Mann, Surya Saha, Lukas A. Mueller, Michelle Heck

Data types: poly-A enriched RNA, i.e transcriptome data

Organisms/Tissues of each data type: All data from *Diaphorina citri*, tissues include gut, bacteriome, salivary gland, and head including thoracic segment 1 and antennae.

Estimate of dataset size: 120 G

File organization: Tar archive named “Dcitri_SG_BAC_HEAD_mRNA.tar” which contains three sub-archives in the following order, called “BACarchive.tar”, “HEADarchive.tar”, “SGarchive.tar”. Each sub archive contains gzipped fastq files for forward and reverse of every biological replicate.

Acknowledgments: Funding to generate samples and sequence them from Michelle Heck and Lukas Mueller, USDA-NIFA grants 2015-70016-23028 and 2020-70029-33199.

Declarations

List of abbreviations

All abbreviations have been defined in the manuscript.

Consent for publication

Not Applicable.

Competing interests

The author(s) declare that they have no competing interests.

Funding

This project was funded by NIFA Predoctoral Fellowship 2021-67011-35143 (MM) USDA-NIFA grants 2015-70016-23028 (MH and LM), 2020-70029-33199 (LM) and USDA ARS Project number 8062-22410-007-00-D (MH).

Authors' contributions

MM: Took part in, or led, all aspects including conceptualization, data curation, formal analysis, funding acquisition, investigation, methodology, validation, visualization and writing of original draft as well as review and editing.

SS: Funding acquisition, conceptualization, methodology, resources, writing - review and editing.

JMC: Visualization, writing – review and editing, data curation.

MP: Methodology, resources, software, writing – review and editing.

KM: Data curation, resources.

LC: Funding acquisition, project administration, resources, supervision.

WBH: Funding acquisition, project administration, resources, supervision, writing – review and editing.

LAM: Funding acquisition, project administration, methodology, conceptualization resources, supervision, writing – review and editing.

MH: Conceptualization, investigation, methodology, project administration, resources, supervision, validation, visualization, writing of original draft and reviews and edits.

Acknowledgements

We thank Jaclyn Mahoney (Cornell University) for assistance with lab work, Dr. Angela Kruse (now at Vanderbilt University) for teaching Marina Mann how to extract RNA from psyllid organs while Dr. Kruse was a graduate student at Cornell, Tracy Bell and Hanna Mann from IRREC at Fort Pierce, FL for their assistance with excision of salivary glands. We are also grateful to Dr. Robert Krueger at the USDA ARS Citrus Germplasm Repository for providing the Heck lab with pathogen-free citrus seeds.

References

1. Wang N, Stelinski LL, Pelz-Stelinski KS, Graham JH and Zhang Y. Tale of the Huanglongbing Disease Pyramid in the Context of the Citrus Microbiome. Phytopathology. 2017;107 4:380-7. doi:10.1094/PHYTO-12-16-0426-RVW.
2. Wang N, Pierson EA, Setubal JC, Xu J, Levy JG, Zhang Y, et al. The *Candidatus* Liberibacter-Host Interface: Insights into Pathogenesis Mechanisms and Disease Control. Annu Rev Phytopathol. 2017;55:451-82. doi:10.1146/annurev-phyto-080516-035513.
3. Ammar E-D, Jr RGS and Heck M. 8 Huanglongbing Pathogens: Acquisition, Transmission and Vector Interactions. Asian Citrus Psyllid: Biology, Ecology and Management of the Huanglongbing Vector. 2020:113.
4. McRoberts N, Dunn R and Deniston-Sheets H. Mining value from ACP prevalance data. Citrograph. 2021;12 1:38-41.
5. Lee JA, Halbert SE, Dawson WO, Robertson CJ, Keesling JE and Singer BH. Asymptomatic spread of huanglongbing and implications for disease control. Proc Natl Acad Sci U S A. 2015;112 24:7605-10. doi:10.1073/pnas.1508253112.
6. Meng L, Li X, Cheng X and Zhang H. 16S rRNA Gene Sequencing Reveals a Shift in the Microbiota of *Diaphorina citri* During the Psyllid Life Cycle. Front Microbiol. 2019;10:1948. doi:10.3389/fmicb.2019.01948.
7. Ammar E-D, Ramos JE, Hall DG, Dawson WO and Shatters RG, Jr. Acquisition, Replication and Inoculation of *Candidatus* Liberibacter asiaticus following Various

Acquisition Periods on Huanglongbing-Infected Citrus by Nymphs and Adults of the Asian Citrus Psyllid. PLoS One. 2016;11 7:e0159594. doi:10.1371/journal.pone.0159594.

8. Ammar ED, Hall DG, Hosseinzadeh S and Heck M. The quest for a non-vector psyllid: Natural variation in acquisition and transmission of the huanglongbing pathogen '*Candidatus* Liberibacter asiaticus' by Asian citrus psyllid isofemale lines. PLoS One. 2018;13 4:e0195804. doi:10.1371/journal.pone.0195804.

9. Ammar ED, Shatters RG and Hall DG. Localization of *Candidatus* Liberibacter asiaticus, Associated with Citrus Huanglongbing Disease, in its Psyllid Vector using Fluorescence in situ Hybridization. J Phytopathol. 2011;159 11-12:726-34. doi:DOI 10.1111/j.1439-0434.2011.01836.x.

10. Ammar E, Shatters RG, Lynch C and Hall DG. Detection and Relative Titer of *Candidatus* Liberibacter asiaticus in the Salivary Glands and Alimentary Canal of Diaphorina citri (Hemiptera: Psyllidae) Vector of Citrus Huanglongbing Disease. Ann Entomol Soc Am. 2011;104 3:526-33. doi:10.1603/AN10134.

11. Brown JK, Cicero, J.M. and Fisher, T.W. Psyllid-transmitted *Candidatus* Liberibacter species infecting citrus and solanaceous hosts. St. Paul, Minnesota: American Phytopathological Society; 2016.

12. JM C. Functional anatomy of the Asian citrus psyllid. In: Qureshi JaSP, editor. Asian citrus psyllid: Biology, ecology and management of the huanglongbing vector. CAB International; 2020.

13. Kruse A, Fattah-Hosseini S, Saha S, Johnson R, Warwick E, Sturgeon K, et al. Combining 'omics and microscopy to visualize interactions between the Asian citrus psyllid vector and the Huanglongbing pathogen Candidatus Liberibacter asiaticus in the insect gut. PLoS One. 2017;12 6:e0179531. doi:10.1371/journal.pone.0179531.

14. Ghanim M, Fattah-Hosseini S, Levy A and Cilia M. Morphological abnormalities and cell death in the Asian citrus psyllid (*Diaphorina citri*) midgut associated with *Candidatus* Liberibacter asiaticus. Sci Rep. 2016;6:33418. doi:10.1038/srep33418.

15. Mann M, Fattah-Hosseini S, Ammar ED, Stange R, Warrick E, Sturgeon K, et al. *Diaphorina citri* Nymphs Are Resistant to Morphological Changes Induced by "*Candidatus* Liberibacter asiaticus" in Midgut Epithelial Cells. Infect Immun. 2018;86 4 doi:10.1128/IAI.00889-17.

16. Ghanim M, Achor D, Ghosh S, Kontsedalov S, Lebedev G and Levy A. '*Candidatus* Liberibacter asiaticus' Accumulates inside Endoplasmic Reticulum Associated Vacuoles in the Gut Cells of *Diaphorina citri*. Sci Rep. 2017;7 1:16945. doi:10.1038/s41598-017-16095-w.

17. Hosseinzadeh S, Shams-Bakhsh M, Mann M, Fattah-Hosseini S, Bagheri A, Mehrabadi M, et al. Distribution and Variation of Bacterial Endosymbiont and "*Candidatus* Liberibacter asiaticus" Titer in the Huanglongbing Insect Vector, Diaphorina citri Kuwayama. Microb Ecol. 2019;78 1:206-22. doi:10.1007/s00248-018-1290-1.

18. Dossi FC, da Silva EP and Consoli FL. Population dynamics and growth rates of endosymbionts during *Diaphorina citri* (Hemiptera, Liviidae) ontogeny. Microb Ecol. 2014;68 4:881-9. doi:10.1007/s00248-014-0463-9.

19. Saha S, Hunter WB, Reese J, Morgan JK, Marutani-Hert M, Huang H, et al. Survey of endosymbionts in the *Diaphorina citri* metagenome and assembly of a *Wolbachia* wDi draft genome. PLoS One. 2012;7 11:e50067. doi:10.1371/journal.pone.0050067.

20. Chu CC, Gill TA, Hoffmann M and Pelz-Stelinski KS. Inter-Population Variability of Endosymbiont Densities in the Asian Citrus Psyllid (*Diaphorina citri* Kuwayama). Microb Ecol. 2016;71 4:999-1007. doi:10.1007/s00248-016-0733-9.
21. Nakabachi A, Ueoka R, Oshima K, Teta R, Mangoni A, Gurgui M, et al. Defensive bacteriome symbiont with a drastically reduced genome. Current biology : CB. 2013;23 15:1478-84. doi:10.1016/j.cub.2013.06.027.
22. Guidolin AS and Consoli FL. Molecular characterization of *Wolbachia* strains associated with the invasive Asian citrus psyllid *Diaphorina citri* in Brazil. Microb Ecol. 2013;65 2:475-86. doi:10.1007/s00248-012-0150-7.
23. Morrow JL, Om N, Beattie GAC, Chambers GA, Donovan NJ, Liefting LW, et al. Characterization of the bacterial communities of psyllids associated with *Rutaceae* in Bhutan by high throughput sequencing. BMC Microbiol. 2020;20 1:215. doi:10.1186/s12866-020-01895-4.
24. Flores-Gonzalez M, Hosmani PS, Fernandez-Pozo N, Mann M, Humann JL, Main D, et al. Citrusgreening.org: An open access and integrated systems biology portal for the Huanglongbing (HLB) disease complex. bioRxiv. 2019:868364. doi:10.1101/868364.
25. Reese J, Christenson MK, Leng N, Saha S, Cantarel B, Lindeberg M, et al. Characterization of the Asian Citrus Psyllid Transcriptome. J Genomics. 2014;2:54-8. doi:10.7150/jgen.7692.
26. Saha S, Hosmani PS, Villalobos-Ayala K, Miller S, Shippy T, Flores M, et al. Improved annotation of the insect vector of citrus greening disease: biocuration by a diverse genomics community. Database (Oxford). 2017;2017:bax032-bax. doi:10.1093/database/bax032.
27. Prashant S. Hosmani MF-G, Teresa Shippy, Chad Vosburg, Crissy Massimino, Will Tank, Max Reynolds, Blessy Tamayo, Sherry Miller, Jordan Norus, Kyle Kercher, Bec Grace, Margaryta Jernigan, Doug Harper, Sam Adkins, Yesmarie DeLaFlor, Thomson Paris, Sara Vandervoort, Rebekah Adams, Seantel Norman, Jessica Ventura, Michael Perry, Matthew Weirauch, Josh Benoit, Wayne B. Hunter, Helen Wiersma-Koch, Tom D'elia, Susan Brown, Lukas A. Mueller and Surya Saha. Chromosomal length reference assembly for *Diaphorina citri* using single-molecule sequencing and Hi-C proximity ligation with manually curated genes in developmental, structural and immune pathways. biorxiv. 2020; doi:https://doi.org/10.1101/869685
28. Leng N, English A, Johnson S, Richards S, Hunter W and Saha S. *Diaphorina citri* genome assembly Diaci 1.1. 2017.
29. Fleites LA, Johnson R, Kruse AR, Nachman RJ, Hall DG, MacCoss M, et al. Peptidomics Approaches for the Identification of Bioactive Molecules from *Diaphorina citri*. J Proteome Res. 2020;19 4:1392-408. doi:10.1021/acs.jproteome.9b00509.
30. Hosseinzadeh S, Ramsey J, Mann M, Bennett L, Hunter WB, Shams-Bakhsh M, et al. Color morphology of *Diaphorina citri* influences interactions with its bacterial endosymbionts and 'Candidatus Liberibacter asiaticus'. PLoS One. 2019;14 5:e0216599. doi:10.1371/journal.pone.0216599.
31. Kruse A, Ramsey JS, Johnson R, Hall DG, MacCoss MJ and Heck M. Candidatus Liberibacter asiaticus Minimally Alters Expression of Immunity and Metabolism Proteins in Hemolymph of *Diaphorina citri*, the Insect Vector of Huanglongbing. J Proteome Res. 2018;17 9:2995-3011. doi:10.1021/acs.jproteome.8b00183.
32. Ramsey JS, Johnson RS, Hoki JS, Kruse A, Mahoney J, Hilf ME, et al. Metabolic Interplay between the Asian Citrus Psyllid and Its Profftella Symbiont: An Achilles' Heel of the

Citrus Greening Insect Vector. PLoS One. 2015;10 11:e0140826. doi:10.1371/journal.pone.0140826.

33. Wu Z, Pu X, Shu B, Bin S and Lin J. Transcriptome analysis of putative detoxification genes in the Asian citrus psyllid, *Diaphorina citri*. Pest Manag Sci. 2020;76 11:3857-70. doi:10.1002/ps.5937.

34. Wu ZZ, Qu MQ, Chen MS and Lin JT. Proteomic and transcriptomic analyses of saliva and salivary glands from the Asian citrus psyllid, *Diaphorina citri*. J Proteomics. 2021;238:104136. doi:10.1016/j.jprot.2021.104136.

35. Yu HZ, Li NY, Zeng XD, Song JC, Yu XD, Su HN, et al. Transcriptome Analyses of Diaphorina citri Midgut Responses to *Candidatus* Liberibacter Asiaticus Infection. Insects. 2020;11 3 doi:10.3390/insects11030171.

36. Alba-Tercedor J, Hunter WB and Alba-Alejandre I. Using micro-computed tomography to reveal the anatomy of adult *Diaphorina citri* Kuwayama (Insecta: Hemiptera, Liviidae) and how it pierces and feeds within a citrus leaf. Sci Rep. 2021;11 1:1358. doi:10.1038/s41598-020-80404-z.

37. Cicero JM and Brown JK. A stationary tweezer platform for high throughput dissections of minute arthropods and extirpation of their minute organs. MethodsX. 2021;8:101317.

38. Rio DC, Ares M, Jr., Hannon GJ and Nilsen TW. Purification of RNA using TRIzol (TRI reagent). Cold Spring Harb Protoc. 2010;2010 6:pdb prot5439. doi:10.1101/pdb.prot5439.

39. Zhong S, Joung JG, Zheng Y, Chen YR, Liu B, Shao Y, et al. High-throughput illumina strand-specific RNA sequencing library preparation. Cold Spring Harb Protoc. 2011;2011 8:940-9. doi:10.1101/pdb.prot5652.

40. Duan Y, Zhou L, Hall DG, Li W, Doddapaneni H, Lin H, et al. Complete genome sequence of citrus huanglongbing bacterium, '*Candidatus* Liberibacter asiaticus' obtained through metagenomics. Mol Plant Microbe Interact. 2009;22 8:1011-20. doi:10.1094/MPMI-22-8-1011.

41. Homma M, Kutsukake K, Hasebe M, Iino T and Macnab RM. FlgB, FlgC, FlgF and FlgG. A family of structurally related proteins in the flagellar basal body of *Salmonella typhimurium*. J Mol Biol. 1990;211 2:465-77. doi:10.1016/0022-2836(90)90365-S.

42. Boncristiani H, Li J, Evans JD, Pettis J and Chen Y. Scientific note on PCR inhibitors in the compound eyes of honey bees, *Apis mellifera*. Apidologie. 2011;42 4:457-60. doi:10.1007/s13592-011-0009-9.

43. Schrader C, Schielke A, Ellerbroek L and Johne R. PCR inhibitors - occurrence, properties and removal. J Appl Microbiol. 2012;113 5:1014-26. doi:10.1111/j.1365-2672.2012.05384.x.

44. Jalal ASB and Le TBK. Bacterial chromosome segregation by the ParABS system. Open Biol. 2020;10 6:200097. doi:10.1098/rsob.200097.

45. Ramsey JS, Chin EL, Chavez JD, Saha S, Mischuk D, Mahoney J, et al. Longitudinal Transcriptomic, Proteomic, and Metabolomic Analysis of *Citrus limon* Response to Graft Inoculation by *Candidatus* Liberibacter asiaticus. J Proteome Res. 2020;19 6:2247-63. doi:10.1021/acs.jproteome.9b00802.

46. Chin EL, Ramsey JS, Mishchuk DO, Saha S, Foster E, Chavez JD, et al. Longitudinal Transcriptomic, Proteomic, and Metabolomic Analyses of Citrus sinensis (L.) Osbeck Graft-Inoculated with "*Candidatus* Liberibacter asiaticus". J Proteome Res. 2020;19 2:719-32. doi:10.1021/acs.jproteome.9b00616.

47. Killiny N and Nehela Y. Metabolomic Response to Huanglongbing: Role of Carboxylic Compounds in Citrus sinensis Response to '*Candidatus* Liberibacter asiaticus' and Its Vector, Diaphorina citri. Mol Plant Microbe Interact. 2017;30 8:666-78. doi:10.1094/MPMI-05-17-0106-R.
48. Q Chen WL, RA Veach, AB Hickman, M Wilson, F Dyda. Structural basis of seamless excision and specific targeting by *piggyBac* transposase. Nature Communications. 2020;11 3446.
49. Yan Q, Sreedharan A, Wei S, Wang J, Pelz-Stelinski K, Folimonova S, et al. Global gene expression changes in *Candidatus* Liberibacter asiaticus during the transmission in distinct hosts between plant and insect. Mol Plant Pathol. 2013;14 4:391-404. doi:10.1111/mpp.12015.
50. Hall D. Incidence of "*Candidatus* Liberibacter asiaticus" in a Florida population of Asian citrus psyllid. Journal of Applied Entomology. 2018;142 1-2:97-103.
51. Coy M and Stelinski LL. Great Variability in the Infection Rate of '*Candidatus* Liberibacter Asiaticus' in Field Populations of *Diaphorina citri* (Hemiptera: Liviidae) in Florida. Florida Entomologist. 2015;98 1:356-7.
52. Poelchau M, Childers C, Moore G, Tsavatapalli V, Evans J, Lee CY, et al. The i5k Workspace@NAL--enabling genomic data access, visualization and curation of arthropod genomes. Nucleic Acids Res. 2015;43 Database issue:D714-9. doi:10.1093/nar/gku983.
53. Childers AK, Geib SM, Sim SB, Poelchau MF, Coates BS, Simmonds TJ, et al. The USDA-ARS Ag100Pest Initiative: High-Quality Genome Assemblies for Agricultural Pest Arthropod Research. Insects. 2021;12 7 doi:10.3390/insects12070626.
54. Severo MS, Landry JJM, Lindquist RL, Goosmann C, Brinkmann V, Collier P, et al. Unbiased classification of mosquito blood cells by single-cell genomics and high-content imaging. Proc Natl Acad Sci U S A. 2018;115 32:E7568-E77. doi:10.1073/pnas.1803062115.
55. Traniello IM, Bukhari SA, Kevill J, Ahmed AC, Hamilton AR, Naeger NL, et al. Meta-analysis of honey bee neurogenomic response links *Deformed wing virus type A* to precocious behavioral maturation. Sci Rep. 2020;10 1:3101. doi:10.1038/s41598-020-59808-4.
56. Raddi G, Barletta ABF, Efremova M, Ramirez JL, Cantera R, Teichmann SA, et al. Mosquito cellular immunity at single-cell resolution. Science. 2020;369 6507:1128-32. doi:10.1126/science.abc0322.
57. Feng M, Xia J, Fei S, Peng R, Wang X, Zhou Y, et al. Identification of Silkworm Hemocyte Subsets and Analysis of Their Response to Baculovirus Infection Based on Single-Cell RNA Sequencing. Front Immunol. 2021;12:645359. doi:10.3389/fimmu.2021.645359.
58. Surya Saha AMC, Anna K Childers, Monica F Poelchau, Fiona M McCarthy. Workflows for rapid functional annotation of diverse arthropod genomes. bioRxiv. 2021.06.12.448177 doi:https://doi.org/10.1101/2021.06.12.448177
59. Vosburg C, Reynolds M, Noel R, Shippy T, Hosmani PS, Flores-Gonzalez M, et al. Utilizing a chromosomal-length genome assembly to annotate the Wnt signaling pathway in the Asian citrus psyllid, *Diaphorina citri*. Gigabyte. 2021;2021:1-15. doi:10.46471/gigabyte.21.
60. Sharma P, Al-Dossary O, Alsubaie B, Al-Mssallem I, Nath O, Mitter N, et al. Improvements in the sequencing and assembly of plant genomes. Gigabyte. 2021;2021:1-10. doi:10.46471/gigabyte.24.

61. Miller S, Shippy TD, Hosmani PS, Flores-Gonzalez M, Mueller LA, Hunter WB, et al. Annotation of segmentation pathway genes in the Asian citrus psyllid, *Diaphorina citri*. Gigabyte. 2021;2021:1-13. doi:10.46471/gigabyte.26.
62. Miller S, Shippy TD, Tamayo B, Hosmani PS, Flores-Gonzalez M, Mueller LA, et al. In silico characterization of chitin deacetylase genes in the *Diaphorina citri* genome. Gigabyte. 2021;2021:1-11. doi:10.46471/gigabyte.25.
63. Miller S, Shippy TD, Tamayo B, Hosmani PS, Flores-Gonzalez M, Mueller LA, et al. Annotation of chitin biosynthesis genes in *Diaphorina citri*, the Asian citrus psyllid. Gigabyte. 2021;2021:1-12. doi:10.46471/gigabyte.23.
64. Bioinformatics B: FastQC v0.11.8. https://www.bioinformatics.babraham.ac.uk/projects/fastqc/. Accessed Dec 29 2019.
65. Schubert M, Lindgreen S and Orlando L. AdapterRemoval v2: rapid adapter trimming, identification, and read merging. BMC Res Notes. 2016;9 1:88. doi:10.1186/s13104-016-1900-2.
66. Kopylova: SortMeRNA v2.1b. http://bioinfo.lifl.fr/RNA/sortmerna (2014).
67. Bolger AM, Lohse M and Usadel B. Trimmomatic: a flexible trimmer for Illumina sequence data. Bioinformatics. 2014;30 15:2114-20. doi:10.1093/bioinformatics/btu170.
68. Pertea M, Kim D, Pertea GM, Leek JT and Salzberg SL. Transcript-level expression analysis of RNA-seq experiments with HISAT, StringTie and Ballgown. Nat Protoc. 2016;11 9:1650-67. doi:10.1038/nprot.2016.095.
69. Kim D, Paggi JM, Park C, Bennett C and Salzberg SL. Graph-based genome alignment and genotyping with HISAT2 and HISAT-genotype. Nat Biotechnol. 2019;37 8:907-15. doi:10.1038/s41587-019-0201-4.
70. Li: Samtools – Utilities for the Sequence Alignment/Map (SAM) format. https://github.com/samtools/samtools (2018).
71. Kovaka S, Zimin AV, Pertea GM, Razaghi R, Salzberg SL and Pertea M. Transcriptome assembly from long-read RNA-seq alignments with StringTie2. Genome Biol. 2019;20 1:278. doi:10.1186/s13059-019-1910-1.
72. Love MI, Huber W and Anders S. Moderated estimation of fold change and dispersion for RNA-seq data with DESeq2. Genome Biol. 2014;15 12:550. doi:10.1186/s13059-014-0550-8.
73. D T: Creating a coverage plot using BEDTools and R. https://davetang.org/muse/2015/08/05/creating-a-coverage-plot-using-bedtools-and-r/ (2015). Accessed July 2 2021.
74. Almagro Armenteros JJ, Tsirigos KD, Sonderby CK, Petersen TN, Winther O, Brunak S, et al. SignalP 5.0 improves signal peptide predictions using deep neural networks. Nat Biotechnol. 2019;37 4:420-3. doi:10.1038/s41587-019-0036-z.
75. Kall L, Krogh A and Sonnhammer EL. Advantages of combined transmembrane topology and signal peptide prediction--the Phobius web server. Nucleic Acids Res. 2007;35 Web Server issue:W429-32. doi:10.1093/nar/gkm256.

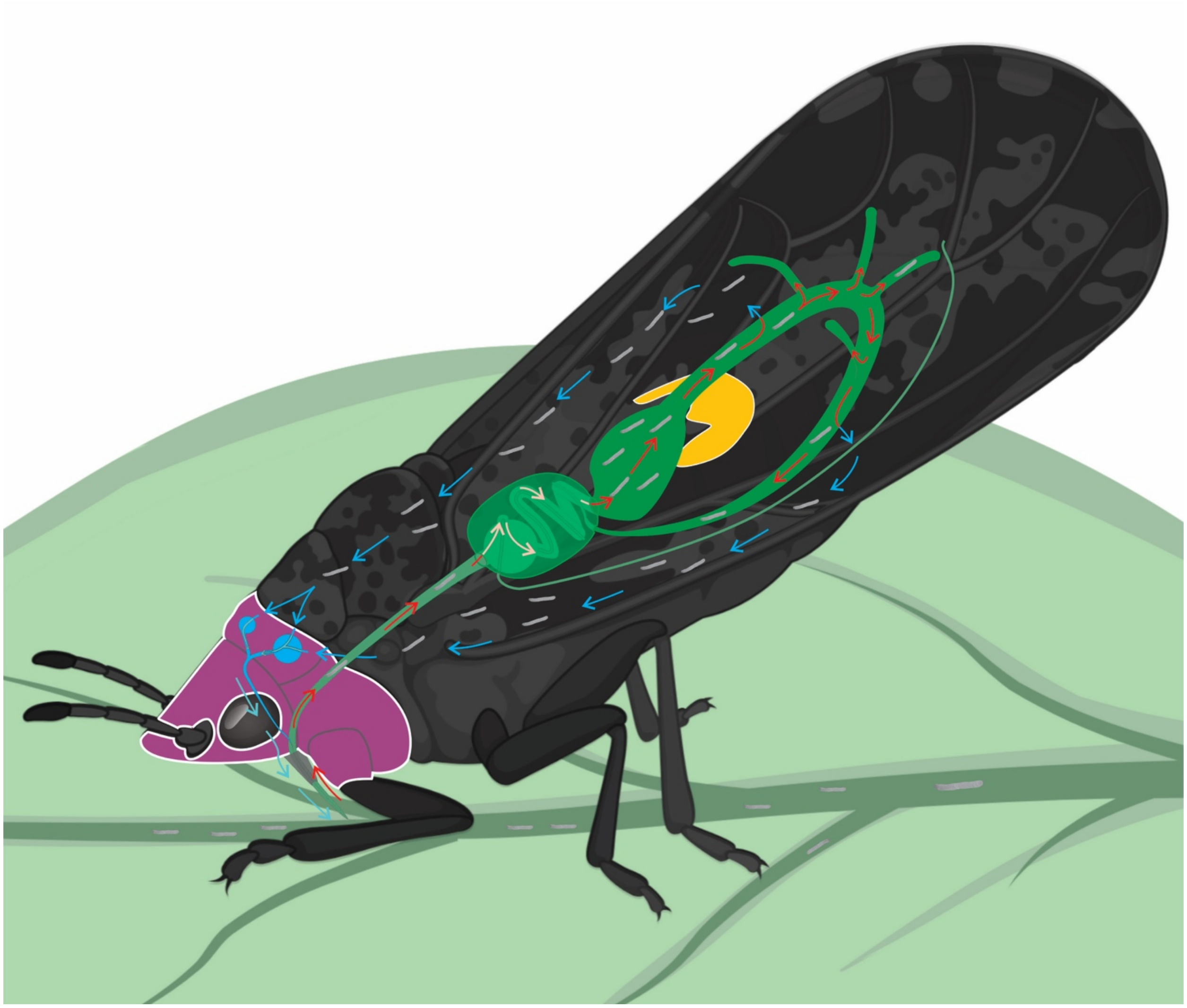

Figure 1

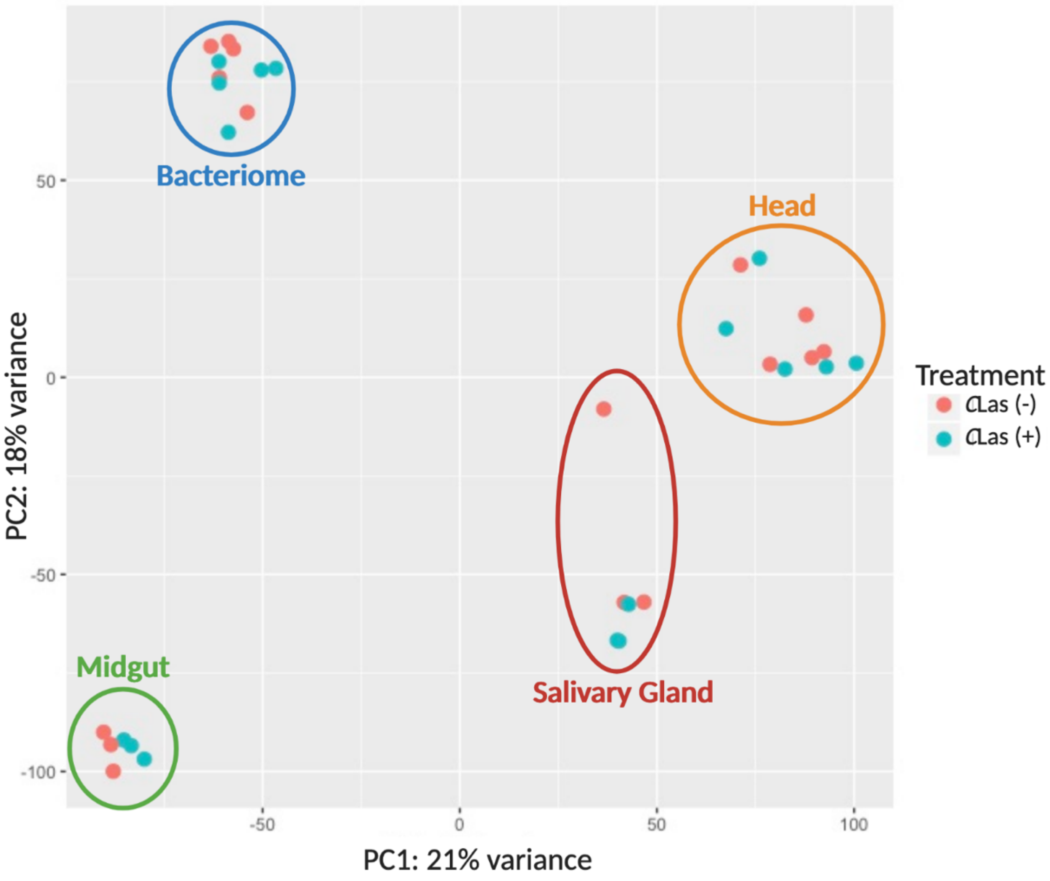
Bacteriome
Head
Salivary Gland
Midgut
Treatment
CLas (-)
CLas (+)
PC2: 18% variance
PC1: 21% variance
50
0
-50
-100
-50
0
50
100

Figure 2

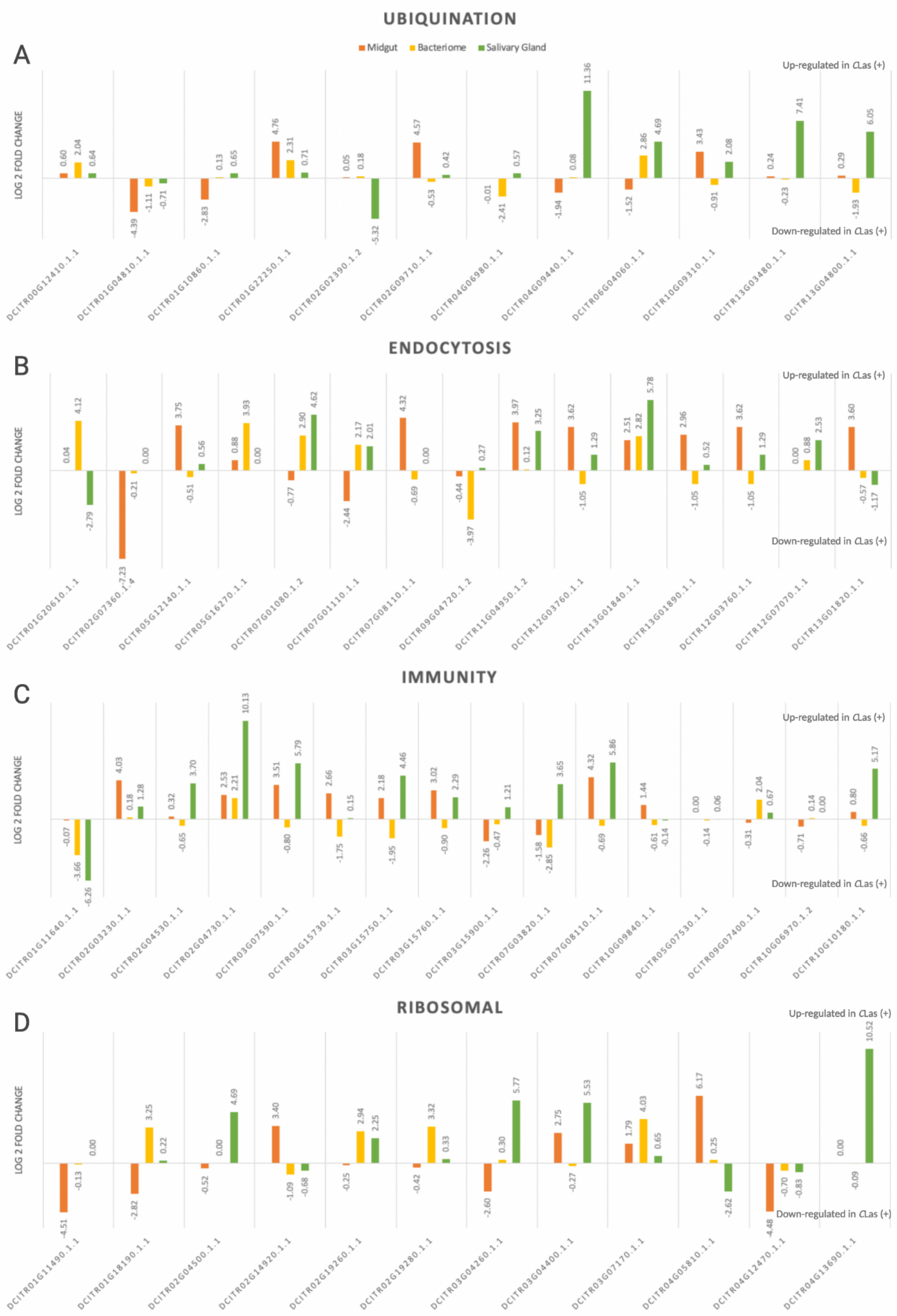
UBIQUINATION
A
Midgut
Bacteriome
Salivary Gland
Up-regulated in CLas (+)
Down-regulated in CLas (+)
LOG 2 FOLD CHANGE
0.60 2.04 0.64
-4.39 -1.11 -0.71
-2.83 0.13 0.65
4.76 2.31 0.71
0.05 0.18 -5.32
4.57 -0.53 0.42
-0.01 -2.41 0.57
-1.94 0.08 11.36
-1.52 2.86 4.69
3.43 -0.91 2.08
0.24 -0.23 7.41
0.29 -1.93 6.05
DCITR00G12410.1.1
DCITR01G04810.1.1
DCITR01G10860.1.1
DCITR01G22250.1.1
DCITR02G02390.1.2
DCITR02G09710.1.1
DCITR04G06980.1.1
DCITR04G09440.1.1
DCITR06G04060.1.1
DCITR10G09310.1.1
DCITR13G03480.1.1
DCITR13G04800.1.1
ENDOCYTOSIS
B
Up-regulated in CLas (+)
Down-regulated in CLas (+)
LOG 2 FOLD CHANGE
0.04 4.12 -2.79
-7.23 -0.21 0.00
3.75 -0.51 0.56
0.88 3.93 0.00
-0.77 2.90 4.62
-2.44 2.17 2.01
4.32 -0.69 0.00
-0.44 -3.97 0.27
3.97 0.12 3.25
3.62 -1.05 1.29
2.51 2.82 5.78
2.96 -1.05 0.52
3.62 -1.05 1.29
0.00 0.88 2.53
3.60 -0.57 -1.17
DCITR01G20610.1.1
DCITR02G07360.1.4
DCITR05G12140.1.1
DCITR05G16270.1.1
DCITR07G01080.1.2
DCITR07G01110.1.1
DCITR07G08110.1.1
DCITR09G04720.1.2
DCITR11G04950.1.2
DCITR12G03760.1.1
DCITR13G01840.1.1
DCITR13G01890.1.1
DCITR12G03760.1.1
DCITR12G07070.1.1
DCITR13G01820.1.1
IMMUNITY
C
Up-regulated in CLas (+)
Down-regulated in CLas (+)
LOG 2 FOLD CHANGE
-0.07 -3.66 -6.26
4.03 0.18 1.28
0.32 -0.65 3.70
2.53 2.21 10.13
3.51 -0.80 5.79
2.66 -1.75 0.15
2.18 -1.95 4.46
3.02 -0.90 2.29
-2.26 -0.47 1.21
-1.58 -2.85 3.65
4.32 -0.69 5.86
1.44 -0.61 -0.14
0.00 -0.14 0.06
-0.31 2.04 0.67
-0.71 0.14 0.00
0.80 -0.66 5.17
DCITR01G11640.1.1
DCITR02G03230.1.1
DCITR02G04530.1.1
DCITR02G04730.1.1
DCITR03G07590.1.1
DCITR03G15730.1.1
DCITR03G15750.1.1
DCITR03G15760.1.1
DCITR03G15900.1.1
DCITR07G03820.1.1
DCITR07G08110.1.1
DCITR10G09840.1.1
DCITR05G07530.1.1
DCITR09G07400.1.1
DCITR10G06970.1.2
DCITR10G10180.1.1
RIBOSOMAL
D
Up-regulated in CLas (+)
Down-regulated in CLas (+)
LOG 2 FOLD CHANGE
-4.51 -0.13 0.00
-2.82 3.25 0.22
-0.52 0.00 4.69
3.40 -1.09 -0.68
-0.25 2.94 2.25
-0.42 3.32 0.33
-2.60 0.30 5.77
2.75 -0.27 5.53
1.79 4.03 0.65
6.17 0.25 -2.62
-4.48 -0.70 -0.83
0.00 -0.09 10.52
DCITR01G11490.1.1
DCITR01G18190.1.1
DCITR02G04500.1.1
DCITR02G14920.1.1
DCITR02G19260.1.1
DCITR02G19280.1.1
DCITR03G04260.1.1
DCITR03G04400.1.1
DCITR03G07170.1.1
DCITR04G05810.1.1
DCITR04G12470.1.1
DCITR04G13690.1.1

Figure 3

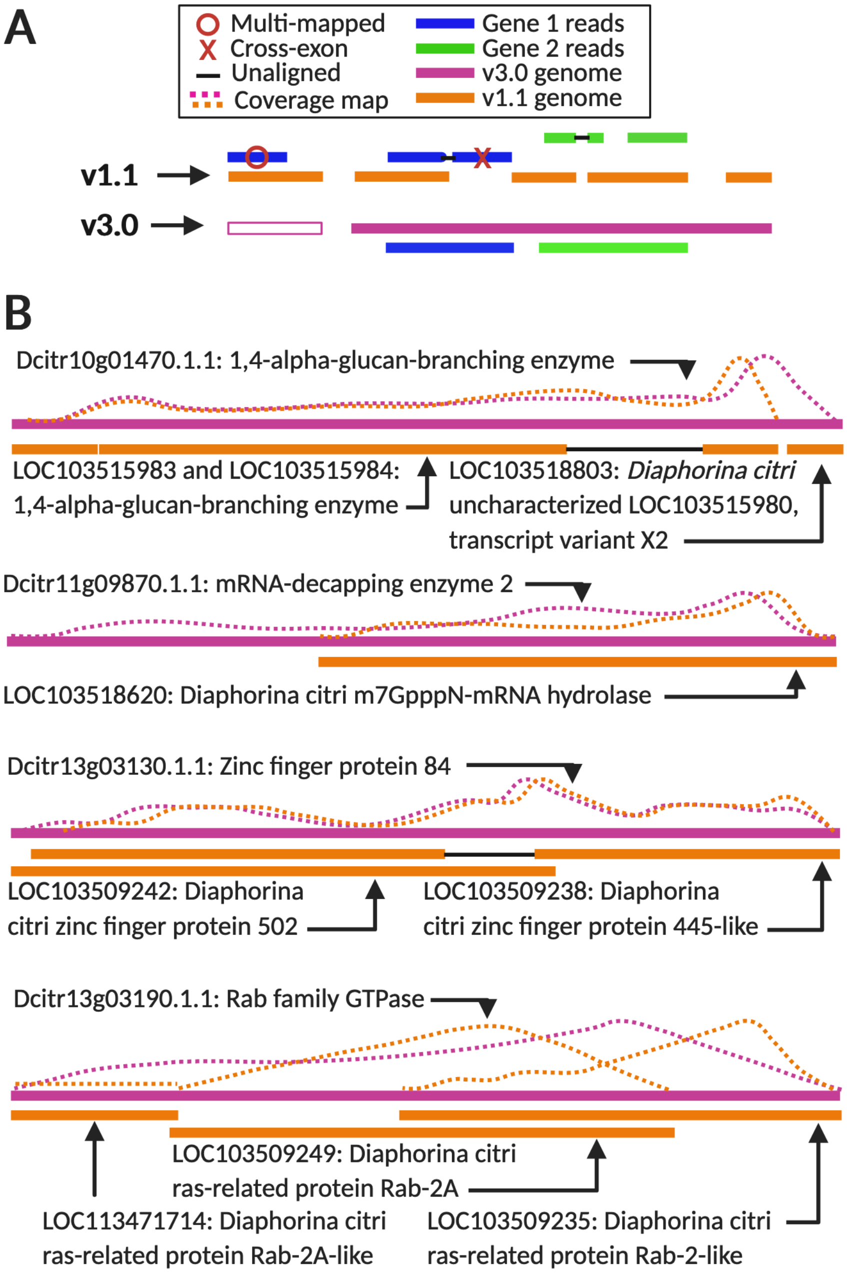
A
Multi-mapped
Cross-exon
Unaligned
Coverage map
Gene 1 reads
Gene 2 reads
v3.0 genome
v1.1 genome
v1.1
v3.0
B
Dcitr10g01470.1.1: 1,4-alpha-glucan-branching enzyme
LOC103515983 and LOC103515984: 1,4-alpha-glucan-branching enzyme
LOC103518803: Diaphorina citri uncharacterized LOC103515980, transcript variant X2
Dcitr11g09870.1.1: mRNA-decapping enzyme 2
LOC103518620: Diaphorina citri m7GpppN-mRNA hydrolase
Dcitr13g03130.1.1: Zinc finger protein 84
LOC103509242: Diaphorina citri zinc finger protein 502
LOC103509238: Diaphorina citri zinc finger protein 445-like
Dcitr13g03190.1.1: Rab family GTPase
LOC103509249: Diaphorina citri ras-related protein Rab-2A
LOC113471714: Diaphorina citri ras-related protein Rab-2A-like
LOC103509235: Diaphorina citri ras-related protein Rab-2-like

Figure 4

Figure S1: A histogram of *C*Las Cq values from individuals tested from each colony used to generate RNAseq data.

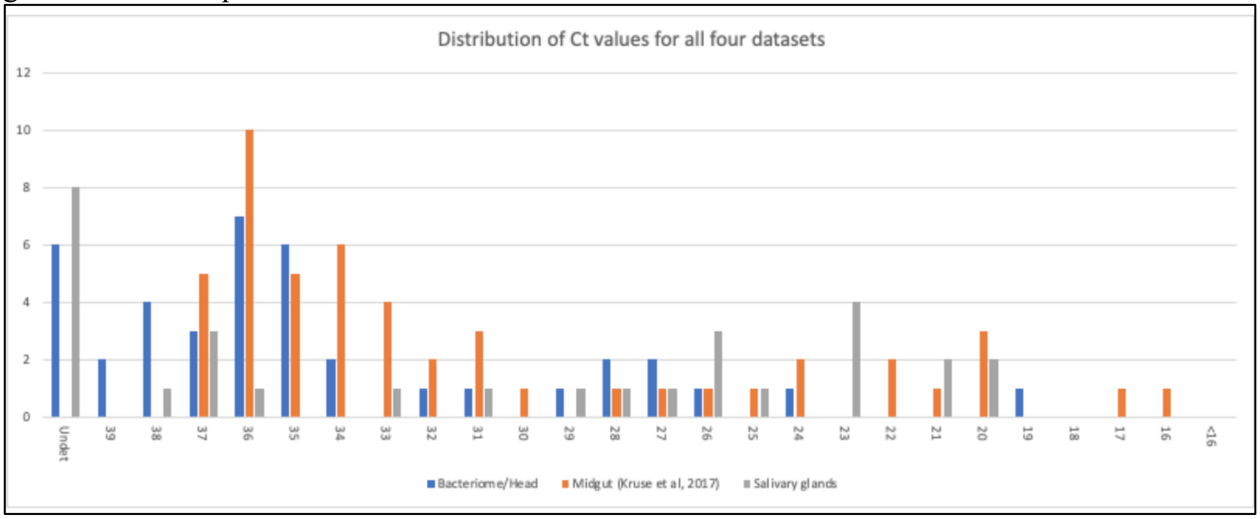

Figure S2: Principal components analysis of all four datasets relative to each other, and distinguishing between healthy and *C*Las-exposed biological replicates.

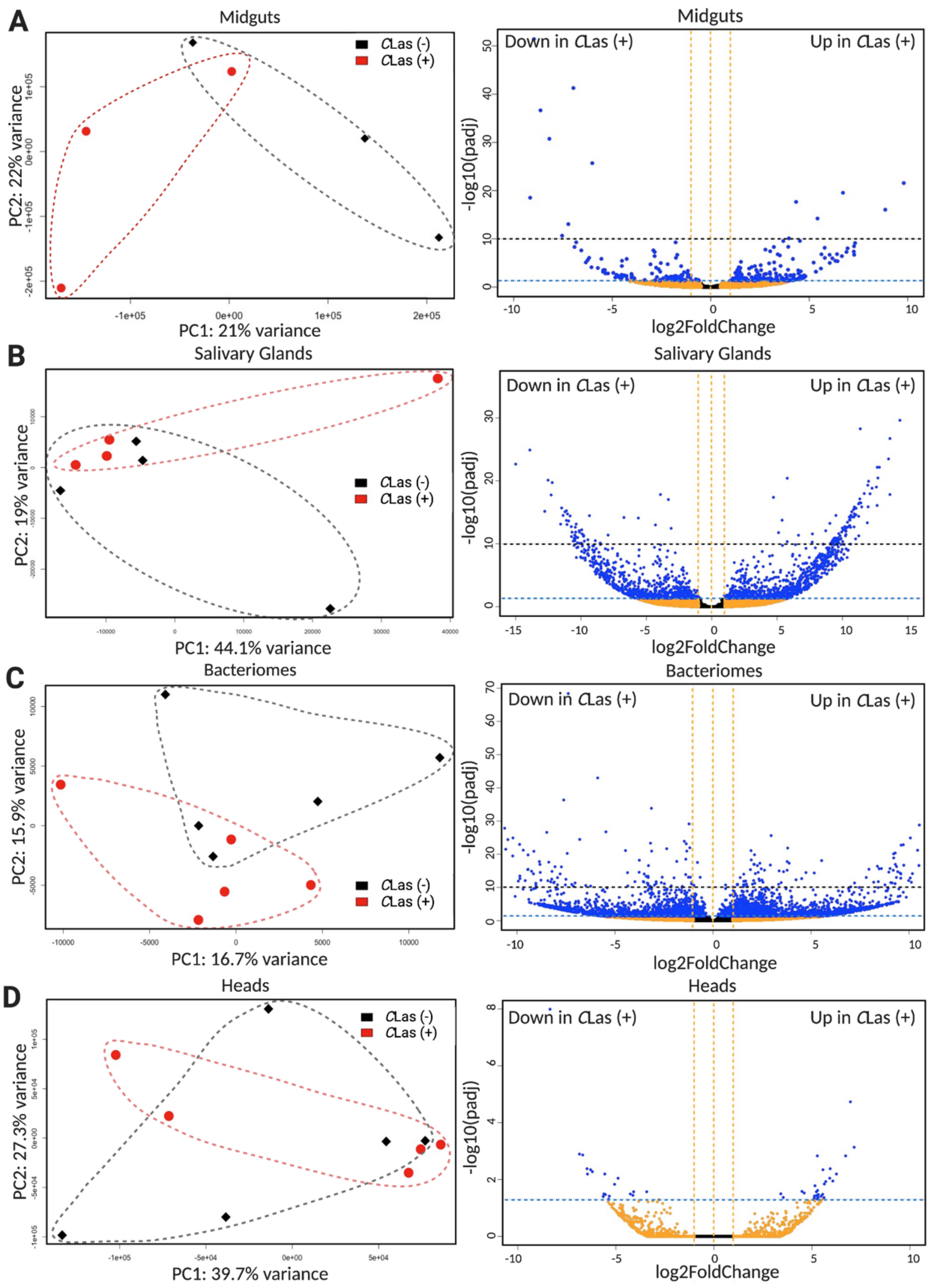

Table S1: Of the *C*Las transcripts identified from the salivary gland *C*Las (+) transcriptome dataset, only those *C*Las transcripts with reads present in three or more of the four biological replicates were considered (n=56). Among the top 10 with the most reads aligned, three transcripts stood out based on their annotation, figC, figB (two most abundant) and parB (9th most abundant). All others are annotated as "unknown". Reads were aligned to the CLas-psy62 genome from NCBI.

**Order**	**Gene_ID**	**Total # reads aligned**	**Annotation**
1	gene269	290.00	figB
2	gene268	176.00	figC
3	gene911	166.00	Unknown
4	gene702	148.00	Unknown
5	gene1020	127.00	Unknown
6	gene1056	126.00	Unknown
7	gene788	87.00	Unknown
8	gene543	77.00	Unknown
9	gene416	71.00	parB
10	gene993	80.00	Unknown

Table S2: Metadata on RNAseq datasets and alignments.

GUT	# reads	%3.0	SG	# reads	%3.0	BAC	# reads	%3.0	HEA.	# reads	%3.0
*C*Las (-) 1	27.85	73.82	*C*Las (-) 1	50.85	59.02	*C*Las (-) 1	25.29	78.74	*C*Las (-) 1	17.34	92.80
*C*Las (-) 2	28.26	77.12	*C*Las (-) 2	40.55	75.31	*C*Las (-) 2	25.18	82.01	*C*Las (-) 2	14.10	36.59
*C*Las (-) 3	26.05	74.29	*C*Las (-) 3	52.21	71.42	*C*Las (-) 3	20.71	78.78	*C*Las (-) 3	6.32	83.53
			*C*Las (-) 4	16.27	82.11	*C*Las (-) 4	21.19	81.29	*C*Las (-) 4	22.73	91.38
						*C*Las (-) 5	20.94	82.41	*C*Las (-) 5	12.03	13.15
*C*Las (+) 1	26.89	73.23	*C*Las (+) 1	54.25	72.07	*C*Las (+) 1	24.12	79.77	*C*Las (+) 1	5.97	10.39
*C*Las (+) 2	27.15	71.82	*C*Las (+) 2	59.21	73.29	*C*Las (+) 2	19.82	81.12	*C*Las (+) 2	16.82	39.71
*C*Las (+) 3	22.41	74.77	*C*Las (+) 3	67.56	70.54	*C*Las (+) 3	20.28	81.44	*C*Las (+) 3	16.31	85.37
			*C*Las (+) 4	84.31	84.31	*C*Las (+) 4	24.61	81.47	*C*Las (+) 4	24.41	89.70
						*C*Las (+) 5	18.95	84.15	*C*Las (+) 5	18.00	21.72
Avg.	26.43	74.17		44.98	73.51		22.11	81.12		15.40	56.43

GUT=Midgut dataset, SG=Salivary gland dataset, BAC=Bacteriome dataset, HEA.=Head dataset
#reads = The number millions of raw, paired-end reads.
%3.0 = The percent of paired-end reads that aligned to the version 3.0 *D. citri* genome.

Table S3: All transcripts from the midgut dataset that have differential expression log2FoldChange>|2| and adjusted p-value<0.05. Sorted by Log2FoldChange. Aligned to v3.0 of the *D. citri* genome.

**Transcript_ID**	**L2FC**	**padj**	**Annotation**
Dcitr10g01470.1.1	9.797	0.000	1,4-alpha-glucan-branching enzyme
Dcitr08g09790.1.1	8.859	0.000	Surfeit locus 4
Dcitr08g09800.1.1	7.327	0.000	SET and MYND domain-containing protein 4
Dcitr02g10600.1.1	7.283	0.000	Xanthine dehydrogenase
Dcitr08g03160.1.1	7.275	0.000	Murein tripeptide amidase MpaA
Dcitr03g01380.1.1	7.262	0.000	Alpha-mannosidase
Dcitr06g15560.1.2	6.946	0.000	centrosomal protein of 135 kDa, partial
Dcitr01g13610.1.1	6.891	0.000	Unknown protein
Dcitr03g07140.1.1	6.712	0.000	Acyl-CoA dehydrogenase
Dcitr08g02010.1.1	6.656	0.000	Open rectifier potassium channel protein 1
Dcitr02g11990.1.1	6.435	0.000	Gamma-glutamyl hydrolase
Dcitr04g05420.1.1	6.408	0.000	Unknown protein
Dcitr05g07620.1.1	6.329	0.000	Vinculin
Dcitr04g05810.1.1	6.173	0.000	28S ribosomal protein S18b, mitochondrial
Dcitr08g09260.1.2	6.039	0.000	Solute carrier family 41 member 2
Dcitr10g02030.1.1	6.034	0.000	UDP-glucuronosyltransferase
Dcitr12g06530.1.1	5.791	0.000	Unknown protein
Dcitr02g11330.1.1	5.783	0.000	DnaJ subfamily B member 2
Dcitr08g09460.1.3	5.618	0.000	Lipoma HMGIC fusion partner-like 2 protein
Dcitr09g06950.1.2	5.592	0.000	Nucleobase
Dcitr11g06490.1.1	5.420	0.000	zinc finger CCCH domain-containing protein 13-like
Dcitr01g06150.1.1	5.338	0.001	Mid1-interacting protein 1
Dcitr01g06150.1.2	5.338	0.001	Mid1-interacting protein 1
Dcitr03g09200.1.1	5.221	0.000	Metalloendopeptidase
Dcitr02g05500.1.2	4.803	0.006	neuropeptide CNMamide
Dcitr01g22250.1.1	4.762	0.008	RBR-type E3 ubiquitin transferase
Dcitr07g08080.1.1	4.744	0.004	Cysteine proteinase
Dcitr03g14920.1.5	4.724	0.005	GATA zinc finger domain-containing protein 15-like, partial
Dcitr01g12010.1.2	4.714	0.008	Pancreatic lipase-related protein 2
Dcitr08g09760.1.1	4.704	0.008	Insulin-like growth factor 2 mRNA-binding protein 3
Dcitr08g08680.1.1	4.701	0.013	Rho GTPase-activating protein 190
Dcitr06g06560.1.1	4.698	0.006	Intraflagellar transport particle protein 88
Dcitr03g17770.1.1	4.629	0.011	Unknown protein
Dcitr02g09070.1.1	4.625	0.016	Carboxypeptidase D
Dcitr02g09710.1.1	4.571	0.001	E3 ubiquitin ligase PARAQUAT TOLERANCE 3-like
Dcitr11g08160.1.1	4.568	0.005	Unknown protein
Dcitr10g01310.1.1	4.557	0.013	Ankyrin repeat protein
Dcitr02g07600.1.1	4.524	0.000	Enolase
Dcitr01g12820.1.1	4.478	0.013	ran-binding protein 3-like
Dcitr04g04620.1.1	4.452	0.001	Unknown protein
Dcitr02g07110.1.1	4.442	0.000	Phosphate transporter
Dcitr04g09180.1.3	4.411	0.023	selection and upkeep of intraepithelial T-cells protein 1-like isoform X2
Dcitr03g11060.1.1	4.333	0.000	ATP-dependent RNA helicase
Dcitr07g08110.1.1	4.323	0.026	Cathepsin

Dcitr01g13440.1.1	4.281	0.026	Unknown protein
Dcitr11g03350.1.1	4.240	0.033	Alpha-tocopherol transfer protein-like
Dcitr00g02160.1.1	4.224	0.003	CG12123-RA
Dcitr04g06690.1.1	4.205	0.017	Long-chain acyl-CoA synthetase
Dcitr01g01630.1.2	4.190	0.008	Bestrophin homolog
Dcitr01g05670.1.1	4.187	0.036	Lachesin
Dcitr01g18090.1.1	4.183	0.033	Unknown protein
Dcitr02g09760.1.1	4.176	0.033	Unknown protein
Dcitr11g01690.1.1	4.172	0.000	Unknown protein
Dcitr07g07340.1.1	4.162	0.033	circadian locomoter output cycles protein kaput
Dcitr04g09730.1.1	4.107	0.036	Odorant-binding protein 18
Dcitr01g09430.1.1	4.059	0.004	Protein prgI
Dcitr07g10360.1.1	4.029	0.035	Ankyrin repeat and fibronectin type-III domain-containing protein 1
Dcitr04g11360.1.2	4.028	0.004	Proton-coupled amino acid transporter 4
Dcitr02g03230.1.1	4.027	0.004	CLIPC3 -Serine Protease Snake-Like.
Dcitr07g06300.1.1	3.998	0.041	General secretion pathway protein C
Dcitr11g04950.1.2	3.967	0.000	Dynamin-like protein, mitochondrial
Dcitr00g04570.1.3	3.934	0.000	Zinc
Dcitr03g09760.1.1	3.922	0.000	UDP-glucuronosyltransferase
Dcitr03g11640.1.1	3.880	0.049	2,3-bisphosphoglycerate-dependent phosphoglycerate mutase
Dcitr00g01710.1.1	3.858	0.012	Major
Dcitr10g07760.1.1	3.822	0.003	Unknown protein
Dcitr10g02970.1.1	3.776	0.024	Unknown protein
Dcitr04g11510.1.1	3.774	0.029	Unknown protein
Dcitr05g12140.1.1	3.750	0.040	Vacuolar protein sorting-associated protein 37A
Dcitr05g13670.1.1	3.685	0.000	Dosage compensation regulator
Dcitr02g13200.1.1	3.647	0.030	Unknown protein
Dcitr06g07920.1.1	3.633	0.012	Unknown protein
Dcitr12g03760.1.1	3.621	0.017	Cathepsin L1-like Prot 4
Dcitr01g08080.1.2	3.614	0.014	Hydroxymethylglutaryl-CoA lyase
Dcitr13g01820.1.1	3.602	0.018	SITE-1 protease
Dcitr04g06130.1.1	3.547	0.000	LYR motif-containing protein 7
Dcitr12g03060.1.1	3.509	0.004	Unknown protein
Dcitr03g07590.1.1	3.508	0.000	serine/threonine-protein phosphatase 4 regulatory subunit 3
Dcitr10g09310.1.1	3.431	0.047	Ubiquitin-conjugating enzyme
Dcitr02g14920.1.1	3.400	0.000	Adenine phosphoribosyltransferase
Dcitr05g04040.1.1	3.362	0.015	Tumor protein p53-inducible nuclear protein 1
Dcitr02g12750.1.1	3.333	0.033	Mediator of RNA polymerase II transcription subunit 9
Dcitr03g05350.1.1	3.331	0.006	Protein bric-a-brac 2
Dcitr13g02990.1.1	3.310	0.001	Unknown protein
Dcitr07g10650.1.1	3.301	0.027	transcription factor SPT20 homolog, partial
Dcitr08g11710.1.2	3.241	0.000	3-hydroxy-3-methylglutaryl coenzyme A synthase
Dcitr00g05130.1.1	3.220	0.000	Unknown
Dcitr06g09810.1.1	3.208	0.036	WD repeat-containing protein on Y chromosome
Dcitr08g05230.1.1	3.202	0.000	Arylsulfatase B
Dcitr03g07160.1.1	3.173	0.033	La
Dcitr03g03770.1.1	3.062	0.021	GTP-binding protein 1
Dcitr02g20050.1.1	3.062	0.015	Tetratricopeptide repeat (TPR)-like superfamily protein
Dcitr07g03880.1.1	3.046	0.049	Unknown protein
Dcitr03g15760.1.1	3.022	0.014	Trypsin-like serine protease

Dcitr02g11140.1.1	3.017	0.000	keratin-associated protein 19-2-like isoform X1
Dcitr10g07700.1.1	3.016	0.002	Unknown protein
Dcitr08g07480.1.3	3.000	0.001	villin-2-like
Dcitr13g01890.1.1	2.963	0.000	Adenylate kinase isoenzyme 6 homolog
Dcitr10g07150.1.1	2.902	0.017	Heat Shock Protein 70 A1
Dcitr02g07960.1.1	2.806	0.002	Aquaporin AQPAe.a-like
Dcitr10g06510.1.1	2.774	0.000	Heat Shock Protein 70 B
Dcitr01g22530.1.1	2.746	0.018	Unknown protein
Dcitr11g01900.1.1	2.713	0.002	Alpha-(1,6)-fucosyltransferase
Dcitr08g03010.1.1	2.676	0.010	Peritrophin-1
Dcitr04g17010.1.1	2.659	0.012	Histone H3
Dcitr06g02580.1.1	2.599	0.019	Cobalt-zinc-cadmium efflux system protein
Dcitr08g07480.1.4	2.557	0.000	villin-2-like
Dcitr13g03230.1.1	2.535	0.000	cytochrome B561%2C amino-terminal protein Chr1:2469528-2472518 REVERSE LENGTH%3D693 201606
Dcitr13g01840.1.1	2.513	0.015	Adenylate kinase isoenzyme 6 homolog
Dcitr10g01270.1.1	2.489	0.022	4-aminobutyrate aminotransferase
Dcitr03g01700.1.1	2.488	0.003	72 kDa type IV collagenase
Dcitr05g09020.1.1	2.478	0.031	DNA-directed RNA polymerases I/II/III subunit
Dcitr00g10110.1.1	2.449	0.004	zinc
Dcitr11g01700.1.1	2.380	0.028	trypsin I-P1-like, partial
Dcitr05g10240.1.1	2.369	0.001	Phosphoenolpyruvate carboxykinase
Dcitr10g07040.1.1	2.343	0.018	Heat shock protein 70
Dcitr06g09500.1.1	2.316	0.000	Neurotrypsin
Dcitr04g14800.1.1	2.294	0.005	Major facilitator, sugar transporter-like, Major facilitator superfamily domain protein
Dcitr01g12250.1.1	2.277	0.044	Calmodulin
Dcitr01g22110.1.1	2.263	0.026	Mite allergen Der p 7
Dcitr01g13510.1.1	2.247	0.035	WD repeat-containing protein 19
Dcitr01g03180.1.1	2.240	0.000	Odorant binding protein
Dcitr04g17030.1.1	2.240	0.049	Histone H2B
Dcitr01g09440.1.1	2.212	0.040	WD40 repeat protein
Dcitr01g16420.1.1	2.143	0.040	Carboxypeptidase B
Dcitr05g13810.1.1	2.137	0.012	Dynein regulatory complex subunit 7
Dcitr01g06830.1.1	2.111	0.013	Protein with WD-40 repeat domain
Dcitr03g19110.1.1	2.075	0.009	Unknown protein
Dcitr03g11520.1.1	2.073	0.000	Cystatin
Dcitr08g10620.1.1	-2.030	0.007	Syntaxin-41
Dcitr08g09060.1.1	-2.059	0.014	Pleckstrin homology domain-containing family M member 2
Dcitr01g18410.1.1	-2.133	0.011	Cullin-associated NEDD8-dissociated protein 1
Dcitr03g13820.1.1	-2.185	0.000	PRELI/MSF1 domain-containing protein
Dcitr07g07760.1.1	-2.216	0.026	Doubletime
Dcitr03g15900.1.1	-2.257	0.006	Serine/threonine-protein phosphatase 4 regulatory subunit 3
Dcitr06g05800.1.1	-2.413	0.014	DNA-directed RNA polymerase III subunit rpc-3
Dcitr02g11330.1.4	-2.534	0.000	DnaJ subfamily B member 2
Dcitr10g09050.1.2	-2.563	0.001	Acyl carrier protein
Dcitr10g01500.1.1	-2.568	0.032	Zinc finger MYM-type protein 1
Dcitr03g19770.1.1	-2.569	0.033	Unknown protein
Dcitr03g06820.1.1	-2.581	0.049	Suppressor of fused
Dcitr08g02060.1.1	-2.640	0.000	RING/U-box superfamily protein
Dcitr03g07620.1.1	-2.729	0.008	DNA repair/transcription protein mms19

Dcitr13g03440.1.1	-2.772	0.000	Long chain acyl-coa synthetase
Dcitr01g10860.1.1	-2.826	0.037	Ubiquinol-cytochrome c reductase complex chaperone CBP3
Dcitr05g06210.1.2	-2.847	0.000	Unknown protein
Dcitr11g01280.1.1	-2.851	0.000	Zinc finger protein 879
Dcitr04g15470.1.1	-2.911	0.000	Tensin
Dcitr10g08880.1.1	-2.981	0.002	cold shock domain protein 1 Chr4:17043443-17044342 REVERSE LENGTH%3D299 201606
Dcitr11g09700.1.1	-3.057	0.000	Collagen alpha 2(I) chain
Dcitr05g05120.1.1	-3.347	0.033	Unknown protein
Dcitr00g01590.1.1	-3.878	0.015	Collagen
Dcitr01g05780.1.1	-3.886	0.015	Thymidylate kinase
Dcitr07g08900.1.1	-4.005	0.004	Phosphomannomutase
Dcitr08g01460.1.1	-4.024	0.002	HSP20-like chaperone
Dcitr06g14110.1.1	-4.026	0.010	Transmembrane protein 165
Dcitr03g13500.1.1	-4.245	0.028	MAM domain-containing protein 2
Dcitr00g06180.1.2	-4.265	0.000	Broad-complex
Dcitr08g12680.1.2	-4.271	0.005	Mediator of RNA polymerase II transcription subunit 14
Dcitr02g15740.1.4	-4.292	0.006	Rap guanine nucleotide exchange factor 4
Dcitr03g18670.1.1	-4.383	0.004	CBS domain-containing protein / transporter associated domain-containing protein
Dcitr01g04810.1.1	-4.393	0.004	26S proteasome regulatory subunit
Dcitr04g12470.1.1	-4.481	0.004	39S ribosomal protein L10, mitochondrial
Dcitr11g06930.1.1	-4.505	0.000	Thymidylate kinase
Dcitr01g11490.1.1	-4.512	0.000	28S ribosomal protein S24, mitochondrial
Dcitr05g17080.1.1	-4.516	0.010	Long chain base biosynthesis protein 1-like
Dcitr04g11590.1.1	-4.522	0.014	Suppressor of hairless protein
Dcitr05g15560.1.1	-4.525	0.003	Proton-coupled amino acid transporter 4
Dcitr10g08700.1.1	-4.613	0.012	CAP-Gly domain-containing linker protein 1
Dcitr01g19850.1.1	-4.662	0.009	Larval cuticle protein 16/17
Dcitr05g03690.1.1	-4.683	0.005	histidine-rich glycoprotein
Dcitr10g08260.1.1	-4.784	0.003	Sin3 histone deacetylase corepressor complex component SDS3
Dcitr03g08640.1.1	-4.842	0.010	GM25696
Dcitr06g04860.1.1	-4.944	0.001	LOW QUALITY PROTEIN: myb-like protein P
Dcitr03g12480.1.4	-5.217	0.001	S-adenosyl-L-methionine-dependent methyltransferases superfamily protein Chr1:5687994-5690395 FORWARD LENGTH%3D382 201606
Dcitr11g05020.1.2	-5.346	0.001	Trafficking protein particle complex subunit
Dcitr02g13040.1.1	-5.377	0.000	CD5 antigen-like
Dcitr06g14230.1.1	-5.452	0.000	Forkhead box protein O
Dcitr00g08930.1.1	-5.464	0.001	Serine
Dcitr00g04570.1.4	-5.549	0.000	Zinc
Dcitr02g14470.1.1	-5.729	0.000	Cell adhesion molecule 3
Dcitr05g04450.1.1	-6.012	0.000	Nuclear transport factor 2
Dcitr11g01720.1.2	-6.054	0.000	Alpha-tocopherol transfer protein-like
Dcitr01g10250.1.1	-6.174	0.000	Zinc finger protein 84
Dcitr04g09190.1.1	-6.248	0.000	jerky protein homolog-like, partial
Dcitr02g07060.1.1	-6.340	0.000	Dorsal-ventral patterning protein Sog
Dcitr10g01320.1.3	-6.570	0.000	Unknown protein
Dcitr10g09890.1.1	-6.828	0.000	Thioredoxin-mitochondrial 1
Dcitr04g15090.1.1	-6.913	0.000	NADH-quinone oxidoreductase subunit B
Dcitr00g04130.1.1	-6.971	0.000	Origin
Dcitr02g07360.1.4	-7.227	0.000	Adenylate kinase

Dcitr06g01400.1.1	-7.544	0.000	Unknown protein
Dcitr01g13900.1.1	-8.191	0.000	Mitochondrial pyruvate carrier
Dcitr01g14790.1.1	-8.641	0.000	Calreticulin
Dcitr02g11330.1.2	-8.972	0.000	DnaJ subfamily B member 2
Dcitr04g16360.1.1	-9.165	0.000	EH domain-binding protein 1

Table S4: All transcripts from the salivary gland dataset that have differential expression log2FoldChange>|2| and adjusted p-value<0.05. Sorted by Log2FoldChange.

Transcript_ID	L2FC	padj	Annotation
Dcitr04g09440.1.1	11.361	0.000	NADH-ubiquinone oxidoreductase
Dcitr04g13690.1.1	10.523	0.000	40S ribosomal protein S28
Dcitr10g10180.1.1	10.129	0.000	40S ribosomal protein S15a
Dcitr08g07040.1.1	9.306	0.000	Phosphate acyltransferase
Dcitr01g05060.1.1	8.412	0.000	Acyl-CoA thioesterase
Dcitr12g05490.1.1	8.155	0.000	Gamma-glutamylcyclotransferase
Dcitr01g09640.1.1	8.048	0.000	Alpha-tocopherol transfer protein-like protein
Dcitr10g02950.1.1	7.829	0.000	Signal peptidase I
Dcitr13g03480.1.1	7.406	0.000	Ubiquitin conjugating enzyme
Dcitr03g19180.1.1	7.209	0.000	CAAX prenyl protease 1 (Peptidase family M48)
Dcitr03g16830.1.3	7.171	0.000	basic salivary proline-rich protein 3-like
Dcitr05g11190.1.1	7.048	0.000	Methylthioribulose-1-phosphate dehydratase
Dcitr11g07970.1.2	6.765	0.000	Calcium-binding EF-hand
Dcitr13g07090.1.3	6.693	0.000	Farnesyl pyrophosphate synthase
Dcitr05g07830.1.1	6.622	0.000	LIM homeobox transcription factor 1-beta
Dcitr07g02170.1.1	6.442	0.002	Mitochondrial carrier
Dcitr01g11050.1.2	6.436	0.000	Soluble calcium-activated nucleotidase 1
Dcitr04g16010.1.1	6.260	0.002	Unknown protein
Dcitr11g09190.1.4	6.220	0.000	Geranylgeranyl transferase type-1 subunit beta
Dcitr13g03250.1.1	6.199	0.002	Leucine--tRNA ligase
Dcitr13g04800.1.1	6.048	0.000	Tethering factor for nuclear proteasome STS1
Dcitr08g04630.1.1	5.991	0.000	UDP-N-acetylglucosamine pyrophosphorylase 1
Dcitr02g12920.1.2	5.959	0.007	ABC transporter C family
Dcitr07g06610.1.1	5.910	0.000	Meteorin-like protein
Dcitr03g05340.1.1	5.892	0.001	tctex1 domain-containing protein 1-like
Dcitr05g14340.1.1	5.883	0.002	Flavin-containing monooxygenase
Dcitr03g15750.1.1	5.861	0.011	Serine protease
Dcitr01g11640.1.1	5.787	0.021	Serine/threonine-protein kinase 16
Dcitr13g01840.1.1	5.782	0.002	Adenylate kinase isoenzyme 6 homolog
Dcitr03g04260.1.1	5.774	0.000	Ribosomal protein
Dcitr06g03150.1.1	5.737	0.004	Phospholipid-transporting ATPase
Dcitr06g05570.1.1	5.727	0.002	NADH dehydrogenase [ubiquinone] 1 α subcomplex subunit 7
Dcitr01g21740.1.1	5.624	0.021	Thioredoxin domain-containing protein 17
Dcitr03g04400.1.1	5.535	0.001	60S ribosomal protein L4
Dcitr01g04160.1.1	5.462	0.000	Unknown protein
Dcitr02g18560.1.1	5.450	0.007	Unknown protein
Dcitr11g05250.1.1	5.428	0.003	Elongation factor 2
Dcitr05g04310.1.1	5.399	0.003	AGAP013432-PA
Dcitr06g05040.1.1	5.372	0.012	Unknown protein
Dcitr12g02110.1.1	5.359	0.029	Very-long-chain 3-oxoacyl-CoA reductase 1
Dcitr00g13000.1.1	5.234	0.006	ABC
Dcitr02g16560.1.1	5.193	0.015	TBC1 domain family member 31-like isoform X2
Dcitr10g09840.1.1	5.168	0.043	testis specific serine/threonine protein kinase 3
Dcitr01g13970.1.1	5.168	0.005	Elongation factor 1-beta
Dcitr01g10480.1.1	5.140	0.026	33 kDa inner dynein arm light chain, axonemal
Dcitr04g11380.1.1	5.059	0.040	Unknown protein

Dcitr03g02880.1.1	5.055	0.045	spermatogenesis-associated protein 7-like isoform X1
Dcitr01g05220.1.1	5.048	0.009	UPF0691 protein C9orf116
Dcitr03g04980.1.1	5.042	0.024	zinc finger protein 436-like isoform X1
Dcitr01g20800.1.1	5.006	0.029	Pleckstrin homology domain-containing family M member 1
Dcitr09g07010.1.1	4.986	0.011	Alpha-Crystallin
Dcitr10g02810.1.1	4.963	0.038	Unknown protein
Dcitr00g14590.1.1	4.908	0.021	Transposon
Dcitr11g08720.1.1	4.881	0.045	Protein yellow
Dcitr01g15810.1.1	4.856	0.025	RNA helicase
Dcitr05g09660.1.1	4.854	0.030	Unknown protein
Dcitr04g10840.1.1	4.841	0.015	Protein FAM210A
Dcitr13g01100.1.1	4.811	0.001	Snurportin-1
Dcitr03g11970.1.1	4.763	0.030	DNA repair RAD51-like protein
Dcitr10g07900.1.1	4.757	0.022	Macrophage erythroblast attacher
Dcitr08g10720.1.1	4.697	0.038	Homeobox protein Nkx-2.2a
Dcitr02g04500.1.1	4.694	0.030	Phosphoribosylamine--glycine ligase
Dcitr06g04060.1.1	4.693	0.031	Ubiquitin-ligase E3
Dcitr01g07660.1.1	4.670	0.028	Unknown protein
Dcitr05g14800.1.1	4.595	0.010	Elongation factor 4
Dcitr10g10090.1.1	4.579	0.038	Unknown protein
Dcitr01g16880.1.1	4.567	0.042	WD40 repeat
Dcitr02g04530.1.1	4.464	0.006	Biogenesis of lysosome-related organelles complex 1 subunit 1
Dcitr03g13410.1.1	4.445	0.021	Polyadenylate-binding protein-interacting protein 2B
Dcitr01g07920.1.1	4.398	0.024	Unknown protein
Dcitr06g11690.1.1	4.324	0.002	Peroxiredoxin
Dcitr03g06640.1.1	4.312	0.028	Zinc finger CCHC domain-containing protein 4
Dcitr02g13860.1.1	4.277	0.020	RNA-directed DNA polymerase from mobile element jockey
Dcitr08g03350.1.1	4.260	0.030	Ecdysone-induced protein 74EF isoform B
Dcitr04g11610.1.2	4.038	0.027	MFS-type transporter C6orf192
Dcitr07g08050.1.1	3.944	0.046	Transmembrane emp24 domain-containing protein 7
Dcitr10g06970.1.2	3.701	0.006	U3 small nucleolar RNA-associated protein 6
Dcitr03g15730.1.1	3.646	0.010	CLIPB - Serine Protease 1
Dcitr07g02410.1.1	3.265	0.032	Chloride channel protein
Dcitr05g16750.1.1	3.236	0.006	Pro-Pol polyprotein
Dcitr04g06660.1.1	3.182	0.035	NADPH-dependent diflavin oxidoreductase 1
Dcitr05g16000.1.2	3.180	0.049	Fatty-acid amide hydrolase 2
Dcitr13g04790.1.1	2.952	0.020	Lipid phosphate phosphatase
Dcitr02g08670.1.1	2.904	0.019	Lamin-B1
Dcitr04g09870.1.1	2.678	0.008	RNA polymerase II-associated protein 1
Dcitr10g07230.1.2	2.608	0.009	Ankyrin repeat and MYND domain-containing protein 2
Dcitr07g02010.1.1	2.587	0.008	Guanine deaminase
Dcitr07g02260.1.1	2.536	0.006	Nuclear autoantigenic sperm protein
Dcitr12g07070.1.1	2.530	0.013	oxidative stress-responsive serine-rich protein 1
Dcitr12g05190.1.1	2.256	0.015	ATP synthase gamma chain
Dcitr04g11810.1.1	2.218	0.032	Transposon Ty3-I Gag-Pol polyprotein
Dcitr04g02090.1.1	-2.522	0.025	Unknown protein
Dcitr07g03910.1.1	-2.647	0.001	Unknown protein
Dcitr11g01500.1.1	-3.290	0.000	RNA-directed DNA polymerase from mobile element jockey
Dcitr10g10160.1.1	-4.359	0.024	Protein sidekick
Dcitr03g13400.1.2	-4.409	0.000	UDP-glucuronosyltransferase
Dcitr09g07790.1.1	-4.641	0.045	conserved

Dcitr01g09640.1.2	-5.198	0.037	Alpha-tocopherol transfer protein-like protein
Dcitr02g02390.1.2	-5.323	0.010	E3 SUMO-protein ligase PIAS3
Dcitr08g05310.1.1	-5.772	0.043	5-aminolevulinate synthase
Dcitr02g02120.1.1	-5.908	0.000	Unknown protein
Dcitr05g07530.1.1	-6.262	0.017	U2 small nuclear ribonucleoprotein B
Dcitr06g05250.1.1	-7.423	0.000	Zinc finger protein 417
Dcitr03g04630.1.2	-7.934	0.000	NAD(P)-linked oxidoreductase superfamily protein
Dcitr01g22340.1.1	-8.455	0.000	Unknown protein

Table S5: All transcripts from the bacteriome dataset that have differential expression log2FoldChange>|2| and adjusted p-value<0.05. Sorted by Log2FoldChange.

Transcript_ID	L2FC	padj	Annotation
Dcitr01g16790.1.1	8.432	0.000	Cationic amino acid transporter
Dcitr04g03620.1.1	7.487	0.000	Methyltransferase family protein
Dcitr01g21740.1.1	7.335	0.000	Thioredoxin domain-containing protein 17
Dcitr06g12720.1.2	6.793	0.001	Sorbin and SH3 domain-containing protein 2
Dcitr01g12970.1.1	6.466	0.004	Zinc-binding dehydrogenase
Dcitr10g11050.1.1	6.295	0.001	Unknown protein
Dcitr01g09990.1.1	6.114	0.006	Androgen-induced gene 1 protein
Dcitr08g01750.1.2	5.977	0.007	Blood vessel epicardial substance
Dcitr07g08590.1.1	5.592	0.028	Major facilitator transporter
Dcitr04g12240.1.1	5.483	0.006	Lactoylglutathione lyase
Dcitr06g04730.1.1	5.482	0.025	Phthiotriol/phenolphthiotriol dimycocerosates methyltransferase
Dcitr02g04200.1.2	5.314	0.005	Dystroglycan
Dcitr11g06040.1.1	5.140	0.011	GATA zinc finger domain-containing protein 10-like, partial
Dcitr13g03670.1.1	5.090	0.012	Gag-Pro-Pol polyprotein
Dcitr00g02740.1.1	4.974	0.020	RING/U-box
Dcitr03g07550.1.1	4.541	0.004	Delta-1-pyrroline-5-carboxylate synthase
Dcitr04g04380.1.1	4.539	0.022	Piezo-type mechanosensitive ion channel component 1
Dcitr01g05990.1.1	4.446	0.002	Acid phosphatase-like protein 2
Dcitr00g02000.1.1	4.360	0.025	Unknown
Dcitr10g03040.1.3	4.353	0.000	SH3 domain-containing protein Dlish
Dcitr04g01760.1.1	4.304	0.026	LOW QUALITY PROTEIN: uncharacterized protein LOC112591404
Dcitr00g11870.1.1	4.210	0.007	Elongation
Dcitr01g20610.1.1	4.122	0.011	F-actin-capping protein subunit alpha
Dcitr04g01700.1.1	4.066	0.005	Erg28-domain containing protein
Dcitr06g09230.1.1	4.053	0.005	COP9 signalosome complex subunit 7a
Dcitr03g07170.1.1	4.034	0.008	60S ribosomal protein L26
Dcitr05g16270.1.1	3.932	0.027	early endosome antigen 1-like
Dcitr12g10310.1.1	3.875	0.050	Unknown protein
Dcitr08g11110.1.1	3.744	0.007	Agrin
Dcitr03g19430.1.1	3.733	0.021	Hexokinase
Dcitr01g18830.1.1	3.609	0.024	sphingomyelin phosphodiesterase 4-like
Dcitr02g02510.1.1	3.555	0.008	Phosphoenolpyruvate synthase
Dcitr02g19280.1.1	3.319	0.019	Poly [ADP-ribose] polymerase
Dcitr00g12300.1.1	3.299	0.020	Protein
Dcitr01g18190.1.1	3.254	0.032	60S ribosomal protein L37a
Dcitr04g05130.1.1	3.212	0.039	Unknown protein
Dcitr13g06110.1.1	3.158	0.040	Unknown protein
Dcitr05g10980.1.1	3.122	0.016	RING finger and SPRY domain-containing protein 1
Dcitr03g08980.1.1	3.075	0.000	Unknown protein
Dcitr07g01070.1.1	3.071	0.002	L-allo-threonine aldolase
Dcitr11g01560.1.1	3.070	0.030	Cation-chloride cotransporter 1
Dcitr02g19260.1.1	2.937	0.014	Ribosomal protein L23
Dcitr07g01080.1.2	2.899	0.025	Myosin heavy chain, non-muscle
Dcitr03g19060.1.1	2.863	0.000	protein phosphatase 1 regulatory subunit 21-like
Dcitr12g05400.1.1	2.853	0.024	5'-nucleotidase

Dcitr03g08990.1.1	2.549	0.000	Unknown protein
Dcitr01g01470.1.1	2.540	0.006	Poly(A) RNA polymerase, mitochondrial
Dcitr06g08650.1.1	2.535	0.000	Lipase
Dcitr05g01800.1.1	2.473	0.000	PiggyBac transposable element-derived protein 4
Dcitr11g09370.1.1	2.456	0.038	Kanadaptin
Dcitr04g11810.1.1	2.446	0.000	Transposon Ty3-I Gag-Pol polyprotein
Dcitr04g09800.1.1	2.413	0.036	Aldose 1-epimerase
Dcitr04g01290.1.1	2.392	0.000	Mortality factor 4-like protein 1
Dcitr05g14050.1.1	2.344	0.011	Cytochrome P450 305E1
Dcitr13g05490.1.1	2.269	0.000	Aldehyde dehydrogenase
Dcitr04g01240.1.1	2.268	0.028	Mitotic checkpoint serine/threonine-protein kinase BUB1
Dcitr05g07320.1.1	2.260	0.030	N-acetylaspartate synthetase
Dcitr05g06560.1.1	2.216	0.004	LETM1 domain-containing protein 1
Dcitr02g04730.1.1	2.207	0.000	Spondin-1
Dcitr07g01110.1.1	2.172	0.000	Myosin heavy chain, non-muscle
Dcitr04g11820.1.1	2.162	0.000	Transposon Ty3-I Gag-Pol polyprotein
Dcitr05g17180.1.1	2.136	0.013	DnaJ subfamily C member 22
Dcitr03g19600.1.1	2.106	0.007	Protein arginine N-methyltransferase
Dcitr03g09890.1.1	2.072	0.035	Unknown protein
Dcitr04g11220.1.2	2.069	0.003	pre-mRNA-splicing factor CWC22 homolog isoform X2
Dcitr05g15210.1.1	2.058	0.035	serine/threonine-protein phosphatase 6 regulatory subunit 3-A
Dcitr11g01990.1.1	2.052	0.001	Pro-Pol polyprotein
Dcitr09g07400.1.1	2.039	0.036	Ribonuclease
Dcitr00g12410.1.1	2.035	0.047	E3
Dcitr13g01090.1.1	2.013	0.000	Myb
Dcitr01g09300.1.1	-2.059	0.004	Unknown protein
Dcitr02g18210.1.1	-2.136	0.037	ABC transporter G family
Dcitr03g02070.1.1	-2.147	0.022	Fibroblast growth factor 17
Dcitr03g18480.1.1	-2.176	0.038	Histone acetyltransferase type B catalytic subunit
Dcitr11g03410.1.1	-2.302	0.018	Unconventional myosin-IXa
Dcitr12g03860.1.1	-2.316	0.022	organic solute transporter ostalpha protein (DUF300) Chr5:9292436-9294407 FORWARD LENGTH%3D422 201606
Dcitr07g08190.1.1	-2.318	0.009	Peroxisomal N(1)-acetyl-spermine/spermidine oxidase
Dcitr11g06580.1.1	-2.341	0.024	CLUMA_CG018496, isoform A
Dcitr07g06540.1.1	-2.361	0.000	4-hydroxy-tetrahydrodipicolinate synthase
Dcitr10g02220.1.1	-2.381	0.014	Cytochrome c oxidase subunit
Dcitr04g06980.1.1	-2.412	0.000	E3 ubiquitin-protein ligase E3D
Dcitr00g06360.1.1	-2.513	0.040	Sorting
Dcitr08g10240.1.1	-2.520	0.022	Filamin-B
Dcitr00g10370.1.1	-2.576	0.006	Sphingomyelin
Dcitr03g07570.1.1	-2.605	0.037	Delta-1-pyrroline-5-carboxylate synthase
Dcitr02g04720.1.1	-2.660	0.000	DNA helicase
Dcitr05g15920.1.1	-2.794	0.004	Major facilitator superfamily domain-containing protein 6
Dcitr02g10750.1.1	-2.841	0.016	minichromosome maintenance domain-containing protein 2 isoform X1
Dcitr07g03820.1.1	-2.848	0.006	Aminopeptidase
Dcitr01g17200.1.1	-2.885	0.012	Prolyl oligopeptidase family protein
Dcitr03g21220.1.1	-2.934	0.002	hemicentin-1-like
Dcitr01g05380.1.1	-3.088	0.035	transcription factor A, mitochondrial
Dcitr07g09000.1.1	-3.272	0.038	Alkaline nuclease
Dcitr00g06180.1.1	-3.618	0.041	Broad-complex

Dcitr01g06690.1.1	-3.653	0.003	Elongation factor 4
Dcitr04g07450.1.1	-3.702	0.008	retinaldehyde-binding protein 1-like
Dcitr03g09640.1.1	-3.744	0.007	Unknown protein
Dcitr06g06000.1.1	-3.760	0.000	Ankyrin repeat family protein
Dcitr03g01060.1.1	-3.764	0.005	L-lactate dehydrogenase
Dcitr00g09080.1.1	-3.788	0.007	Krueppel-like
Dcitr09g04720.1.2	-3.967	0.008	Vacuolar
Dcitr12g08510.1.4	-3.982	0.000	Bax inhibitor 1-related
Dcitr08g10290.1.1	-4.023	0.047	HEAT repeat-containing protein 5B
Dcitr04g11300.1.1	-4.444	0.044	Pre-mRNA-splicing factor CWC22
Dcitr07g06450.1.1	-4.566	0.024	2-oxoglutarate (2OG) and Fe(II)-dependent oxygenase superfamily protein
Dcitr03g03100.1.1	-4.640	0.019	regulator of G-protein signaling loco-like
Dcitr04g07640.1.1	-4.648	0.003	Protein SYS1 homolog
Dcitr06g14870.1.1	-4.749	0.027	Coiled-coil domain-containing protein 149
Dcitr01g22730.1.1	-4.786	0.000	RING/U-box superfamily protein Chr3:5102210-5104082 REVERSE LENGTH%3D249 201606
Dcitr10g04230.1.1	-4.940	0.020	CAP-Gly domain-containing linker protein 1
Dcitr03g19580.1.1	-5.409	0.050	Delta-aminolevulinic acid dehydratase
Dcitr06g03080.1.1	-5.828	0.000	Unknown protein
Dcitr10g04540.1.1	-6.068	0.024	NADPH--cytochrome P450 reductase

Table S6: All transcripts from the head dataset that have differential expression log2FoldChange>|2| and adjusted p-value<0.05. Sorted by Log2FoldChange.

HEAD	padj	L2FC	Annotation
Dcitr05g09170.1.1	0.039	-5.333	ATP synthase delta subunit
Dcitr05g06500.1.1	0.032	-4.263	Unknown protein
Dcitr02g01080.1.1	0.027	-4.095	Vigilin
Dcitr01g21980.1.1	0.027	-3.426	RNA-directed DNA polymerase from mobile element jockey
Dcitr02g09580.1.1	0.032	3.415	intracellular protein transport protein USO1
Dcitr04g10550.1.1	0.001	5.276	neuromodulin
Dcitr07g08870.1.1	0.024	5.289	Rho GTPase-activating protein 17
Dcitr10g06500.1.1	0.005	5.562	Glucocorticoid-induced transcript 1 protein
Dcitr05g04030.1.1	0.043	5.615	Tumor protein p53-inducible nuclear protein 1
Dcitr08g09760.1.3	0.014	5.633	Insulin-like growth factor 2 mRNA-binding protein 3

Table S7: Data used to generate Figure 3, including annotations, p-values and Log2FoldChange values for each transcript listed.

Endocytosis

Gene_ID	Midgut	padj	Bacteriome	padj	Salivary Gland	padj
Dcitr01g20610.1.1	0.04	0.99	4.12	0.01	-2.79	0.14
Dcitr02g07360.1.4	-7.23	0.00	-0.21	0.99	NA	NA
Dcitr05g12140.1.1	3.75	0.04	-0.51	0.79	0.56	0.97
Dcitr05g16270.1.1	0.88	0.58	3.93	0.03	NA	NA
Dcitr07g01080.1.2	-0.77	NA	2.90	0.03	4.62	0.15
Dcitr07g01110.1.1	-2.44	0.49	2.17	0.00	2.01	0.65
Dcitr07g08110.1.1	4.32	0.03	-0.69	NA	NA	NA
Dcitr09g04720.1.2	-0.44	NA	-3.97	0.01	0.27	0.98
Dcitr11g04950.1.2	3.97	0.00	0.12	1.00	3.25	0.20
Dcitr12g03760.1.1	3.62	0.02	-1.05	0.96	1.29	0.95
Dcitr12g03760.1.1	3.62	0.01	-1.05	0.58	1.29	0.00
Dcitr12g07070.1.1	NA	0.00	0.88	0.87	2.53	0.99
Dcitr13g01820.1.1	3.60	0.02	-0.57	0.96	-1.17	0.95
Dcitr13g01840.1.1	2.51	NA	2.82	0.19	5.78	0.01
Dcitr13g01890.1.1	2.96	0.02	-1.05	0.04	0.52	0.88

Ubiquination

Gene_ID	Midgut	padj	Bacteriome	padj	Salivary Gland	padj
Dcitr00g12410.1.1	0.60	0.84	2.04	0.05	0.64	0.98
Dcitr01g04810.1.1	-4.39	0.00	-1.11	0.00	-0.71	0.98
Dcitr01g10860.1.1	-2.83	0.04	0.13	0.98	0.65	0.98
Dcitr01g22250.1.1	4.76	0.01	2.31	NA	0.71	0.98
Dcitr02g02390.1.2	0.05	1.00	0.18	1.00	-5.32	0.01
Dcitr02g09710.1.1	4.57	0.00	-0.53	0.99	0.42	0.98
Dcitr04g06980.1.1	-0.01	1.00	-2.41	0.00	0.57	0.98
Dcitr04g09440.1.1	-1.94	0.54	0.08	0.99	11.36	0.00
Dcitr06g04060.1.1	-1.52	0.52	2.86	0.57	4.69	0.03
Dcitr10g09310.1.1	3.43	0.05	-0.91	0.86	2.08	0.49
Dcitr13g03480.1.1	0.24	0.93	-0.23	0.78	7.41	0.00
Dcitr13g04800.1.1	0.29	0.95	-1.93	NA	6.05	0.00

Immunity

Gene_ID	Midgut	padj	Bacteriome	padj	Salivary Gland	padj
Dcitr01g11640.1.1	-0.07	0.99	-3.66	NA	-6.26	0.02
Dcitr02g03230.1.1	4.03	0.00	0.18	0.99	1.28	0.62

Dcitr02g04530.1.1	0.32	0.91	-0.65	0.16	3.70	0.01
Dcitr02g04730.1.1	2.53	0.38	2.21	0.00	10.13	0.00
Dcitr03g07590.1.1	3.51	0.00	-0.80	0.95	5.79	0.02
Dcitr03g15730.1.1	2.66	0.15	-1.75	0.85	0.15	0.99
Dcitr03g15750.1.1	2.18	0.41	-1.95	0.81	4.46	0.01
Dcitr03g15760.1.1	3.02	0.01	-0.90	0.94	2.29	0.36
Dcitr03g15900.1.1	-2.26	0.01	-0.47	0.72	1.21	0.95
Dcitr07g03820.1.1	-1.58	0.73	-2.85	0.01	3.65	0.01
Dcitr07g08110.1.1	4.32	0.03	-0.69	NA	5.86	0.01
Dcitr10g09840.1.1	1.44	0.41	-0.61	0.98	-0.14	0.99
Dcitr05g07530.1.1	NA	NA	-0.14	0.99	0.06	1.00
Dcitr09g07400.1.1	-0.31	0.98	2.04	0.04	0.67	0.98
Dcitr10g06970.1.2	-0.71	0.94	0.14	0.99	NA	NA
Dcitr10g10180.1.1	0.80	0.65	-0.66	0.45	5.17	0.04

Ribosomal

Gene_ID	Midgut	padj	Bacteriome	padj	Salivary Gland	padj
Dcitr01g11490.1.1	-4.51	0.00	-0.13	0.96	NA	NA
Dcitr01g18190.1.1	-2.82	0.37	3.25	0.03	0.22	0.99
Dcitr02g04500.1.1	-0.52	0.94	NA	NA	4.69	0.03
Dcitr02g14920.1.1	3.40	0.00	-1.09	0.40	-0.68	0.98
Dcitr02g19260.1.1	-0.25	NA	2.94	0.01	2.25	0.52
Dcitr02g19280.1.1	-0.42	0.62	3.32	0.02	0.33	0.99
Dcitr03g04260.1.1	-2.60	NA	0.30	0.99	5.77	0.00
Dcitr03g04400.1.1	2.75	NA	-0.27	0.99	5.53	0.00
Dcitr03g07170.1.1	1.79	0.60	4.03	0.01	0.65	0.98
Dcitr04g05810.1.1	6.17	0.00	0.25	0.98	-2.62	0.77
Dcitr04g12470.1.1	-4.48	0.00	-0.70	0.89	-0.83	0.88
Dcitr04g13690.1.1	NA	NA	-0.09	1.00	10.52	0.00

Table S8: All transcripts from the salivary gland dataset that have predicted signal sequences. The four in **bold** text had predicted transmembrane helices.

Transcript ID	padj	L2FC	Annotation	Mw (kD)	Cleavage site	AA site
Dcitr05g04310.1.1	0.003	5.399	AGAP013432-PA	10.12	14-15	ALC-DQ
Dcitr03g16830.1.3	0.000	7.171	basic salivary proline-rich protein 3-like	14.17	25-26	VIG-QS
Dcitr03g15730.1.1	0.010	3.646	CLIPB - Serine Protease 1	35.39	22-23	GLA-YS
**Dcitr09g07790.1.1**	**0.045**	**-4.641**	**conserved**	**57.12**	**20-21**	**ISA-ES**
**Dcitr13g04790.1.1**	**0.020**	**2.952**	**Lipid phosphate phosphatase**	**34.06**	**21-22**	**SKQ-GF**
Dcitr07g06610.1.1	0.000	5.910	Meteorin-like protein	36.49	28-29	ITG-LV
Dcitr03g15750.1.1	0.011	5.861	Serine protease	35.19	22-23	GLA-YS
**Dcitr07g08050.1.1**	**0.046**	**3.944**	**Transmembrane emp24 domain-containing protein 7**	**25.58**	**28-29**	**VQA-VE**
**Dcitr03g13400.1.2**	**0.000**	**-4.409**	**UDP-glucuronosyltransferase**	**57.55**	**19-20**	**AQG-AN**
Dcitr01g04160.1.1	0.000	5.462	Unknown protein	29.65	19-20	TQS-QL
Dcitr05g09660.1.1	0.030	4.854	Unknown protein	6.87	21-22	TLA-SD
Dcitr01g22340.1.1	0.000	-8.455	Unknown protein	75.36	20-21	TLC-GV

Table S9: All piggyBac-related genes currently annotated in the Diaci_v3.0 genome. The gene identified in our transcript analysis is in **bold**.

Gene_ID	Annotation
Dcitr02g01360.1.1	PiggyBac transposable element-derived protein 4
Dcitr03g11500.1.1	PiggyBac transposable element-derived protein 2
**Dcitr05g01800.1.1**	**PiggyBac transposable element-derived protein 4**
Dcitr05g01980.1.1	PiggyBac transposable element-derived protein 4
Dcitr05g02000.1.1	PiggyBac transposable element-derived protein 4
Dcitr08g12690.1.1	PiggyBac transposable element-derived protein 2
Dcitr09g02860.1.1	PiggyBac
Dcitr09g03340.1.1	PiggyBac
Dcitr10g03650.1.1	piggyBac transposable element-derived protein 3-like
Dcitr10g10330.1.1	PiggyBac transposable element-derived protein 2
Dcitr11g09800.1.1	PiggyBac transposable element-derived protein 4
