# Supplementary material for: Lessons learned about the biology and genomics of Diaphorina citri infection with “Candidatus Liberibacter asiaticus” by integrating new and archived organ-specific transcriptome data
Source: Gigascience. 2022 Apr 28;11:giac035. doi: 10.1093/gigascience/giac035 (PMC9049105; doi:10.1093/gigascience/giac035)
Supplement: giac035_Response_to_Reviewer_Comments_Original_Submission [file giac035_response_to_reviewer_comments_original_submission.pdf]

Author's Response To Reviewer Comments

Close

Nov. 16, 2021

Dear Dr. Hongfang Zhang,
Thank you to you and the two reviewers for the helpful feedback on our manuscript. We are including a point-by-point response to the reviewers. The suggestions of the reviewers greatly strengthened the manuscript. Their comments were straightforward and we were able to address all of them. We appreciate your consideration of the revised version.

With best regards,
Michelle Heck

Reviewer #1: GIGA-D-21-00314
The manuscript {Lessons learned about the biology and genomics of Diaphorina citri infection with "Candidatus Liberibacter asiaticus" by integrating new and archived organ-specific transcriptome data} is well written and provide important insights in the interactions between 'Ca. L. asiaticus' and its insect vector, D. citri. I highly recommend the manuscript to be published after minor revision. My comments are indicated in the attached PDF file.

Mann et al:
Thank you for your kind comments about the manuscript. We have reviewed your edits in the PDF and have made most of the suggested minor revisions. We acknowledge that your suggestion to use "Ca. L. asiaticus" is important, however, our current use of CLas as an abbreviation is widely accepted and published in well-reviewed journals (see recent perspective article by Huang et al., 2020 for example). Thus, we have maintained our use of CLas to refer to the bacterium. As for other small edits (adding spaces, correcting characters and use of "-", and defining certain terms like "adjusted p-value"), we have implemented all your suggestions throughout the manuscript.
In response to adding the reference Yu and Killiny (2018) to the discussion of salivary proteins, we made a brief comparison of transcripts identified by this study, to those found in table 1 of this reference.
In the potential implications section, we agree the sentence was confusing and we improved it to read as, "We urge arthropod genome communities and funding bodies to continue to invest funds on genome improvement projects such as i5k [57] and Ag100Pest [58], and to emphasize reanalyzing previously generated data as it may yield higher confidence results after using an improved-quality genome."
We also re-organized the methods and data section as suggested by reviewer 1 in conjunction with a similar suggestion by reviewer 2. A table that clearly describes all the samples is now shown in the data section and any mis-placed methods have been moved into the methods section.

Reviewer #2: The present manuscript combines new and archived RNA-seq datasets toward the goal of producing tissue-specific transcriptome analyses of Diaphorina citri (ACP) exposed or unexposed to the CLas pathogen. Overall, I think the manuscript is a strong contribution to our study of ACP-CLas interactions because it uncovers some previously undescribed patterns of expression, clarifies where in the insects CLas is likely to accumulate and be detected, provides new salivary effectors candidates, and provides a rigorous analysis of the impacts of gene model annotation quality on the analysis of RNA-seq data. I have a few concerns that should be addressed in a revision.

1. The authors are clear that this paper is stitched together from multiple datasets that were not collected in identical ways. However, given this, it is difficult to find all of the information about how the colonies were reared, on what hosts, and under what conditions, all in one place. For instance, the methods starts by describing validation of infection rates and then discusses protocols for harvesting different tissues. However, the authors do not mention the rearing hosts for colonies used to generate each tissue type, the locations reared and any relevant differences, or citations if those data are already published (e.g., I believe the midgut samples were from the archival study, but this is not cited in the methods). It would be very helpful to have a table somewhere in the manuscript, ideally with the

methods or description of data, that summarizes the exact sources and rearing conditions for the animals dissected for tissues.

Mann et al:
Thank you for your thoughtful comments and recommendations. We agree that the methods section lacked pertinent information on sample collection. That information you were looking for was previously found in the Data Description section and has now been moved to its proper place in the Methods section. We have revised the Data Description section (see lines 156-174) to be more on-topic with the section's intended purpose. Additionally, we have added a table (Table 1) in the Data Description (see lines 176-178), summarizing the four datasets – specifically, how they differ from each other prior to sequencing.

2. While the inclusion of the earlier study dataset is useful, the present study does not necessarily disentangle the influence of study-level differences as drivers of variation among the data sets. This is because the present study does not repeat the midgut dissections to determine if the differences seen between the archival midgut dataset and the present-day datasets represent tissue-level differences in response to CLas. As they are very different tissues, we can reasonably expect that much of the variation is due to tissue specificity, but we don't really get the answer to the question of how much information we are losing/missing by combining disparate datasets into one analysis. This doesn't invalidate the study by any means, but I think the authors could be more explicit about this fact in the discussion, and possibly in the introduction.

Mann et al:
Thank you for this insightful comment. We rewrote parts of the Lessons Learned section entitled, “Improved genome quality did not determine the proportion of transcripts differentially expressed.” to clarify these points.

3. My other comments are minor and include the following.

Line 179-180 states that salivary gland replicates consisted of pools of 300 per replicate, but earlier in this section (line 167) it states replicates consisted of 150 per salivary gland replicate. Please clarify the number of glands pooled to constitute one replicate.

Mann et al:
Thank you for pointing out the discrepancy in number of pooled salivary glands, it has been corrected to 150 pooled adult salivary glands per biological replicate.

Main manuscript has CLas- in red and CLas+ in blue (Fig. 2), while supplementary data uses a different color scheme (CLas- in black and CLas+ in red). This is somewhat confusing. Consider harmonizing this across main MS and supplemental figures.

Mann et al:
We corrected the colors of the figure.

The discussion is somewhat hard to follow and jumps back and forth among topic areas. For instance, paragraph one guides the reader to a discussion of CLas reads in different tissues, but then abruptly switches to discussion of ACP-derived reads in the salivary glands and relation of genes expressed to ROS responses. The following paragraph loops back to the discussion of CLas reads in the tissues and focuses on the salivary glands. The paragraph after that jumps to discussion of the heads, then we again return to the salivary glands and discussion of evidence that CLas is replicating at high levels in this tissue. For the sake of clarity, you may want to structure this to collect the information about CLas reads in each tissue into distinct sections so the reader is not forced to go back and forth, then discuss the ACP gene expression patterns in the context of this knowledge about CLas replication activity and the different tissue sources.

Mann et al:
Thank you for your constructive feedback on the discussion. We agree and have reorganized the paragraphs to improve the logical flow from topic to topic.

The supplementary figures show a fair amount of variation among replicates. Notably, there are two

outliers in the salivary gland dataset that were stored in the -80 for longer than the others. Perhaps this warrants more attention in the discussion.

Mann et al:
Thank you, we agree clarifying our position and opinion on this point is important. See lines 454-459 where this has been discussed.

Line 682 - remove comma between "sequencing" and "raw data"

Mann et al:
Thank you, this has been corrected.

Close
