# Supplementary material for: Lessons learned about the biology and genomics of Diaphorina citri infection with “Candidatus Liberibacter asiaticus” by integrating new and archived organ-specific transcriptome data
Source: Gigascience. 2022 Apr 28;11:giac035. doi: 10.1093/gigascience/giac035 (PMC9049105; doi:10.1093/gigascience/giac035)
Supplement: giac035_Reviewer_2_Report_Original_Submission — Kerry Mauck -- 11/12/2021 Reviewed [file giac035_reviewer_2_report_original_submission.pdf]

Reviewer Report

Title: Lessons learned about the biology and genomics of Diaphorina citri infection with â€œCandidatus Liberibacter asiaticusâ€� by integrating new and archived organ-specific transcriptome data.

Version: Original Submission **Date:** 11/12/2021

Reviewer name: Kerry Mauck

Reviewer Comments to Author:

The present manuscript combines new and archived RNA-seq datasets toward the goal of producing tissue-specific transcriptome analyses of Diaphorina citri (ACP) exposed or unexposed to the CLas pathogen. Overall, I think the manuscript is a strong contribution to our study of ACP-CLas interactions because it uncovers some previously undescribed patterns of expression, clarifies where in the insects CLas is likely to accumulate and be detected, provides new salivary effectors candidates, and provides a rigorous analysis of the impacts of gene model annotation quality on the analysis of RNA-seq data. I have a few concerns that should be addressed in a revision.

1. The authors are clear that this paper is stitched together from multiple datasets that were not collected in identical ways. However, given this, it is difficult to find all of the information about how the colonies were reared, on what hosts, and under what conditions, all in one place. For instance, the methods starts by describing validation of infection rates and then discusses protocols for harvesting different tissues. However, the authors do not mention the rearing hosts for colonies used to generate each tissue type, the locations reared and any relevant differences, or citations if those data are already published (e.g., I believe the midgut samples were from the archival study, but this is not cited in the methods). It would be very helpful to have a table somewhere in the manuscript, ideally with the methods or description of data, that summarizes the exact sources and rearing conditions for the animals dissected for tissues.

2. While the inclusion of the earlier study dataset is useful, the present study does not necessarily disentangle the influence of study-level differences as drivers of variation among the data sets. This is because the present study does not repeat the midgut dissections to determine if the differences seen between the archival midgut dataset and the present-day datasets represent tissue-level differences in response to CLas. As they are very different tissues, we can reasonably expect that much of the variation is due to tissue specificity, but we don't really get the answer to the question of how much information we are losing/missing by combining disparate datasets into one analysis. This doesn't invalidate the study by any means, but I think the authors could be more explicit about this fact in the discussion, and possibly in the introduction.

My other comments are minor and include the following.

Line 179-180 states that salivary gland replicates consisted of pools of 300 per replicate, but earlier in this section (line 167) it states replicates consisted of 150 per salivary gland replicate. Please clarify the number of glands pooled to constitute one replicate.

Main manuscript has CLas- in red and CLas+ in blue (Fig. 2), while supplementary data uses a different

color scheme (CLas- in black and CLas+ in red). This is somewhat confusing. Consider harmonizing this across main MS and supplemental figures.
The discussion is somewhat hard to follow and jumps back and forth among topic areas. For instance, paragraph one guides the reader to a discussion of CLas reads in different tissues, but then abruptly switches to discussion of ACP-derived reads in the salivary glands and relation of genes expressed to ROS responses. The following paragraph loops back to the discussion of CLas reads in the tissues and focuses on the salivary glands. The paragraph after that jumps to discussion of the heads, then we again return to the salivary glands and discussion of evidence that CLas is replicating at high levels in this tissue. For the sake of clarity, you may want to structure this to collect the information about CLas reads in each tissue into distinct sections so the reader is not forced to go back and forth, then discuss the ACP gene expression patterns in the context of this knowledge about CLas replication activity and the different tissue sources.
The supplementary figures show a fair amount of variation among replicates. Notably, there are two outliers in the salivary gland dataset that were stored in the -80 for longer than the others. Perhaps this warrants more attention in the discussion.
Line 682 - remove comma between "sequencing" and "raw data"

Methods

Are the methods appropriate to the aims of the study, are they well described, and are necessary controls included? Choose an item.

Conclusions

Are the conclusions adequately supported by the data shown? Choose an item.

Reporting Standards

Does the manuscript adhere to the journal’s guidelines on minimum standards of reporting? Choose an item.

Choose an item.

Statistics

Are you able to assess all statistics in the manuscript, including the appropriateness of statistical tests used? Choose an item.

Quality of Written English

Please indicate the quality of language in the manuscript: Choose an item.

Declaration of Competing Interests

Please complete a declaration of competing interests, considering the following questions:

- Have you in the past five years received reimbursements, fees, funding, or salary from an organisation that may in any way gain or lose financially from the publication of this manuscript, either now or in the future?
- Do you hold any stocks or shares in an organisation that may in any way gain or lose financially from the publication of this manuscript, either now or in the future?
- Do you hold or are you currently applying for any patents relating to the content of the manuscript?
- Have you received reimbursements, fees, funding, or salary from an organization that holds or has applied for patents relating to the content of the manuscript?
- Do you have any other financial competing interests?
- Do you have any non-financial competing interests in relation to this paper?

If you can answer no to all of the above, write 'I declare that I have no competing interests' below. If your reply is yes to any, please give details below.

'I declare that I have no competing interests'

I agree to the open peer review policy of the journal. I understand that my name will be included on my report to the authors and, if the manuscript is accepted for publication, my named report including any attachments I upload will be posted on the website along with the authors' responses. I agree for my report to be made available under an Open Access Creative Commons CC-BY license (http://creativecommons.org/licenses/by/4.0/). I understand that any comments which I do not wish to be included in my named report can be included as confidential comments to the editors, which will not be published.

Choose an item.

To further support our reviewers, we have joined with Publons, where you can gain additional credit to further highlight your hard work (see: https://publons.com/journal/530/gigascience). On publication of this paper, your review will be automatically added to Publons, you can then choose whether or not to claim your Publons credit. I understand this statement.

Yes Choose an item.
